# Sleep disorders and associated factors among medical students in the Middle East and North Africa: a systematic review and meta-analysis

**DOI:** 10.1038/s41598-024-53818-2

**Published:** 2024-02-26

**Authors:** Sonia Chaabane, Karima Chaabna, Salina Khawaja, Jasmine Aboughanem, Dhruv Mittal, Ravinder Mamtani, Sohaila Cheema

**Affiliations:** 1 Institute for Population Health, Weill Cornell Medicine – Qatar, Education City, Qatar Foundation, P.O. Box. 24144, Doha, Qatar; 2 Intern, Institute for Population Health, Weill Cornell Medicine – Qatar, Education City, Qatar Foundation, P.O. Box. 24144, Doha, Qatar

**Keywords:** Risk factors, Lifestyle modification, Epidemiology

## Abstract

Sleep disturbances like poor and insufficient sleep are common among medical students in the Middle East and North Africa (MENA) countries; however, the extent of medically defined sleep disorders (SDs) remains unclear. This meta-analysis determines SD prevalence and identifies associated factors among medical students in the MENA. PubMed, Web of Science, Google Scholar, and reference lists of included studies were searched (latest search: June 2022). Meta-analyses included 22 studies and were performed using random-effect models. Included studies used self-reported screening tools for assessing SDs and then estimated the proportion of participants at high risk of developing a SD. Central disorders of hypersomnolence were the most prevalent SD [prevalence_pooled_ range: 30.9% (Jordan) to 62.5% (Saudi Arabia)], followed by insomnia disorders [prevalence_pooled_ range: 30.4% (Jordan) to 59.1% (Morocco)], circadian rhythm sleep–wake disorders [prevalence_pooled_ range: 13.5% (Jordan) to 22.4% (Saudi Arabia)], sleep-related breathing disorders [prevalence_pooled_ range: 12.2% (Jordan) to 22.5% (Pakistan)], sleep-related movement disorders [prevalence_pooled_ range: 5.9% (Egypt) to 30.6% (Saudi Arabia)], and parasomnias [prevalence_pooled_ range: 5.6% (Jordan) to 17.4% (Saudi Arabia)]. Female sex, studying in the latter academic years, having anxiety, excessive internet use, and poor academic performance were significantly associated with SDs. SDs are prevalent among MENA medical students. Implementing student-centered interventions targeting high risk groups in medical schools should be considered to improve students’ health and wellbeing.

## Introduction

Medically defined sleep disorders (SDs) include insomnia disorders, sleep-related breathing disorders, central disorders of hypersomnolence, circadian rhythm sleep–wake disorders, sleep-related movement disorders, and parasomnias^1–4^. SDs increased in the last decade globally^5,6^ but remain under-diagnosed and under-treated^7^. SDs affect not only physical and mental functioning and work productivity but are also associated with various psychiatric^6,8–10^ and physical^11–15^ illnesses, workplace injuries^7^, and sudden death^16^. Consequently, SDs’ substantially impact the society^7^.

Insufficient sleep affects up to one third of the global population^17^ and has been declared a ‘public health epidemic’^18^.Worldwide, medical students appear to be more affected by sleep disturbances (e.g. poor sleep quality, insufficient sleep duration, irregular sleep, and insomnia symptoms) than non-medical students^6,9,19–22^ or the general population^9,19,23–25^, owing to the large academic load and assigned clinical duties^6,26^. Hence, we expect a high SD prevalence in this population.

Several recently published systematic reviews (SRs) are focused on sleep disturbances rather than SDs among medical students^16,20,22–24,27–31^ and university students^19,23,32–36^ Also, the available SRs with data on insomnia were conducted among non-medical university students^9,36–38^ with a focus on one specific country^37^, and the COVID-19 pandemic period^36–38^. Several primary studies on SDs in the Middle East and North Africa (MENA) medical students have been recently published. Notably, the countries of the MENA region have the highest total number of medical schools in their respective continents (Asia and Africa)^39^.

To our knowledge, no SR and meta-analysis synthesizing the epidemiology of SDs in MENA medical students has been conducted. The aim of this SR and meta-analysis was to quantify SD prevalence and synthesize the factors associated with SDs among medical students in the MENA countries before and during the COVID-19 pandemic.

## Methods

The SR methodology was developed based on the Cochrane Handbook for Systematic Review of Interventions and followed the AMSTAR 2 checklist. The manuscript follows the Preferred Reporting Items for Systematic Reviews and Meta-Analyses (PRISMA) guidelines (Table S1), the PRISMA checklist for search strategy (Supplementary Table S2)^40,41^ and Meta-analyses of Observational Studies in Epidemiology (MOOSE) guidelines^42^. The research protocol was developed a priori and registered prospectively on Open Science Framework (https://doi.org/10.17605/OSF.IO/2WZJ4).

### Literature search strategy

PubMed, Web of Science, and Google Scholar were searched by two independent reviewers for grey and non-grey literature. The database selection and search strategy were developed in consultation with a specialized librarian. The latest search was conducted on June 25, 2022. The search included a combination of controlled vocabulary terms and text words related to SDs and medical or university students. The search strategy is described in Supplementary Box S1. Two independent reviewers also manually searched the reference lists of included primary studies and relevant reviews, as well as the internal literature database we developed, titled ‘Mental Health in University Students’.

### Eligibility criteria

#### Primary outcomes

The primary outcome of interest was the prevalence of any SD. We included any SD listed in the International Classification of Sleep Disorders, third edition (ICSD-3): the most widely used classification system and a key reference for the diagnosis of SDs using ‘International Classification of Diseases, Ninth and Tenth Revision, Clinical Modification^1–4^.’ SDs reported in the primary studies were categorized, as per the ICSD-3 recommendations, into seven categories that include: (1) insomnia disorders, (2) sleep-related breathing disorders, (3) central disorders of hypersomnolence, (4) circadian rhythm sleep–wake disorders (CRD), (5) parasomnias, (6) sleep-related movement disorders, and (7) other SDs (not fitting in the previous categories). Specific SDs included in each category as per ICSD-3 are presented in Table S3. SDs identified by clinical diagnosis, or any self-reported tools were included in our SR. Severity levels of SDs were included and categorized into mild, moderate, and severe as per the study and/or instrument definition. Cases of risk of SD were defined as those have abnormal scores (any level) according to the scoring system (cut-offs) recommended by the tool. If not reported, the prevalence of an SD was calculated based on crude data reported in the study. Studies with insufficient information to compute prevalence data of SDs, or those reporting only symptoms related to SDs, were excluded.

#### Secondary outcome

The secondary outcome of interest was the factors associated to a higher risk of SD. We synthesized any effect measure used to quantify a relationship between the factor and SDs reported in the included studies. Reported effect sizes included risk and mean differences, correlations, attributable proportion, risk ratios, relative risks, and odds ratios.

#### Population of interest

A study was eligible for inclusion if it included pre-medical or medical students enrolled in a medical school among the 20 MENA countries: Algeria, Bahrain, Djibouti, Egypt, Iraq, Jordan, Kuwait, Lebanon, Libya, Morocco, Oman, Pakistan, Palestine, Qatar, Saudi Arabia, Sudan, Syria, Tunisia, the United Arab Emirates, and Yemen. The list of MENA countries in this study was based on that developed in a series of published SRs and meta-analyses characterizing the population health MENA^43–51^. Pre-medical and medical students mixed with other university students were included only if data specific to the population of interest was available. We excluded studies on medical science students (nursing, pharmacy, dentistry) unless specified as medical students studying for their Medical Degree (MD or MBBS).

#### Study design

Any observational study (e.g., cross-sectional study, case–control, or cohort) was included in the SR. Reviews, case reports, letters to editors, commentaries, and clinical trials were excluded.

### Multi-stage screening

The Rayyan software (Rayyan Systems, Inc, Cambridge, MA, USA, https://www.rayyan.ai/) was used for duplicate removal and multi-stage screening. Two independent reviewers conducted the title and abstract screening, full-text screening, and data extraction. Discrepancies were resolved with a third reviewer to achieve consensus on study inclusion and data extraction.

For inclusion in the systematic review (SR), a study should correspond to the PICOTS framework criteria^52^—population, outcome, study design, time of the study, and setting (control and intervention criteria were not applicable since they were not relevant to the SR question). We included studies reported in English, Arabic, French, Spanish and/or Urdu—languages spoken by the authors of this SR.

### Data extraction

Data was extracted from the included primary studies for the following variables: (1) study characteristics (e.g. study design, sampling method, data collection period, sample size, (2) setting (3) medical student characteristics including age, sex, year of study, and socio-economic status, (4) prevalence of SDs, including the instrument used to diagnose an SD and its related characteristics, (6) factors for which a difference and/or an association with the risk of having an SD was assessed.

### Quality assessment

The risk of bias (RoB) and methodological quality of included studies were appraised independently by two reviewers using a validated RoB tool for prevalence studies^53^. Briefly, the RoB tool uses an items scale to assess: (1) the external validity of the study, based on selection and nonresponse biases, and (2) the internal validity of the study, based on measurement bias and bias related to the analysis. No summary quality score was computed as per COSMOS-E guidance, which provides guidance on conducting SRs of observational studies of aetiology^54^. Each included study was assigned a low or high ROB for each assessment item. A synthesis of studies’ quality was based on a summary of low and high risk of bias assessment of each quality domain.

Reporting bias due to missing data was discussed. Discussion on the validity and reliability of our estimates was also performed to assess the confidence in the body of evidence presented in the SR. The certainty assessment method was based on the Grading of Recommendations, The Assessment, Development, and Evaluation (GRADE) approach. The GRADE approach used in our study considers the RoB and reporting biases in a body of evidence, precision of the meta-analysis effect estimates, the consistency of the primary study results, and how directly the body of evidence answers the research question^55^.

### Synthesis

A meta-analysis of the prevalence of SD categories (proportion of participants at a high risk of any ICDS category of SD) (Supplementary Table S3) was conducted using the DerSimonian-Laird random-effects model^56^. Random effects model with the logit transformation of the proportion was used to conduct the meta-analyses pooling prevalence measures and their 95% confidence intervals (95% CI). The Freeman-Tukey double arcsine transformation was used in the analyses involving the pooling of proportions, using the command sm = “PFT” in R^57^. Clopper-Pearson confidence intervals were computed for individual prevalence measures. The minimum study sample size required for a study to be included in the meta-analysis was 25^58^.

Subgroup meta-analyses were conducted by sex (males and females), academic training period (preclinical, clinical, and late clinical), and MENA country. For each category of SDs, prevalence data were pooled by SD severity level (mild, moderate, and severe). Prevalence data on mixed disorder levels, mixed sex, and/or mixed training periods were pooled in a separate group. If not reported, the prevalence of an SD was calculated based on crude data reported in the study. Sensitivity analyses were conducted to assess the impact of SD prevalence during the COVID-19 pandemic lockdown on the pooled estimates when applicable. Factors associated with SDs among MENA medical students (secondary outcome) were synthesized as reported in the included studies.

SD prevalence measures stratified by sex, disorder severity level, SDs category, and academic period were prioritized for inclusion in the meta-analysis rather than the overall measures on the entire study population or any SD. Prevalence measures reported for each academic year were combined and classified into preclinical, clinical, and late clinical training periods, according to the medical school curriculum followed by the country. Multiple SDs under the same ICDS category reporting on the same study population were merged prior to the inclusion in the meta-analysis to ensure independency of observations.

The heterogeneity between studies was assessed using the I^2^ statistic^59^ and Cochran’s Q between-subgroups statistic^60^. Heterogeneity between studies was considered as substantial when I^2^ > 50%^61^. The Cochran’s Q between-subgroups statistic was used to test for differences between prevalence estimates across subgroups^60^, and statistical significance was considered at *p* value ≤ 0.05. Univariate random-effects meta-regression was used to estimate odds ratios (ORs) and corresponding 95% confidence interval (CI)s measuring the magnitude of relative changes in the pooled SDs prevalence according to study-level factors^62^.

To further explore heterogeneity between studies, univariate random-effects meta-regression was conducted to evaluate potential associations between SD prevalence and measurable study-level factors, including sampling method, sample size, instrument, and study response rate. Meta regression was used to estimate odds ratios (OR) and corresponding 95% CIs to measure the magnitude of relative changes in the pooled SD prevalence according to the study-level factors^62^.

For the meta regression analyses, all SDs were considered grouped to increase the statistical power.

Both meta-analyses and meta-regressions analyses were conducted using RStudio software (version 2022.07.1 Build 554).

Methodological quality of the included studies was appraised using the risk of bias (RoB) tool for prevalence studies^53^. Reporting bias due to missing data was also discussed. Discussion on the validity and reliability of our estimates was also performed to assess the confidence in the body of evidence presented in the SR.

Publication bias was assessed using the Doi plot, a method that allows better visual representation of asymmetry as compared to the conventional funnel plot^63,64^. In the Doi plot, the effect estimate (X-axis) is plotted against the percentiles converted to a normal quantile (Z-score) for each study (Y-axis). The prevalence of SDs was transformed to the log odds scale for better statistical properties for the meta-analysis^64^. We also estimated LFK index to detect and quantify symmetry of study effects in the Doi plot. A LFK index of zero indicates a complete symmetry. The closer the value of the LFK index to zero, the more symmetrical the Doi plot would be. LFK index, values beyond − 1 and + 1were deemed consistent with asymmetry and potential publication bias^63^. Alternatively, for pooled SDs prevalence with identified publication bias, the 95% prediction interval was used to describe the distribution of true outcome measures around the pooled prevalence^65,66^

## Results

**Figure 1:**
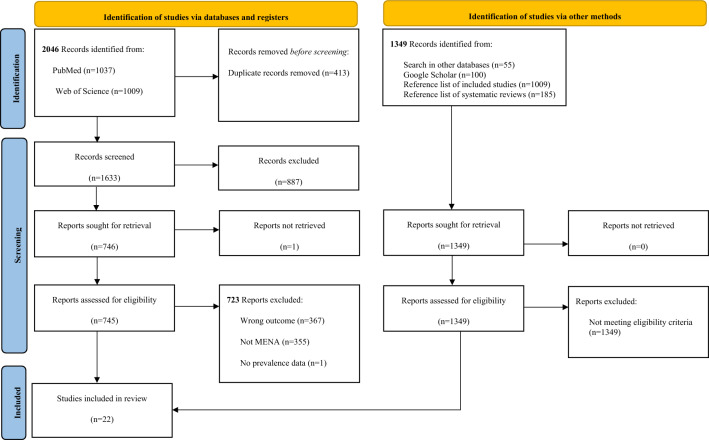
PRISMA flow chart 2020. From: Page MJ, McKenzie JE, Bossuyt PM, Boutron I, Hoffmann TC, Mulrow CD, et al. The PRISMA 2020 statement: an updated guideline for reporting systematic reviews. BMJ 2021;372:n71. https://doi.org/10.1136/bmj.n71. http://www.prisma-statement.org/.

A total of 2046 records were identified through the literature search conducted in PubMed and Web of Science, and 1349 records were identified through Google Scholar, citation hand searching of relevant studies, and other research databases. Twenty-two primary studies were included in the SR and the meta-analysis (Fig. 1). The characteristics of the included studies are described in Table 1. Studies excluded at the full-text screening stage are listed in Supplementary Box S2.

**Table 1:** Characteristics of the included studies on the prevalence of sleep disorders among medical students in MENA.

Country	First author, year—reference	Study design	Sampling method	Data collection time	Population characteristics	Sleep disorder instrument	Prevalence description	Prevalence (%) of students with sleep disorder/issue
Insomnia disorders								
Insomnia								
Pakistan	Pervez^120^	CS	NR	January–April 2019	M: 80 (53.3%), F: 70 (46.7%), Mean age (SD): 23.14 (3.8)	AIS	Any disorder level/any academic period	36.6
							M	31.5
							F	42.8
Pakistan	Zainab^70^	CS	Non-probability	September 2016–December 2017	Liaquat National Medical College, University years: 1–5, M: 111 (30.8%), F: 249 (69%), Mean age (SD): 21.98 (1.7)	ISI	Any disorder level/any academic period	17
Pakistan	Khan^121^	CS	Non-probability	June–August 2018	University of Lahore, University years: 1–5, M: 106 (48.2%), F: 114 (51.8%)	ISI	Any disorder level/any academic period	51.8
							F	75
Pakistan	Khurshid^122^	Longitudinal	NR	2019	Local College of Lahore, Sample size: 309	Self-developed questionnaire	Any disorder level/any academic period	9.7
Pakistan	Ali^123^	CS	Non-probability	March–April 2019	M: 350 (70%), F: 150 (30%)	NR	Any disorder level/any academic period	75.2
Pakistan	Shakeel^124^	CS	Probability	February–May 2018	University years: 3–6, M: 75 (55.55%), F: 60 (44.44%), Mean age (SD): 21 (6.9)	AIS	Any disorder level/clinical training period	59.25
							M	41.81
							F	58.18
Pakistan	Ram^125^	CS	Non-probability	NR	University years: 1–5, M: 35 (10.8%), F: 290 (89.2%), Mean age (SD): 20.55 (1.67)	AIS	Any disorder level/any academic period	33.5
							M	40
							F	32.8
Saudi Arabia	Mohamed^75^	CS	Probability	NR	Medical students at Majmaah University, University years: 1–5, M: 145 (76.3%), F: 45 (23.7%)	NR	Any disorder level/any academic period	70
							Mild	48.9
							Moderate	17
							Severe	17
Saudi Arabia	Alfadeel^126^	CS	Non-probability	2015–2016	College of Medicine at Almaarefa colleges for science and technology (MCST), University years: 1–3, F: 150 (100%)	AIS	Pre-clinical	74
							Clinical	72.8
							Late clinical	76.6
Saudi Arabia	Alrashed^68^	CS	Non-probability	During COVID-19	Medical students at King Saud University (KSU), University years: 3–5 M: 256 (55.3%), F: 207 (44.7%)	ISI	Any disorder level/any academic period	35
							M	43
							F	57
							Year 3	36
							Year 4	36.51
							Year 4–5	28.92
							Year 5	16.7
							Intern	20.9
Saudi Arabia	Mansour^127^	CS	Non-probability	October 2013–2014	College of Medicine, Taibah University, M: 38 (31.1), F: 84 (68.9), Mean age (SD): 20.5	Self-developed questionnaire	Any disorder level/any academic period	23.7
							M	10.5
							F	29.7
Saudi Arabia	Alzahrani^128^	CS	Probability	Total duration including data collection took around 6 months for completion of the study	Majmaah University, M: 98 (75.4), F: 32 (24.6), University years: 2–5	Self-developed questionnaire	Any disorder level/any academic period	37.7
							M	38.7
							F	34.3
Saudi Arabia	Goweda^129^	CS	Probability	During the ongoing global COVID-19 crisis and lockdown, February 10–1 April 2020	Umm Al-Qura University Faculty of Medicine, Bachelor of Medicine, Bachelor of Surgery (MBBS), University years: 2–6, M: 217 (49.5%), F: 221 (50.5%)	SLEEP-50	Any disorder level/any academic period	31.5
Saudi Arabia	Alshaaer^73^	NR	Non-probability sampling	NR	Alfaisal University College of Medicine University years: 2–3, Sample size: 129	AIS	Any disorder level/any academic period	62.1
Jordan	Alqudah^130^	CS	Non-probability	NR	University years: 1- 6, M/F, Sample size: 299	ISI	Any disorder level/any academic period	70.2
Jordan	Yassin^71^	CS	Probability	Academic year 2018–2019	Jordan University of Science and Technology/Yarmouk University, M: 493 (47.4%), F: 548 (52.6%)	SLEEP-50	Any disorder level/any academic period	18.3
							M	17.8
							F	18.8
Jordan	Al-mistarehi^72^	NR	NR	NR	Jordan University of Science and Technology in Irbid, M: 410 (48.5%), F: 436 (51.5%), Mean age (SD): 22.4 (2.1)	SLEEP-50	Any disorder level/academic period NR	18.7
Morocco	Essangri^69^	CS	Probability	April 8–18, 2020, During COVID	All medical universities in Morocco, University years: 1–8, M: 143 (26%), F: 406 (74%)	ISI	Mild	28.8
							Moderate	26.8
							Severe	7.1
							M	50.3
							F	67
							Pre-clinical	67.6
							Clinical	65.5
							Late clinical	49.1
Sleep state misperception (paradoxical insomnia)								
Jordan	Yassin^71^	CS	Probability	Academic year 2018–2019	Jordan University of Science and Technology/Yarmouk University, M: 493 (47.4%), F: 548 (52.6%)	SLEEP-50	Any disorder level/any academic period	0.57
							M	0.6
							F	0.5
Sleep-related movement disorders								
Restless leg syndrome/periodic limb movement disorder								
Saudi Arabia	Goweda^129^	CS	Probability	During the ongoing global COVID-19 crisis and lockdown, February 10–1 April 2020	Umm Al-Qura University Faculty of Medicine, Bachelor of Medicine, Bachelor of Surgery (MBBS), University years: 2–6, M: 217 (49.5%), F: 221 (50.5%)	SLEEP-50	Any disorder level/any academic period	22.4
Saudi Arabia	Burhan^76^	CS	NR	February–May 2017	King Abdulaziz University, Sample size: 618, M/F	SLEEP-50	Any disorder level/academic period NR	39.29
Pakistan	Ishaq^131^	CS	Non-probability sampling	June 2017–July 2018	Jinnah Medical and Dental Medical College (JMDC) DIMC), Liaquat College of Medicine and Dentistry (LCMD), and Al-Tibri Medical College (ATMC)), Karachi, M: 80 (26.6%), F: 220 (73.3%), Mean age: 21.3 (1.68)	RLS rating scale	Any disorder level/academic period NR	7
							M	9
							F	6
							Mild	3
							Moderate	4
Jordan	Al-Mistarehi^72^	NR	NR	NR	Jordan University of Science and Technology in Irbid: M: 410 (48.5%), F: 436 (51.5%), Mean age (SD): 22.4 (2.1)	SLEEP-50	Any disorder level/academic period NR	14.2
Jordan	Yassin^71^	CS	Probability	Academic year 2018–2019	Jordan University of Science and Technology or Yarmouk University, M: 493 (47.4%), F: 548 (52.6%)	SLEEP-50	Any disorder level/any academic period	10.3
							M	7.3
							F	13.1
Egypt	Shalash^132^	CS	Non-probability	January–November 2013	Faculty of Medicine, Ain Shams University Cairo, M: 172 (44.2%), F: 217 (55.8%)	RLS rating scale	Moderate disorder level/any academic period NR	5.9
							M	7.5
							F	4.6
Sleep-related breathing disorders								
Obstructive sleep apnea								
Saudi Arabia	Goweda0^129^	CS	Probability	During the ongoing global COVID-19 crisis and lockdown, February 10–1 April 2020	Umm Al-Qura University Faculty of Medicine, Bachelor of Medicine, Bachelor of Surgery (MBBS), University years: 2–6, M: 217 (49.5%), F: 221 (50.5%)	SLEEP-50	Any disorder level/any academic period	16.4
Saudi Arabia	Burhan^76^	CS	NR	February–May 2017	King Abdulaziz, University, Sample size: 618	SLEEP-50	Any disorder level/academic period NR	26.81
Jordan	Yassin^71^	CS	Probability	Academic year 2018–2019	Jordan University of Science and Technology/Yarmouk University, M: 493 (47.4%), F: 548 (52.6%)	SLEEP-50	Any disorder level/any academic period	12.1
							M	15.4
							F	9.1
Jordan	Al-Mistarehi^72^	NR	NR	NR	Jordan University of Science and Technology in Irbid, M: 410 (48.5%), F: 436 (51.5%), Mean age (SD): 22.4 (2.1)	SLEEP-50	Any disorder level/academic period NR	12
Pakistan	Zainab^70^	CS	Non-probability	September 2016–December 2017	Liaquat National Medical College, University years: 1–5, M: 111 (30.8%), F: 249 (69%), Mean age (SD): 21.98 (1.7)	BQ	Any disorder level/any academic period	20.6
Central disorders of hypersomnolence								
Hypersomnia								
Jordan	Yassin^71^	CS	Probability	Academic year 2018–2019	Jordan University of Science and Technology/Yarmouk University, M: 493 (47.4%), F: 548 (52.6%)	SLEEP-50	Any disorder level/any academic period	23
							M	22.1
							F	23.9
Narcolepsy								
Saudi Arabia	Burhan, 2019^76^	CS	NR	February–May 2017	King Abdulaziz University, Sample size: 618, M/F	SLEEP-50	Any disorder level/academic period NR	72.64
Saudi Arabia	Goweda^129^	CS	Probability	During the ongoing global COVID-19 crisis and lockdown, February 10–1 April 2020	Umm Al-Qura University Faculty of Medicine, Bachelor of Medicine, Bachelor of Surgery (MBBS), University years: 2–6, M: 217 (49.5%), F: 221 (50.5%)	SLEEP-50	Any disorder level/any academic period	51.6
Jordan	Yassin^71^	CS	Probability	Academic year 2018–2019	Jordan University of Science and Technology/Yarmouk University, M: 493 (47.4%), F: 548 (52.6%)	SLEEP-50	Any disorder level/any academic period	7.8
							M	8.7
							F	7.1
Circadian rhythm sleep–wake disorders								
Circadian rhythm disorder								
Saudi Arabia	Goweda^129^	CS	Probability	During the ongoing global COVID-19 crisis and lockdown, February 10–1 April 2020	Umm Al-Qura University Faculty of Medicine, Bachelor of Medicine, Bachelor of Surgery (MBBS), University years: 2–6, M: 217 (49.5%), F: 221 (50.5%)	SLEEP-50	Any disorder level/any academic period	22.4
Jordan	Al-mistarehi^72^	NR	NR	NR	Jordan University of Science and Technology in Irbid, M: 410 (48.5%), F: 436 (51.5%), Mean age (SD): 22.4 (2.1)	SLEEP-50	Any disorder level/academic period NR	13.9
Jordan	Yassin, 2020^71^	CS	Probability	Academic year 2018–2019	Jordan University of Science and Technology/Yarmouk University, M: 493 (47.4%), F: 548 (52.6%)	SLEEP-50	Any disorder level/any academic period	13.2
							M	14.8
							F	11.9
Parasomnias								
Nightmares								
Saudi Arabia	Goweda^129^	CS	Probability sampling	During the ongoing global COVID-19 crisis and lockdown, February 10–1 April 2020	Umm Al-Qura University Faculty of Medicine, Bachelor of Medicine, Bachelor of Surgery (MBBS), University years: 2–6, M: 217 (49.5%), F: 221 (50.5%)	SLEEP-50	Any disorder level/any academic period	13.7
Jordan	Yassin^71^	CS	Probability	Academic year 2018–2019	Jordan University of Science and Technology/Yarmouk University, M: 493 (47.4%), F: 548 (52.6%)	SLEEP-50	Any disorder level/any academic period	4.6
							M	3
							F	6
Sleep walking								
Saudi Arabia	Goweda^129^	CS	Probability	During the ongoing global COVID-19 crisis and lockdown, February 10–1 April 2020	Umm Al-Qura University Faculty of Medicine, Bachelor of Medicine, University years: 2–6, Bachelor of Surgery (MBBS), M: 217 (49.5%), F: 221 (50.5%)	SLEEP-50	Any disorder level/any academic period	3.7
Jordan	Yassin^71^	CS	Probability	Academic year 2018–2019	Jordan University of Science and Technology/Yarmouk University, M: 493 (47.4%), F: 548 (52.6%)	SLEEP-50	Any disorder level/any academic period	1
							M	1.6
							F	0.5
Any sleep disorders								
Saudi Arabia	Almansour^133^	CS	Probability	NR	College of Medicine at King Saud University in Riyadh, University years: 1–5, M: 215 (50%), F: 215 (50%)	Self-developed questionnaire	Any disorder level/any academic period	9.5*

*Calculated based on crude data.

*M* male, *F* female, *SD* Standard deviation, *CS* cross-sectional study, *RLS rating scale* restless leg syndrome rating scale, *REM* rapid eye movement, *NREM* non-rapid eye movement, *ISI* insomnia severity index, *AIS* athens insomnia scale, *BQ* berlin questionnaire, *NR* not reported.

*Considered cut-offs for seep disorders* RLS rating scale: Mild (1–10 points), Moderate (11–20 points); Severe (21–30 points); Very severe (31–40 points); Qualified individuals (meeting the International Restless Legs Syndrome Study Group (IRLSSG) criteria for the diagnosis of Restless Legs Syndrome (RLS)) to take this scale should have shown symptoms). ISI: Subthreshold insomnia (8–14 points); Clinical insomnia (moderate severity) (15–21 points); Clinical insomnia (severe) (22–28 points). AIS: Mild insomnia (6–9 points); Moderate insomnia (10–15), Severe insomnia (16–24). BQ: High risk for Obstructive Sleep Apnea (OSA) if two or more sections out of the three sections have positive scores.

### Characteristics of the included studies

Out of the included studies, 21 were cross-sectional (95%, 21/22) and one study used a longitudinal design. They were conducted in Egypt, Jordan, Morocco, Pakistan, and Saudi Arabia, between 2013 and 2019, and published between 2015 and 2021. The MENA medical students’ mean age ± standard deviation ranged between 20.5 ± 1.67 and 23.1 ± 3.8 years. The total population size varied between 122 and 1,041 medical students (Table 1).

### Pooled estimates of sleep disorders

#### Insomnia disorders

A total of 54 prevalence measures classified under insomnia disorders, including sleep state misperception (paradoxical insomnia), were retrieved from 18 studies conducted in Jordan, Morocco, Pakistan, and Saudi Arabia (Table 1). Following data merging and classification, 30 prevalence measures on any type of insomnia disorder were included in the meta-analysis. Most of the included data (24/30 prevalence measures) were computed from any severity level of insomnia.

The pooled prevalence of insomnia disorders ranged between 30.4% in Jordan and 59.1% in Morocco. A total of three studies^67–69^ were conducted during the COVID-19 pandemic lockdown in Saudi Arabia^67,68^ and Morocco^69^. In Saudi Arabia, pooled prevalence computed with and without data during the COVID-19 pandemic lockdown were similar, at 45.9%; 95% CI: 30.2–62.1 and 50.0%; 95% CI: 29.4–70.6 respectively. The pooled prevalence of insomnia disorders, in Saudi Arabia and Pakistan, following the exclusion of the study not reporting the used tool was 42.1% 95% CI: 27.1–57.9 and 36% 95% CI: 23.1–50.0, respectively. All prevalence data identified for Morocco was measured during the COVID-19 pandemic lockdown.

No statistically significant difference in the prevalence of insomnia disorders was identified between MENA countries. In these countries, mild-level of insomnia was reported by 38.4% of medical students, followed by moderate- (22.2%) and severe-levels of insomnia (11.6%) (Table 2). Insomnia disorders were significantly more prevalent among female medical students than among male students (49.9% vs. 26.1%; *p* value = 0.0027). Although the highest pooled prevalence of insomnia disorders was observed during the late clinical training period (year 7 or more), no statistically significant difference was found between the academic training periods.

**Table 2:** Meta-analysis of sleep disorders prevalence among medical students in MENA countries.

	Number of prevalence measures	Total sample size	Prevalence range (%)	Effect size		Subgroup Comparison (Q between subgroup tests *p* value)		Heterogeneity between studies I^2^ (%)
				Weighted average prevalence (%)	95% CI			
Insomnia disorders								
Countries								
Jordan	4	2186	18.5–70.2	30.4	9.8–56.5	0.222		99.0
Morocco	2	549	67.0–50.3	59.1	42.5–74.7			91.8
Saudi Arabia	12	1772	9.2–83.7	45.9	30.2–62.1			97.4
Pakistan	10	1913	9.7–75.2	40.2	26.1–55.2			98.5
Sex								
M	8	1218	9.2–50.3	26.1	16.7–36.6	0.003	< 0.001	91.5
F	12	2111	19.3–74.0	49.9	38.5–61.3			97.2
M/F not separated	7	2731	18.7–83.7	51.3	29.9–72.5	NA		99.3
N/R	1	309	9.7	9.7	6.6–13.6			N/A
Training period								
Preclinical	4	680	25.5–74.0	57.3	35.2–77.9	0.467	< 0.001	97.6
Clinical	5	688	30.7–72.8	52.0	35.8–67.9			94.3
Late clinical	3	208	49.2–79.1	68.1	48.1–85.3			89.0
Mixed training periods	12	3417	9.2–75.2	41.8	26.3–58.1	NA		98.9
NR	6	1427	9.7–42.9	22.6	13.1–33.7			90.9
Disorder severity level*								
Mild	2	739	28.8–48.9	38.4	20.0–58.7	0.050	0.003	98.6
Moderate	2	739	17.4–26.8	22.2	13.8–32.0			95.9
Severe	2	739	7.1–17.4	11.6	3.5–23.4			86.0
Level not specified	24	5531	9.2–76.6	37.9	28.7–47.6	NA		93.2
Sleep-related breathing disorders								
Countries								
Jordan	3	1887	9.1–15.4	12.2	8.9–15.9	0.001	79.4	
Saudi Arabia	2	1056	16.4–26.9	21.5	12.2–32.5		93.9	
Pakistan	1	329	22.5	22.5	18.1–27.4		N/A	
Sex								
M	1	493	15.4	15.4	12.3–18.9	0.002	< 0.001	N/A
F	1	548	9.1	9.1	6.8–11.9			N/A
M/F not separated	4	3272	12.4–26.9	19.2	13.2–26.0	NA		94.4
Training period								
Mixed training periods	4	1808	9.1–22.5	15.5	10.4–21.3	0.636	90.3	
NR	2	1464	12.4–26.9	19.1	7.1–35.0		97.9	
Disorder severity level*								
Level not specified	6	3272	9.1–26.9	16.7	11.8–22.2	NA	94.2	
Central disorders of hypersomnolence								
Countries								
Jordan	2	1041	30.8–31.0	30.9	28.2–33.8	0.004	0.0	
Saudi Arabia	2	1056	51.6–72.7	62.5	41.2–81.5		98.0	
Sex								
M	1	493	30.8	30.8	26.8–35.1	0.015	NA	
F	1	548	31.0	31.0	27.2–35.1		NA	
M/F not separated	2	1056	51.6–72.7	62.5	41.2–81.5		98.0	
Training period								
Mixed training periods	3	1479	30.8–51.6	37.6	24.9–51.3	< 0.001	96.4	
NR	1	618	72.7	72.7	69.0–76.1		NA	
Disorder severity level*								
Level not specified	3	2097	30.8–72.7	46.5	27.2–66.4	NA	99.0	
Circadian rhythm sleep–wake disorders								
Countries								
Jordan	3	1887	11.9–14.8	13.5	12.0–15.1	< 0.001	6.7	
Saudi Arabia	1	438	22.4	22.4	18.6–26.6		NA	
Sex								
M	1	493	14.8	14.8	11.8–18.3	0.202	NA	
F	1	548	11.9	11.9	9.3–14.9		NA	
M/F not separated	2	1284	13.9–22.4	17.9	10.4–26.8		92.9	
Training period								
Mixed training periods	3	1479	11.9–22.4	16.1	10.6–22.5	0.502	89.9	
NR	1	846	13.9	13.9	11.7–16.5		NA	
Disorder severity level*								
Level not specified	4	2325	11.9–22.4	15.5	11.5–20.0	0.000	85.9	
Parasomnias								
Countries								
Jordan	2	1041	4.7–6.6	5.6	3.9–7.6	< 0.001	42.5	
Saudi Arabia	1	438	17.4	17.4	13.9–21.2		NA	
Sex								
M	1	493	4.7	4.7	3.0–6.9	< 0.001	NA	
F	1	548	6.6	6.6	4.6–9.0		NA	
M/F not separated	1	438	17.4	17.4	13.9–21.2		NA	
Training period								
Mixed training periods	3	1479	4.7–17.4	8.8	3.0–17.3	NA	95.6	
Disorder severity level*								
Level not specified	3	1479	4.7–17.4	8.8	3.0–17.3	NA	95.6	
Sleep-related movement disorders								
Countries								
Jordan	3	1887	7.3–14.2	11.4	7.4–16.1	0.002	87.9	
Saudi Arabia	2	1056	22.4–39.3	30.6	15.5–48.2		97.1	
Pakistan	2	300	6.4–8.8	6.9	4.2–10.1		0.0	
Egypt	2	389	4.6–7.6	5.9	3.3–9.1		32.1	
Sex								
M	3	745	7.3–8.8	7.4	5.6–9.4	0.897	0.024	0.0
F	3	985	4.6–13.1	7.9	3.6–13.5			88.6
M/F not separated	3	1902	14.2–39.3	24.6	11.8–40.1	NA		98.4
Training period								
Mixed training periods	3	1479	7.3–22.4	13.7	6.4–23.2	0.818	95.5	
NR	6	2153	4.6–39.3	12.1	4.6–22.4		97.9	
Disorder severity level*								
Mild	1	300	3.0	3.0	1.4–5.6	0.002	NA	
Moderate	3	689	4.0–7.6	5.1	3.3–7.2		27.3	
Level not specified	5	2943	7.3–39.3	18.2	9.0–29.6		98.2	

*NR* not reported, *NA* not applicable.

Weighted average prevalence measures were obtained using random-effect model. p-value ≤ 0.05 was considered statistically significant. The total number of prevalence measures in each category of sleep disorders (SDs) may vary according to the subgroup analysis.

*Severity disorder levels were considered as classified by the individual studies and are mutually exclusive. For disorder severity levels, the ’level not specified’ category includes SD prevalence measures reported without any specification of the disorder severity level from the primary studies.

Pooled prevalence measures by sex consider combined disorder severity levels, academic training periods, and countries for a specific SDs.

Pooled prevalence measures by academic training period consider combined disorder severity levels, sexes, and countries for a specific SDs.

Pooled prevalence measures by MENA countries consider combined SDs severity levels, sexes, and academic training periods. For sex variable, ‘M/F not separated’ includes studies where SD prevalence was not provided for males and females separately.

Reported factors significantly associated with an increased odds of having insomnia disorders were being a female student^69^, clinical or late clinical years^69^, and use of internet for more than 12 h daily^70^ (Table 3). The reported findings also highlighted the significant negative impact of insomnia^70–73^ and sleep state misperception disorder^71^ (known also as paradoxical insomnia^74^) on academic performance. Reported data suggests that the impact of anxiety on the risk of insomnia depends on the anxiety severity level^70,75^.

**Table 3:** Synthesis of reported factors associated with the risk of sleep disorders among MENA medical students.

Factors	Compared groups^d^	Mean ± Standard Deviation (SD)/OR/r coefficient	*p* value/95% CI
Insomnia disorders			
Insomnia			
Sex	**Female versus Male**^69^	**OR**^**a**^**: 1.830**	**95% CI: 1.176–2.847, *p*-value = 0.007**
	**Male versus Female**^70^	OR adjusted^a^: 1.42	95% CI: 0.71–2-83
Age	**“There was increase in the frequency of insomnia with increased age”**^125^	**NR**	***p*-value = 0.008**
Academic Year	**Preclinical Year (1st-2nd year) versus Early (3rd-6thyear) & Late clinical year (> 7 years)**^69^	**OR**^**a**^**: 0.720**	**95% CI: 0.545–0.949, p-value = 0.020**
	2nd year versus 1st year^70^	OR adjusted^a^: 2.54	95% CI: 0.60–10.67
	3rd year versus 1st year^70^	OR adjusted^a^: 1.76	95% CI: 0.33–8.76
	5th year versus 1st year^70^	OR adjusted^a^: 1.32	95% CI: 0.17–10.09
Academic performance	**Low mean GPA (< 3.0) versus High mean GPA (> 3.0)**^72^	**NR**	***p*-value = 0.001**
		**OR**^**a**^**: 1.59**	**95% CI: 1.11–2.28, p-value = 0.012**
	**Students’ GPA**^73^	**R = − 0.163**	***p*-value = 0.03**
	**Perceived very frequently their academic performance affected by insomnia versus ‘do not perceive at all their academic performance affected by insomnia**^70^	**OR adjusted**^a^**: 5.01**	**95% CI: 1.69–14.82**
	Perceived frequently their academic performance affected by insomnia versus do not perceive at all their academic performance affected by insomnia^70^	OR adjusted^a^: 2.16	95% CI: 0.71–6.60
	“Perceived sometimes their academic performance affected by insomnia” versus “do not perceive at all their academic performance affected by insomnia^70^	OR adjusted^a^: 1.30	95% CI: 0.48–3.52
	**Poor academic performance (GPA ≤ 2.49) versus Good academic per (GPA ≥ 2.50)**^71^	**OR adjusted**^**c**^**: 1.96**	**95% CI: 1.35–2.85, p-value < 0.001**
Technology use	**Internet use more than 12 h daily versus Less than 4 to 8 h**^70^	**OR adjuste**d^a^:** 5.20**	**95% CI: 1.66–16.26, *p*-value = < 0.010**
Characteristics of residency	Living in high prevalence of Covid versus Low prevalence of Covid^69^	OR^a^: 1.370	95% CI: 0.931–2.018, *p*-value = 0.111
Initial exposure followed by a subsequent experience of dissection	**No Insomnia versus Insomnia **^122^	**90.3%versus 9.7%**	***p*-value = 0.001**
Stress	Mild stress versus no stress^70^	OR adjusted^a^: 0.79	95% CI: 0.27–2.30
	Moderate stress versus no stress^70^	OR adjusted^a^: 1.77	95% CI: 0.65–4.84
	Severe stress versus no stress^70^	OR adjusted^a^: 1.24	95% CI: 0.28–5.42
	Extremely severe stress versus no stress^70^	OR adjusted^a^: 7.18	95% CI: 0.90–17.00
Anxiety	Mild anxiety versus no anxiety^70^	OR adjusted^a^: 0.55	95% CI: 0.17–1.77
	Moderate anxiety versus no anxiety^70^	OR adjusted^a^: 1.88	95% CI: 0.70–5.03
	Severe anxiety versus no anxiety^70^	OR adjusted^a^: 0.61	95% CI: 0.15–2.43
	Extreme severe anxiety versus mild, moderate, severe, or no anxiety^70^	OR adjusted^a^: 1.95	95% CI: 0.50–7.57
	**Mild versus Moderate versus Severe anxiety**^75^	**50% versus 72.1% versus 91.5%**	***p*-value < 0.001**
Sleep state misperception (paradoxical insomnia)			
Academic performance	**Poor academic performance (GPA ≤ 2.49) versus good academic performance (GPA ≥ 2.50)**^71^	**OR adjusted**^**c**^**: 6.40**	**95% CI: 1.04–39.19, *p*-value = 0.045**
Sleep-Related Breathing Disorders			
Obstructive sleep apnea			
Sex	**Male versus Female**^71^	**OR unadjusted: 1.815**	**95% CI: 1.242–2.654, *p*-value = 0.002**
	**Male versus Female**^70^	**OR adjusted** ^a^**: 3.52**	**95% CI: 1.85–6.66, *p*-value = < 0.001**
Age	**One year increase in the age**^70^	**OR adjusted**^a^**: 1.37**	**95% CI: 1.01–1.85**
Academic performance	**Low mean GPA (< 3.0) versus High mean GPA (> 3.0)**^72^	**OR**^**a**^**:1.57**	**95% CI: 1.03–2.39**
	Poor academic performance (GPA ≤ 2.49) versus Good academic performance (GPA ≥ 2.50)^71^	16.8% versus 11.0%	*p*-value = 0.117
Academic year	3rd year versus 1st Year^70^	OR adjusted^a^: 2.61	95% CI: 0.56–12.14
	2nd year versus 1st Year^70^	OR adjusted^a^ 1.19	95% CI: 0.26–5.09
Use of technology	**Use of internet for** **4–8 h daily versus < 4–8 h daily**^70^	**OR adjusted**^a^**: 2.41**	**95% CI: 1.14–5.08**
Stress	Mild stress versus no stress^70^	OR adjusted^a^: 0.55	95% CI: 0.20–1.47
	**Moderate stress versus no stress**^70^	**OR adjusted**^a^** 0.21**	**95% CI: 0.06–0.74, *p*-value ≤ 0.050**
	Severe stress versus no stress^70^	OR adjusted^a^: 0.75	95% CI: 0.15–3.73
	Extremely severe stress versus no stress^70^	OR adjusted^a^: 0.22	95% CI: 0.02–1.77
Anxiety	**Mild anxiety versus no anxiety**^70^	**OR adjusted**^a^**: 3.44**	**95% CI: 1.47–8.07, *p*-value ≤ 0.010**
	Moderate anxiety versus no anxiety^70^	OR adjusted^a^: 1.38	95% CI: 0.45–4.20
	Severe anxiety versus no anxiety^70^	OR adjusted^a^: 1.69	95% CI: 0.47–5.99
	**Extremely severe anxiety versus no anxiety**^70^	**OR adjusted**^a^**: 32.01**	**95% CI: 7.45–137.42, *p*-value ≤ 0.001**
Central Disorders of Hypersomnolence			
Hypersomnia			
Academic performance	Poor academic performance (GPA ≤ 2.49) versus Good Academic per (GPA ≥ 2.50)^71^	19.4% versus 23.9%	*p*-value = 0.176
Narcolepsy			
Academic performance	**Poor academic performance (GPA ≤ 2.49) versus Good academic performance (GPA ≥ 2.50)**^71^	**OR adjusted**^**c**^**: 2.03**	**95% CI: 5.83–15.60, p-value = 0.045**
Circadian Rhythm Sleep–Wake Disorders			
Circadian Rhythm Sleep–Wake Disorders			
Academic performance	**Poor academic performance (GPA ≤ 2.49) versus good academic performance (GPA ≥ 2.50)**^71^	**OR adjusted**^**c**^**: 2.03**	**95% CI: 1.34–3.08, *p*-value < 0.001**
	**Low mean GPA (< 3.0) versus High mean GPA (> 3.0)**^72^	**OR**^**a**^**: 1.71**	**95% CI: 1.50–2.56, p-value = 0.008**
Parasomnias			
Nightmares			
Sex	**Male versus Female**^71^	**OR unadjusted: 0.490**	**95% CI: 0.263–0.913, *p*-value = 0.022**
Academic performance	Poor academic performance (GPA ≤ 2.49) versus Good academic performance (GPA ≥ 2.50)^71^	5.6% versus 4.4%	*p*-value = 0.458
Sleep walking			
Academic performance	Poor academic performance (GPA ≤ 2.49) versus good academic performance (GPA ≥ 2.50)^71^	2.0% versus 0.8%	*p*-value = 0.135
Sleep-Related Movement Disorders			
Periodic limb movement disorder/restless leg syndrome			
Sex	RLS score in Female versus Male^131^	Mean ± SD: 10.86 ± 1.34 & Mean ± SD: 11.57 ± 5.798	*p*-value = 0.754
	**Male versus Female**^71^	**OR unadjusted: 0.521**	**95% CI: 0.342–0.793, *p*-value = 0.002**
Academic performance	Poor academic performance (GPA ≤ 2.49) versus Good academic performance (GPA ≥ 2.50)^71^	11.2% versus 10.2%	*p*-value = 0.665
	“RLS/PLMD was not associated with low academic performance (GPA < 3.0)”^72^	NR	*p*-value = 0.152
Combined categories of SDs^e^			
Academic performance	GPA score in students with SDs versus GPA score in students without SDs^67^	Mean ± SD: 2.72 ± 1.34 versus Mean ± SD: 2.61 ± 1.43	*p*-value = 0.484
	**Poor academic performance (GPA ≤ 2.49) versus Good (GPA 2.50–2.99) versus Very good (GPA 3.00–3.49) versus Excellent (GPA 3.50–3.99)/Outstanding (GPA ≥ 4.00) academic performance**^71^	**93.4% versus 82.9%** **versus 66.8% versus 56.5%**	***p*-value = 0.005**
Use of Technology	**Time spent watching television and/or on smartphones versus No time spent watching television and/or on smartphones**^67^	**Mean ± SD: 6.71 h ± 3.83 versus Mean ± SD: 5.90 h ± 3.40**	***p*-value = 0.004**
BMI (BMI kg/m^2^)	BMI in SD group versus BMI in no SD group^67^	Mean ± SD: 24.8 kg/m2 ± 6.45 versus Mean ± SD: 24.8 kg/m2 ± 7.50	*p*-value = 0.936

Significant results are highlighted in bold (*p* value ≤ 0.05).

*GPA* grade point average, *OR* odd ratio, *NR* not reported, *BMI* body max index, *RLS* restless leg syndrome.

^a^Adjustment unknown;

^b^Model included age, gender, education, stress, anxiety, use of internet, and academic performance;

^c^Adjusted for sex and obesity;

^d^The second group listed refers to the reference group when applicable.

^**e**^The studies assessed the factor associated with SDs combining obstructive sleep apnea (OSA), Insomnia, Narcolepsy, Restless leg syndrome/Periodic limb movement disorder (RLS/PLMD), Circadian rhythm disorder (CRD), Sleep walking, and Nightmares.

#### Sleep-related breathing disorders

A total of 7 prevalence measures classified under sleep-related breathing disorders, including obstructive sleep apnoea (OSA) disorders, were retrieved from 5 studies conducted in Jordan, Pakistan, and Saudi Arabia (Table 1). Following data merging and classification, 6 prevalence measures on any type of sleep-related breathing disorders were included in the meta-analysis.

The pooled prevalence of sleep-related breathing disorders ranged between 12.2% in Jordan and 22.5% in Pakistan (Table 2). Significant differences in the pooled prevalence of these disorders were identified between Jordan, Pakistan, and Saudi Arabia. Only one study^67^ from Saudi Arabia reported a prevalence of Obstructive Sleep Apnea (OSA) during the COVID-19 pandemic lockdown. Statistically significant difference in the pooled prevalence of sleep-related breathing disorders was also found between sexes (*p* value = 0.002); however, this observation was based on a limited number of data points. Reported factors significantly associated with an increased odds of having OSA were being a male student^70,71^, increased age^70^, progression through the academic years^76^, use of the internet for more than 4–8 h daily^70^, and mild and extremely severe anxiety^70^ (Table 3). Moderate stress was significantly associated with lower odds of having OSA when compared with no stress^70^.

#### Central disorders of hypersomnolence

A total of 8 prevalence measures classified under central disorders of hypersomnolence were retrieved from 3 studies, including hypersomnia and narcolepsy disorders, conducted in Jordan and Saudi Arabia (Table 1).

The pooled prevalence of central disorders of hypersomnolence ranged between 30.9% in Jordan and 62.5% in Saudi Arabia (Table 2). Significant differences in the prevalence of central disorders of hypersomnolence were found between these two countries. Only one study reported prevalence data of central disorders of hypersomnolence by sex^71^, and another one^67^ during the COVID-19 pandemic lockdown in Saudi Arabia. Reported risk of having narcolepsy was significantly associated with an increased odd of having poor academic performance^71^ (*p* value = 0.045) (Table 3). No significant differences were reported for the prevalence of narcolepsy and hypersomnia between males and females^71^.

#### Circadian rhythm sleep–wake disorders (CRD)

A total of 5 prevalence measures classified under CRD disorders were retrieved from 3 studies conducted in Jordan and Saudi Arabia (Table 1). Following data merging and classification, 5 prevalence measures on any type of CRD disorder were included in the meta-analysis.

A significant difference in the pooled prevalence of CRD was found between the two countries with available data: Jordan (13.5%) and Saudi Arabia (22.4%) (Table 2). Only one study reported prevalence data of CRD by sex^71^. and another one^67^ during the COVID-19 pandemic lockdown in Saudi Arabia. Reported risk of having CRD was associated with an increased odds of having poor academic performance in two studies^71,72^ (Table 3). No significant difference was reported in the prevalence of CRD between males and females (*p* value = 0.162)^71^.

#### Parasomnias

A total of 8 prevalence measures classified under parasomnias disorders, including nightmares and sleep walking, were retrieved from 2 studies conducted in Jordan and Saudi Arabia (Table 1). Following data merging and classification, 3 prevalence measures on any type of parasomnias disorder were included in the meta-analysis.

The pooled prevalence of parasomnia disorders ranged between 5.6% in Jordan and 17.4% in Saudi Arabia (Table 2). A significant difference in the prevalence of parasomnia disorders was found between Jordan and Saudi Arabia. Only one study reported prevalence data of parasomnias segregated by sex^71^, and another one^67^ during the COVID-19 pandemic lockdown in Saudi Arabia.

No significant difference was reported in the prevalence of sleep walking (p-value = 0.090) between males and females^71^ (Table 3). The reported factor associated with an increased odds of having nightmares was being a female medical student (*p* value = 0.022)^71^.

#### Sleep-related movement disorders

A total of 14 prevalence measures classified under sleep-related movement disorders were retrieved from 6 studies conducted in Jordan, Egypt, Pakistan, and Saudi Arabia (Table 1).

Following data merging and classification, 9 prevalence measures on any type of sleep-related movement disorder were included in the meta-analysis.

The pooled prevalence of sleep-related movement disorders ranged between 5.9% in Egypt and 30.6% in Saudi Arabia (Table 2). Only one study^67^ reported a prevalence of restless leg syndrome (RLS) during the COVID-19 pandemic lockdown in Saudi Arabia. Significant differences in the pooled prevalence of sleep-related movement disorders were found between Egypt, Jordan, Saudi Arabia, and Pakistan. 5.1% of medical students had a moderate-level of sleep-related movement disorders and 3.0% had a mild-level. No statistically significant difference in sleep-related movement disorders was found between males and females. The reported factor associated with decreased odds of having periodic limb movement disorder/RLS was being a male medical student^71^ (Table 3).

#### Undefined sleep disorder

Only one study reported a prevalence measure of any SD (without type specific SD prevalence), which was 9.5% among a male and female medical student during mixed training periods in Saudi Arabia (Table 1).

#### Heterogeneity

Between-study heterogeneity was relatively high, and differences between prevalence estimates across subgroups were significant between sex groups, academic training periods, and countries for the majority of SDs (Table 2). Meta-regression analyses revealed that prevalence measures retrieved from studies that did not report the SD measurement tool (n = 2) were significantly higher compared with studies that used a validated tool (n = 47). Although not statistically significant, prevalence measures assessed using non-validated tools seem to provide lower prevalence measures as compared to those using validated tools (Table 4). Although not statistically significant, SD prevalence measures based on ‘non-probability sampling’, ‘sampling method not reported’, ‘sample size £ 100’, and ‘response rates^3^ 75%’ were associated with higher SD prevalence measures when compared to ‘probability sampling’, ‘sampling method reported', ‘sample size > 100’, and ‘response rates < 75%’, respectively.

**Table 4:** Univariate meta-regression models for any sleep disorder prevalence in MENA medical students.

Study-level factors	Number of prevalence measures	Total sample size (n = 19,955)	Effect size		Univariable analyses		
			Weighted average prevalence (%)	95% CI (%)	OR	95% CI	p-value†
Sampling							
Probability based	26	10,308	24.3	17.1–32.2	Ref	Ref	Ref
Non-probability based	19	3950	37.0	23.5–51.5	1.74	0.84–3.63	0.139
NR	10	5697	26.7	15.7–38.4	1.18	0.48–2.89	0.724
Sample size							
> 100	45	19,336	27.7	20.8–35.2	Ref	Ref	Ref
≤ 100	10	619	34.7	22.4–48.0	1.50	0.63–3.60	0.361
Tools							
Validated	47	18,274	28.6	22.2–35.4	Ref	Ref	Ref
Non validated	6	991	15.2	7.9–24.2	0.48	0.18–1.32	0.156
NR	2	690	79.3	70.5–86.9	**11.24**	**2.22–56.99**	**0.004**
Response rate							
< 75%	2	135	41.6	20.7–64.1	Ref	Ref	Ref
≥ 75%	6	2628	26.3	16.9–36.9	2.06	0.27–15.56	0.486
NR	47	17,192	28.8	21.7–36.4	1.01	0.35–2.93	0.980

Significant results are highlighted in bold (*p*-value ≤ 0.05). Associations between study-level factors^62^ and the prevalence of combined SDs is reported as odds ratios (ORs) with its corresponding 95% confidence interval.

### Study-level quality assessment

Overall, most included primary studies properly reported the information required to allow quality assessment and were of good methodological quality (low RoB) (Supplementary Table S4). Most of the included studies (86%, 19/22) had a low likelihood of nonresponse bias, collected data directly from the targeted population (91%, 20/22), used an acceptable case definition (82%, 18/22), and used a validated instrument to measure SD (77%, 17/22). All included studies used the same mode of data collection for comparison groups. However, only 32% (7/22) of the included studies used a random-sampling method. A total of 20 studies out of 22 (91%) had a high RoB related to the representativeness of the national and target populations. Most of the included studies (73%, 16/ 22) used an appropriate tool to identify cases with a high risk of SDs. Only one study had a high RoB with ‘including appropriate numerators and denominators for the studied SDs’. Overall, the included studies had good internal validity and moderate external validity that could limit the generalizability of the results.

### Reporting bias and certainty assessment

Our synthesis was likely impacted by the limited number of primary studies retrieved in some specific SDs categories and MENA countries, which may have consequently limited the representativeness of our pooled prevalence estimates. Most of the primary studies had a low risk of non-response bias, however, they had a high risk of selection bias, which could impact their external validity. Additionally, pooled SD prevalence estimates were likely robust because of the good studies’ internal validity (low risk of measurement and analysis biases); however, identified heterogeneity between studies has likely impacted the precision of the pooled prevalence estimates.

**Figure 2:**
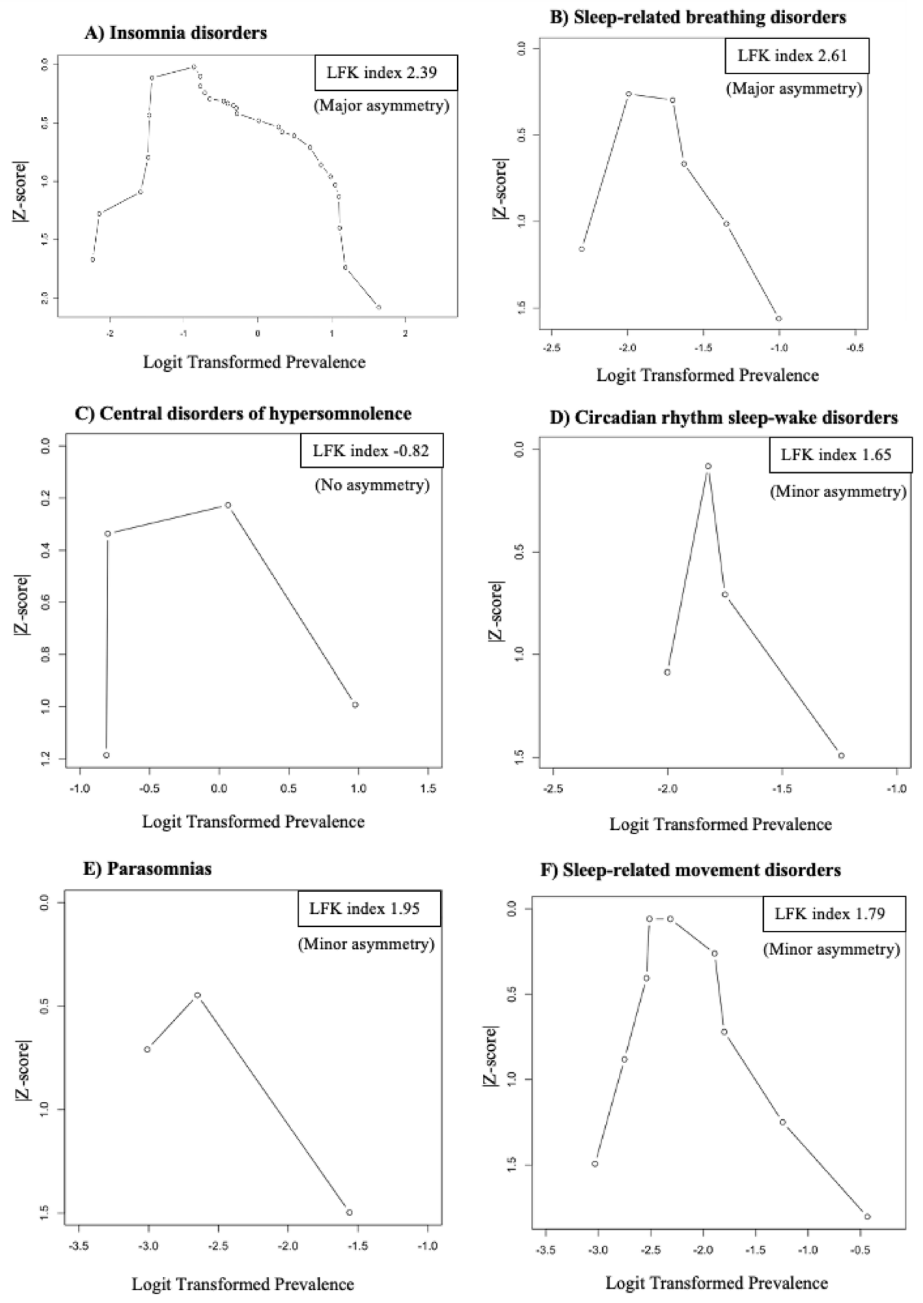
Publication bias assessed via the Doi plot using the prevalence of each sleep disorder as an effect estimate (ES) and the LFK (Luis Furuya-Kanamori) index. In the Doi plot, the dots representing individual prevalence measures extracted from each study on each outcome (sleep disorders).

The visual inspection of the Doi plots indicates some positive asymmetry (with studies spread out towards the right limb), for all SDs except central disorders of hypersomnolence (Fig. 2). The LFK index was consistent with no asymmetry of the Doi plot for central disorders of hypersomnolence and therefore no evidence of a publication bias. The LFK index was consistent with a minor positive asymmetry of the Doi plots (minor publication bias) for (1) circadian rhythm sleep–wake disorders, (2) parasomnias, and (3) sleep-related movement disorders (Fig. 2). LFK index was consistent with a positive major asymmetry of the Doi plot for insomnias disorders and sleep-related breathing disorders. Hence, there may be a major publication bias related to studies with a higher prevalence more likely to be published. The prediction 95% intervals for the prevalence of on insomnias disorders and sleep-related breathing disorders were [6.67%-87.59%] and [5.3%-40.40%], respectively.

Based on the above, the certainty of available evidence was rated as moderate.

## Discussion

The pooled prevalence of SD categories among MENA medical students was the highest for central disorders of hypersomnolence, insomnia, and CRDs. Statistically significant differences in the pooled prevalence were identified between Egypt, Jordan, Morocco, Pakistan, and Saudi Arabia. While insomnia disorders were generally studied in the region, limited data were found for central disorders of hypersomnolence, CRDs, sleep-related breathing disorders, sleep-related movement disorders, and parasomnias disorders preventing conclusions on their extent and positioning. Limited data on the prevalence of SDs among medical students were available globally^77^, and a wide range of SD prevalence measures was reported among university students globally^9,19,25,78^. Additionally, only two studies assessed the impact of the COVID-19 pandemic lockdown on the prevalence insomnias disorders.

Insomnia disorder prevalence among the MENA medical students, ranging between 30.4% and 59.1%, was higher than medical students in China (27.8%)^79^ and lower than medical students in Georgia (70.11%)^80^. Insomnia prevalence in MENA medical students was comparable to the global insomnia prevalence reported among healthcare workers (37–38%)^81,82^. Medical students typically cope with large academic loads and clinical duties that include overnight on-call shifts, which could interfere with their sleep^6,26^. However, medical students may also overreport symptoms compared to non-medical university students or the general population, because of their medical knowledge and their perceived value on health^83^, which could contribute to the higher self-reported SD prevalence.

Globally, a wide range of insomnia prevalence measures have been reported in university students^9,19,36–38,84^ and the adult general population^5,37,38,81,85–89^ preventing comparisons. The differences in the diagnostic criteria used to assess insomnia could explain some of the observed differences in the insomnia prevalence between studies.

The variability in insomnia and other SD prevalence measures in the literature could also be explained by the use of sleep disturbance tools, such as Pittsburgh Sleep Quality Index, for assessing the risk of SDs^9,19,25,35^. Although sleep disturbance tools are good predictors of the risk of SDs^90,91^, only a small proportion of students with sleep complaints will meet SD clinical interview and diagnostic criteria^78^. For instance, 30–40% of the population with sleep disturbances will meet the clinical diagnostic scores for insomnia^35^, as symptoms related to middle-of-night awakenings or daytime impairments are not captured by sleep quality tools and are required to fulfill the criteria for insomnia disorders^92,93^.

Our meta-analysis demonstrated that insomnia disorders and sleep-related breathing disorders were significantly more prevalent in female medical students as compared to male students. Sex-related differences were not identified for other SDs. The synthesis of reported factors associated with SDs suggested that the female sex was associated with the occurrence of insomnia disorders, nightmares, periodic limb movement disorder, and RLS; and the male sex was associated with the occurrence of OSA. The higher vulnerability of women to insomnia^5,94–97^ and RLS^5,98,99^ as compared to men is consistent with previous findings in the general population, suggesting the need to target female students when planning interventions.

Differences in SD prevalence by academic training period or disorder severity level assessed in our meta-analyses could not be established given the limited data; however, included studies suggested that late academic years (clinical and late clinical years) were associated with insomnia and OSA. Medical education in general and the clinical years specifically have been identified as causative factors for poor sleep quality worldwide^6,100^. Our findings highlighted the significant negative impact of insomnia disorders, sleep state misperception, narcolepsy, and CRD disorders on academic performance in medical school. Low academic performance among medical and university students has been correlated with poor sleep quality^20,101^. Additional studies are required to assess the impact of SDs on the academic performance of MENA medical students. Interventions involving sleep-education^24,102^, monitoring cognitive behavior^24,102^, and mindfulness relaxation^24,102^ during clinical training years can help medical students to improve their sleep. Student well-being services can support students in managing disturbed sleep and its consequences. Additionally, incorporating sleep education into the curriculum has been recommended to address medical students’ poor knowledge and misconceptions about sleep practices and disorders^6,103,104^ and prevent misdiagnosis and maltreatment of SDs^105^.

Our synthesis of reported factors associated with SDs suggested that excessive daily internet use was associated with the occurrence of insomnia disorders and OSA. A significant increase in sleep problems and a reduction in sleep duration were found among individuals addicted to the internet^106^, suggesting a potential association with SDs. Additionally, our synthesis suggested that the negative impact of anxiety on the risk of insomnia disorders and OSA depends on the severity level of anxiety. Both insomnia disorders^77,94,107–112^ and OSA^113–115^ are associated with an increased risk of depression and anxiety in adults and adolescents, and it seems that this relationship is bi-directional^116^. As anxiety and depressive symptoms are relatively common in medical students^117,118^, they probably contribute to the increased prevalence of insomnia disorders and OSA in this population. Consequently, interventions designed to prevent or address SDs are also likely to positively impact mental health disorders holistically.

To our knowledge, this is the first SR and meta-analysis focusing on SDs rather than sleep disturbances in MENA medical students. The majority of included studies were of good methodological quality, which reinforces the validity of our findings. A minor impact of the two studies not reporting the tool for measuring SD prevalence on the pooled prevalence is expected given that 85% of the included studies used a validated tool for assessing SDs. There was no evidence for the impact of other study characteristics on the SD prevalence (Table 4). All included studies assessed SDs using self-reported questionnaires, which could be subject to recall bias. Also, all included studies have used screening tools for assessing SDs. Therefore, the proportion of medical students meeting the clinical diagnosis criteria is expected to be lower than the estimated proportion of students with SDs considering screening criteria only^78^. While an objective clinical diagnosis of SDs is generally more reliable, self-reported screening questionnaires are utilized not only in research but also in clinical practice because of their administration efficiency and low cost^119^. Most studies included in this review were comprised of rather small sample sizes or limited generalizability. Studies with larger samples and geographical coverage are required to confirm our results. Our findings may not be generalizable to all MENA countries given the limited number of countries with available data. Although a publication bias related to the available data on insomnias disorders and sleep-related breathing disorders has been detected, we are confident that the prevalence of these SDs are within the prediction interval. Despite these limitations and the existence of heterogeneity, several subgroup analyses of SD prevalence assessed using validated tools were conducted. Therefore, these limitations do not affect the interpretation of our findings.

As most of the included studies were cross-sectional, temporal sequencing of SD development and potential associated factors cannot be established, which limits conclusions on potential causal associations. However, this synthesis can be used to generate hypotheses and support future study design to assess the risk factors and consequences of SDs in medical students.

## Conclusion

SDs with the highest prevalence among medical students in MENA were central disorders of hypersomnolence, insomnia disorders, and CRD disorders. Female sex, latter academic years, anxiety, and excessive internet use were associated with the occurrence of several SDs. SDs negatively impact students’ academic performance. Implementing public health and clinical interventions in medical school settings, particularly targeting high-risk groups (i.e., female students and students in late academic years), should be given serious consideration to help improve students’ overall health, wellbeing, and quality of life.

### Supplementary Information

Supplementary Information.

## Data Availability

The original contributions presented in the study are included in the article and online supplement. Further inquiries can be directed to the corresponding author.

## References

[1] Sateia MJ (2014). International classification of sleep disorders-third edition: highlights and modifications. Chest.

[2] Hypersomnia Foundation. International classification of sleep disorders (ICSD) 2022 [Available from: https://www.hypersomniafoundation.org/glossary/international-classification-of-sleep-disorders/#:~:text=The%20ICSD%2D3%20groups%20sleep,)%20sleep%2Drelated%20movement%20disorders

[3] American Academy of Sleep Medicine (2014). The International Classification of Sleep Disorders – Third Edition (ICSD-3).

[4] American Sleep Association. Sleep disorders – ICD-10 codes and names (2015).

[5] Karna B, Sankari A, Tatikonda G. Sleep Disorder. [Updated 2022 Nov 26]. 2022. In: StatPearls. Treasure Island (FL): Karna B, Sankari A, Tatikonda G. Sleep Disorder. [Updated 2022 Jul 19], in StatPearls [Internet]. Treasure Island (FL) (StatPearls Publishing, 2022). Available from: https://www.ncbi.nlm.nih.gov/books/NBK560720/?report=classic. Available from: https://www.ncbi.nlm.nih.gov/books/NBK560720/?report=classic

[6] Azad MC, Fraser K, Rumana N, Abdullah AF, Shahana N, Hanly PJ (2015). Sleep disturbances among medical students: A global perspective. J. Clin. Sleep Med..

[7] Streatfeild J, Smith J, Mansfield D, Pezzullo L, Hillman D (2021). The social and economic cost of sleep disorders. Sleep.

[8] Carney CE, Moss TG, Lachowski AM, Atwood ME (2014). Understanding mental and physical fatigue complaints in those with depression and insomnia. Behav. Sleep Med..

[9] Jiang X, Zheng XY, Yang J, Ye CP, Chen YY, Zhang ZG (2015). A systematic review of studies on the prevalence of insomnia in university students. Public Health.

[10] Hanin C, Arnulf I, Maranci JB, Lecendreux M, Levinson DF, Cohen D (2021). Narcolepsy and psychosis: A systematic review. Acta Psychiatrica Scand..

[11] Lo JC, Chong PL, Ganesan S, Leong RL, Chee MW (2016). Sleep deprivation increases formation of false memory. J. Sleep Res..

[12] Wang C, Tan J, Miao Y, Zhang Q (2022). Obstructive sleep apnea, prediabetes and progression of type 2 diabetes: A systematic review and meta-analysis. J. Diabetes Investig..

[13] Qie R, Zhang D, Liu L, Ren Y, Zhao Y, Liu D (2020). Obstructive sleep apnea and risk of type 2 diabetes mellitus: A systematic review and dose-response meta-analysis of cohort studies. J. Diabetes.

[14] Zheng Z, Wang C, Li C, Wu Q, Chen X, Chen H (2022). Meta-analysis of relationship of sleep quality and duration with risk of diabetic retinopathy. Front. Endocrinol. (Lausanne).

[15] Partinen M, Putkonen PT, Kaprio J, Koskenvuo M, Hilakivi I (1982). Sleep disorders in relation to coronary heart disease. Acta Med. Scand. Suppl..

[16] Heilbrunn ES, Ssentongo P, Chinchilli VM, Oh J, Ssentongo AE (2021). Sudden death in individuals with obstructive sleep apnoea: A systematic review and meta-analysis. BMJ Open Respir. Res..

[17] Chattu VK, Sakhamuri SM, Kumar R, Spence DW, BaHammam AS, Pandi-Perumal SR (2018). Insufficient sleep syndrome: Is it time to classify it as a major noncommunicable disease?. Sleep Sci..

[18] Liu Y, Wheaton AG, Chapman DP, Cunningham TJ, Lu H, Croft JB (2016). Prevalence of healthy sleep duration among adults-United States, 2014. MMWR Morb. Mortal. Wkly. Rep..

[19] Li L, Wang Y-Y, Wang S-B, Zhang L, Li L, Xu D-D (2018). Prevalence of sleep disturbances in Chinese university students: A comprehensive meta-analysis. J. Sleep Res..

[20] Seoane HA, Moschetto L, Orliacq F, Orliacq J, Serrano E, Cazenave MI (2020). Sleep disruption in medicine students and its relationship with impaired academic performance: A systematic review and meta-analysis. Sleep Med. Rev..

[21] Khaksarian M, Behzadifar M, Behzadifar M, Jahanpanah F, Guglielmi O, Garbarino S (2020). Sleep disturbances rate among medical and allied health professions students in Iran: Implications from a systematic review and meta-analysis of the literature. Int. J. Environ. Res. Public Health.

[22] Rao WW, Li W, Qi H, Hong L, Chen C, Li CY (2020). Sleep quality in medical students: A comprehensive meta-analysis of observational studies. Sleep Breath.

[23] Russell K, Allan S, Beattie L, Bohan J, MacMahon K, Rasmussen S (2019). Sleep problem, suicide and self-harm in university students: A systematic review. Sleep Med. Rev..

[24] Gardani M, Bradford DRR, Russell K, Allan S, Beattie L, Ellis JG (2022). A systematic review and meta-analysis of poor sleep, insomnia symptoms and stress in undergraduate students. Sleep Med. Rev..

[25] Chowdhury AI, Ghosh S, Hasan MF, Khandakar KAS, Azad F (2021). Prevalence of insomnia among university students in South Asian Region: A systematic review of studies. J. Prev. Med. Hyg..

[26] Shad R, Thawani R, Goel A (2015). Burnout and sleep quality: A cross-sectional questionnaire-based study of medical and non-medical students in India. Cureus.

[27] Binjabr MA, Alalawi IS, Alzahrani RA, Albalawi OS, Hamzah RH, Ibrahim YS, et al. The worldwide prevalence of sleep problems among medical students by problem, country, and COVID-19 status: A systematic review, meta-analysis, and meta-regression of 109 studies involving 59427 participants. *Curr. Sleep Med. Rep* 1–19 (2023). 10.1007/s40675-023-00258-5 PMC10238781 37359215

[28] Jannathul F, Emdadul H, Anis L, Auni N, Fatin N (2023). Sleep disruption and its impact on academic performance in medical students: A systematic review. Univers. J. Public Health.

[29] Jahrami H, Dewald-Kaufmann J, Faris MA-I, AlAnsari AMS, Taha M, AlAnsari N (2020). Prevalence of sleep problems among medical students: A systematic review and meta-analysis. J. Public Health.

[30] Jahrami H, Alshomili H, Almannai N, Althani N, Aloffi A, Algahtani H (2019). Predictors of excessive daytime sleepiness in medical students: a meta-regression. Clocks Sleep.

[31] Al-Ajlouni YA, Al Ta'ani O, Shamaileh G, Mushasha R, Makarem N, Duncan DT (2022). Effects of the COVID-19 pandemic on sleep health among Middle Eastern and North African (MENA) populations: A systematic review of the literature. BMJ Open.

[32] Salehinejad MA, Azarkolah A, Ghanavati E, Nitsche MA (2022). Circadian disturbances, sleep difficulties and the COVID-19 pandemic. Sleep Med..

[33] Chandler L, Patel C, Lovecka L, Gardani M, Walasek L, Ellis J (2022). Improving university students’ mental health using multi-component and single-component sleep interventions: A systematic review and meta-analysis. Sleep Med..

[34] Mulyadi M, Tonapa SI, Luneto S, Lin WT, Lee BO (2021). Prevalence of mental health problems and sleep disturbances in nursing students during the COVID-19 pandemic: A systematic review and meta-analysis. Nurse Educ. Pract..

[35] Jahrami HA, Alhaj OA, Humood AM, Alenezi AF, Fekih-Romdhane F, AlRasheed MM (2022). Sleep disturbances during the COVID-19 pandemic: A systematic review, meta-analysis, and meta-regression. Sleep Med. Rev..

[36] Deng J, Zhou F, Hou W, Silver Z, Wong CY, Chang O (2021). The prevalence of depressive symptoms, anxiety symptoms and sleep disturbance in higher education students during the COVID-19 pandemic: a systematic review and meta-analysis. Psychiatry Res..

[37] Zhang SX, Chen RZ, Xu W, Yin A, Dong RK, Chen BZ (2022). A Systematic review and meta-analysis of symptoms of anxiety, depression, and insomnia in Spain in the COVID-19 crisis. Int. J. Environ. Res. Public Health.

[38] Zou Q, Tang Y, Jiang C, Lin P, Tian J, Sun S (2022). Prevalence of anxiety, depressive and insomnia symptoms among the different groups of people during COVID-19 pandemic: An overview of systematic reviews and meta-analyses. Front. Psychol..

[39] Duvivier RJ, Boulet JR, Opalek A, van Zanten M, Norcini J (2014). Overview of the world's medical schools: An update. Med. Educ..

[40] Moher D, Liberati A, Tetzlaff J, Altman DG, PRISMA Group (2009). Preferred reporting items for systematic reviews and meta-analyses: The PRISMA statement. J. Clin. Epidemiol..

[41] Rethlefsen ML, Kirtley S, Waffenschmidt S, Ayala AP, Moher D, Page MJ (2021). PRISMA-S: An extension to the PRISMA statement for reporting literature searches in systematic reviews. Syst. Rev..

[42] Stroup DF, Berlin JA, Morton SC, Olkin I, Williamson GD, Rennie D (2000). Meta-analysis of observational studies in epidemiology: A proposal for reporting. Meta-analysis of observational studies in epidemiology (MOOSE) group. JAMA.

[43] Chaabna K, Cheema S, Abraham A, Alrouh H, Mamtani R, Sheikh JI (2018). Gray literature in systematic reviews on population health in the Middle East and North Africa: Protocol of an overview of systematic reviews and evidence mapping. Syst. Rev..

[44] Chaabane S, Chaabna K, Abraham A, Mamtani R, Cheema S (2020). Physical activity and sedentary behaviour in the Middle East and North Africa: An overview of systematic reviews and meta-analysis. Sci. Rep..

[45] Chaabane S, Chaabna K, Doraiswamy S, Mamtani R, Cheema S (2021). Barriers and facilitators associated with physical activity in the Middle East and North Africa region: A systematic overview. Int. J. Environ. Res. Public Health.

[46] Doraiswamy S, Jithesh A, Chaabane S, Abraham A, Chaabna K, Cheema S (2020). Perinatal mental illness in the Middle East and North Africa region-a systematic overview. Int. J. Environ. Res. Public Health.

[47] Chaabna K, Cheema S, Abraham A, Maisonneuve P, Lowenfels AB, Mamtani R (2021). The state of population health research performance in the Middle East and North Africa: A meta-research study. Syst. Rev..

[48] Chaabna K, Cheema S, Abraham A, Mamtani R (2020). Strengthening literature search strategies for systematic reviews reporting population health in the Middle East and North Africa: A meta-research study. J. Evid. Based Med..

[49] Chaabna K, Cheema S, Abraham A, Alrouh H, Lowenfels AB, Maisonneuve P (2018). Systematic overview of hepatitis C infection in the Middle East and North Africa. World J. Gastroenterol..

[50] Chaabane S, Doraiswamy S, Chaabna K, Mamtani R, Cheema S (2021). The impact of COVID-19 school closure on child and adolescent health: A rapid systematic review. Children (Basel).

[51] Chaabane S, Chaabna K, Bhagat S, Abraham A, Doraiswamy S, Mamtani R (2021). Perceived stress, stressors, and coping strategies among nursing students in the Middle East and North Africa: An overview of systematic reviews. Syst. Rev..

[52] Singh SCS, Matchar DB, Bass EB, Chang SM, Matchar DB, Smetana GW (2012). Grading a body of evidence on diagnostic tests. Methods Guide for Medical Test Reviews [Internet].

[53] Hoy D, Brooks P, Woolf A, Blyth F, March L, Bain C (2012). Assessing risk of bias in prevalence studies: Modification of an existing tool and evidence of interrater agreement. J. Clin. Epidemiol..

[54] Dekkers OM, Vandenbroucke JP, Cevallos M, Renehan AG, Altman DG, Egger M (2019). COSMOS-E: Guidance on conducting systematic reviews and meta-analyses of observational studies of etiology. PLOS Med..

[55] Terracciano L, Brozek J, Compalati E, Schünemann H (2010). GRADE system: New paradigm. Curr. Opin. Allergy Clin. Immunol..

[56] Michael B, Larry VH, Julian PTH, Hannah RR (2009). Random-Effects Model. Introduction to Meta-Analysis.

[57] Freeman MF, Tukey JW (1950). Transformations related to the angular and the square root. Ann. Math. Stat..

[58] Turner RM, Bird SM, Higgins JP (2013). The impact of study size on meta-analyses: Examination of underpowered studies in Cochrane reviews. PLoS One..

[59] Higgins JPT, Thompson SG, Deeks JJ, Altman DG (2003). Measuring inconsistency in meta-analyses. BMJ.

[60] Schwarzer G. General package for meta-analysis.: CRAN; 2018. Available from: https://github.com/guido-s/meta, http://meta-analysis-with-r.org

[61] Cochrane Handbook for Systematic Reviews of Interventions. Version 6.22021.

[62] Higgins, J. P. T., Green, S. (eds) 9 analysing data and undertaking meta-analyses > 9.6 investigating heterogeneity > 9.6.4 meta-regression. 2011, in *Cochrane Handbook for Systematic Reviews of Interventions Version 510 [updated March 2011] [Internet]. The Cochrane Collaboration*. Available from: Higgins JPT, Green S (editors). Cochrane Handbook for Systematic Reviews of Interventions Version 5.1.0 [updated March 2011]. The Cochrane Collaboration, 2011. Available from www.handbook.cochrane.org.

[63] Furuya-Kanamori L, Barendregt JJ, Doi SAR (2018). A new improved graphical and quantitative method for detecting bias in meta-analysis. JBI Evid. Implement..

[64] Hunter JP, Saratzis A, Sutton AJ, Boucher RH, Sayers RD, Bown MJ (2014). In meta-analyses of proportion studies, funnel plots were found to be an inaccurate method of assessing publication bias. J. Clin. Epidemiol..

[65] Spineli LM, Pandis N (2020). Prediction interval in random-effects meta-analysis. Am. J. Orthod. Dentofac. Orthop..

[66] Shamim MA (2023). Real-life implications of prevalence meta-analyses? Doi plots and prediction intervals are the answer. Lancet Microbe.

[67] Abdelmoaty Goweda R, Hassan-Hussein A, Ali Alqahtani M, Janaini MM, Alzahrani AH, Sindy BM (2021). Prevalence of sleep disorders among medical students of Umm Al-Qura University, Makkah, Kingdom of Saudi Arabia. J. Public Health Res..

[68] Alrashed FA (2021). Prevalence of insomnia and related psychological factors with coping strategies among medical students in clinical years during the COVID-19 pandemic. Saudi J. Bio. Sci..

[69] Essangri H, Sabir M, Benkabbou A, Majbar MA, Amrani L, Ghannam A (2021). Predictive factors for impaired mental health among medical students during the early stage of the COVID-19 pandemic in Morocco. Am. J. Trop. Med. Hyg..

[70] Zainab S, Soomro RA, Khoso A, Qazi NA, Siddiqui S, Zainab S (2020). Frequency and predictors of sleep disorders in undergraduate medical students. J Liaquat Univ. Med. Health Sci..

[71] Yassin A, Al-Mistarehi AH, Beni Yonis O, Aleshawi AJ, Momany SM, Khassawneh BY (2020). Prevalence of sleep disorders among medical students and their association with poor academic performance: A cross-sectional study. Ann. Med. Surg. (Lond.).

[72] Al-mistarehi A, Ibnian A, Shaqadan S, Khassawneh B, Al-mistarehi AW, Ibnian AM (2019). The impact of sleep disorders on academic performance among medical students. Am. J. Respir. Crit. Care Med..

[73] Alshaaer, N. E. F., Marashli, E., Mahgoub, M., & Alashqae, A. A. The prevalence of Insomnia in medical students: Impact of academic performance (2012).

[74] Sleep Foundation. Paradoxical Insomnia: The misperception of your sleep state 2022 Available from: https://www.sleepfoundation.org/insomnia/paradoxical-insomnia

[75] Mohamed E, Abdulrahim S, Sami W, Althaqib A, Alzuwayyid A, Almutiri K (2020). Insomnia and related anxiety among medical students. J. Res. Med. Dent. Sci..

[76] Burhan, N. M. Prevalence of sleep disorders among medical students at King Abdulaziz University: A cross-sectional study (2019).

[77] Mokarrar M, Afsharmanesh A, Afshari M, Mohammadi F (2017). Prevalence of sleep disorder among medical students in an Eastern University in Iran. Iran. J. Health Sci..

[78] Thomas, S. J. A survey of sleep disorders in college students: A study of prevalence and outcomes: University of Alabama Libraries (2014).

[79] Zhang M, Qin L, Zhang D, Tao M, Han K, Chi C (2023). Prevalence and factors associated with insomnia among medical students in China during the COVID-19 pandemic: Characterization and associated factors. BMC Psychiatry.

[80] Solanki S, Venkiteswaran A, Saravanabawan P (2023). Prevalence of insomnia and factors influencing its incidence in students of tbilisi state medical university: A cross-sectional study. Cureus.

[81] Cénat JM, Blais-Rochette C, Kokou-Kpolou CK, Noorishad PG, Mukunzi JN, McIntee SE (2021). Prevalence of symptoms of depression, anxiety, insomnia, posttraumatic stress disorder, and psychological distress among populations affected by the COVID-19 pandemic: A systematic review and meta-analysis. Psychiatry Res..

[82] Pappa S, Ntella V, Giannakas T, Giannakoulis VG, Papoutsi E, Katsaounou P (2020). Prevalence of depression, anxiety, and insomnia among healthcare workers during the COVID-19 pandemic: A systematic review and meta-analysis. Brain Behav. Immun..

[83] Moss-Morris R, Petrie KJ (2001). Redefining medical students’ disease to reduce morbidity. Med. Educ..

[84] Pavlinac Dodig I, Lusic Kalcina L, Demirovic S, Pecotic R, Valic M, Dogas Z (2023). Sleep and lifestyle habits of medical and non-medical students during the COVID-19 lockdown. Behav. Sci..

[85] Roth T, Coulouvrat C, Hajak G, Lakoma MD, Sampson NA, Shahly V (2010). Prevalence and perceived health associated with insomnia based on DSM-IV-TR; International statistical classification of diseases and related health problems, tenth revision; and research diagnostic criteria/international classification of sleep disorders, second edition criteria: Results from the America Insomnia survey. Biol. Psychiatry.

[86] Buysse DJ (2013). Insomnia. JAMA..

[87] Morin CM, LeBlanc M, Bélanger L, Ivers H, Mérette C, Savard J (2011). Prevalence of insomnia and its treatment in Canada. Can. J. Psychiatry.

[88] Ohayon MM, Smirne S (2002). Prevalence and consequences of insomnia disorders in the general population of Italy. Sleep Med..

[89] Léger D, Partinen M, Hirshkowitz M, Chokroverty S, Hedner J (2010). EQUINOX (Evaluation of daytime QUality impairment by nocturnal awakenings in outpatient’s eXperience) survey investigators. Characteristics of insomnia in a primary care setting: EQUINOX survey of 5293 insomniacs from 10 countries. Sleep Med..

[90] Buysse DJ, Hall ML, Strollo PJ, Kamarck TW, Owens J, Lee L (2008). Relationships between the pittsburgh sleep quality index (PSQI), epworth sleepiness scale (ESS), and clinical/polysomnographic measures in a community sample. J. Clin. Sleep Med..

[91] Backhaus J, Junghanns K, Broocks A, Riemann D, Hohagen F (2002). Test-retest reliability and validity of the pittsburgh sleep quality index in primary insomnia. J. Psychosom. Res..

[92] Edinger JD, Bonnet MH, Bootzin RR, Doghramji K, Dorsey CM, Espie CA (2004). American academy of sleep medicine work group. Derivation of research diagnostic criteria for insomnia: Report of an American academy of sleep medicine work group. Sleep.

[93] Lichstein KL, Durrence HH, Taylor DJ, Bush AJ, Riedel BW (2003). Quantitative criteria for insomnia. Behav. Res. Ther..

[94] Sivertsen B, Krokstad S, Øverland S, Mykletun A (2009). The epidemiology of insomnia: associations with physical and mental health. The HUNT-2 study. J. Psychosom. Res..

[95] Phillips BA, Collop NA, Drake C, Consens F, Vgontzas AN, Weaver TE (2008). Sleep disorders and medical conditions in women. J. Womens Health (Larchmt)..

[96] Tamanna S, Geraci SA (2013). Major sleep disorders among women: (women’s health series). South Med. J..

[97] Rodriguez JC, Dzierzewski JM, Alessi CA (2015). Sleep problems in the elderly. Med. Clin. N. Am..

[98] Allen RP, Walters AS, Montplaisir J, Hening W, Myers A, Bell TJ (2005). Restless legs syndrome prevalence and impact: REST general population study. Arch Intern. Med..

[99] Alsafadi S, Abaalkhail B, Wali SO, Aljammali K, Alotaiby B, Zakaria I (2018). Risk factors of primary and secondary restless legs syndrome among a middle-aged population in Saudi Arabia: A community-based study. Ann. Thorac. Med..

[100] Mahajan AS (2010). Stress in medical education: A global issue or much ado about nothing specific?. South-East Asian J. Med. Educ..

[101] Suardiaz-Muro M, Morante-Ruiz M, Ortega-Moreno M, Ruiz MA, Martín-Plasencia P, Vela-Bueno A (2020). Sleep and academic performance in university students: A systematic review. Rev. Neurol..

[102] Friedrich A, Schlarb AA (2018). Let’s talk about sleep: A systematic review of psychological interventions to improve sleep in college students. J. Sleep Res..

[103] Jain A, Wadhwa R, Kundu K, Nebhinani N, Gupta R (2022). Assessment of knowledge about RLS among medical teachers and undergraduate students using newly developed questionnaire: K-RLS. Sleep Vigil..

[104] Ozoh OB, Iwuala SO, Desalu OO, Ojo OO, Okubadejo NU (2015). An assessment of the knowledge and attitudes of graduating medical students in lagos, nigeria, regarding obstructive sleep apnea. Ann. Am. Thorac. Soc..

[105] Rosen RC, Rosekind M, Rosevear C, Cole WE, Dement WC (1993). Physician education in sleep and sleep disorders: A national survey of U.S. medical schools. Sleep.

[106] Alimoradi Z, Lin CY, Broström A, Bülow PH, Bajalan Z, Griffiths MD (2019). Internet addiction and sleep problems: A systematic review and meta-analysis. Sleep Med. Rev..

[107] Khurshid KA (2018). Comorbid Insomnia and psychiatric disorders: An update. Innov. Clin. Neurosci..

[108] Pigeon WR, Bishop TM, Krueger KM (2017). Insomnia as a precipitating factor in new onset mental illness: A systematic review of recent findings. Curr. Psychiatry Rep..

[109] Sarsour K, Morin CM, Foley K, Kalsekar A, Walsh JK (2010). Association of insomnia severity and comorbid medical and psychiatric disorders in a health plan-based sample: Insomnia severity and comorbidities. Sleep Med..

[110] Ohayon MM, Roth T (2003). Place of chronic insomnia in the course of depressive and anxiety disorders. J. Psychiatr. Res..

[111] Roane BM, Taylor DJ (2008). Adolescent insomnia as a risk factor for early adult depression and substance abuse. Sleep.

[112] Neckelmann D, Mykletun A, Dahl AA (2007). Chronic insomnia as a risk factor for developing anxiety and depression. Sleep..

[113] Björnsdóttir E, Benediktsdóttir B, Pack AI, Arnardottir ES, Kuna ST, Gíslason T (2016). The prevalence of depression among untreated obstructive sleep apnea patients using a standardized psychiatric interview. J. Clin. Sleep Med..

[114] McCall WV, Harding D, O’Donovan C (2006). Correlates of depressive symptoms in patients with obstructive sleep apnea. J. Clin. Sleep Med..

[115] Macey PM, Woo MA, Kumar R, Cross RL, Harper RM (2010). Relationship between obstructive sleep apnea severity and sleep, depression and anxiety symptoms in newly-diagnosed patients. PLoS One.

[116] Jansson-Fröjmark M, Lindblom K (2008). A bidirectional relationship between anxiety and depression, and insomnia? A prospective study in the general population. J. Psychosom. Res..

[117] Zeng W, Chen R, Wang X, Zhang Q, Deng W (2019). Prevalence of mental health problems among medical students in China: A meta-analysis. Med. (Baltimore).

[118] Mirza AA, Baig M, Beyari GM, Halawani MA, Mirza AA (2021). Depression and anxiety among medical students: A brief overview. Adv. Med. Educ. Pract..

[119] Luyster FS, Choi J, Yeh C-H, Imes CC, Johansson AEE, Chasens ER (2015). Screening and evaluation tools for sleep disorders in older adults. Appl. Nurs. Res..

[120] Pervez S, Kumar H, Bai S, Kumar R, Parkash O (2021). Prevalence of Insomnia among medical students. Pak. J. Med. Health Sci..

[121] Khan K, Waqas M, Sarwar R, Ahmad S, Faizan M, Khan K (2019). Effects of insomnia on daily performance of medical students: A cross sectional study conducted in university of Lahore, Pakistan. Rawal Med. J..

[122] Khurshid R, Majeed S, Amer L, Rana S, Ikram S, Upal S (2021). Attitudes and reactions of medical students to the dissection room. Pak. J. Med. Health Sci..

[123] Ali A, Mehmood S, Farooq L, Arif H, Korai N, Khan M (2019). Influence of excessive mobile phone use on anxiety and academic performance among medical college students. J. Pharm. Res. Int..

[124] Shakeel HA, Maqsood H, Ishaq A, Ali B, Hussain H, Khan AR (2019). Insomnia among medical students: A crosssectional study. Int. J. Res. Med. Sci..

[125] Ram D (2016). Frequency of insomnia amongst medical students and its correlation with demographic variables. J. Pak. Pyschiatr. Soc..

[126] Alfadeel M, Alqahtani N, Alhudaib M, Almudhee S, Alghamdi A, Jadou N (2020). The prevalence of insomnia among female medical students of almaarefa colleges in Riyadh city -Kingdom of Saudi Arabia 2015–2016. Indo Am. J. Pharm. Sci..

[127] Mansour T, Yousef M, Mansour TMA, Yousef M (2016). Nightmares among young medical students. Biomed. Res.-India.

[128] Al-Zahrani, J. M., Aldossari, K. K., Abdulmajeed, I., Al-Ghamdi, S. H., Al-Shamrani, A. M., & Al-Qahtani, N. S., et al. Daytime sleepiness and academic performance among arab medical students. *Am. J. Respir. Crit. Care Med.* **193** (2016).

[129] Goweda R, Idris K, Bakhsh A, Mufti H, Gadhi M, Alrashed A (2020). Prevalence and associated risk factor of low back pain among medical student of Umm Al-Qura University, Makkah, Saudi Arabia: Cross-sectional study. Med. Sci..

[130] Alqudah M, Balousha SAM, Al-Shboul O, Al-Dwairi A, Alfaqih MA, Alzoubi KH (2019). Insomnia among medical and paramedical students in Jordan: Impact on academic performance. BioMed Res. Int..

[131] Ishaq M, Riaz SU, Iqbal N, Siddiqui S, Moin A, Sajjad S (2020). Prevalence of restless legs syndrome among medical students of karachi: An experience from a developing country. Sleep Disord..

[132] Shalash AS, Elrassas HH, Monzem MM, Salem HH, Abdel Moneim A, Moustafa RR (2015). Restless legs syndrome in Egyptian medical students using a validated Arabic version of the restless legs syndrome rating scale. Sleep Med..

[133] Almansour A, AlJammaz F, Ahmeda A, Alfawaz M, Abdulsalam K, AlSheikh A (2020). the prevalence of sleep deprivation and its influence on student’' life attending medical School at King Saud University. Int. J. Pharm. Phytopharmacol. Res..

