# Supplementary material for: Sleep disorders and associated factors among medical students in the Middle East and North Africa: a systematic review and meta-analysis
Source: Sci Rep. 2024 Feb 26;14:4656. doi: 10.1038/s41598-024-53818-2 (PMC10897338; doi:10.1038/s41598-024-53818-2)
Supplement: Supplementary file 1 — Supplementary Information. [file 41598_2024_53818_MOESM1_ESM.docx]

Supplementary Material

Sleep Disorders and Associated Factors Among Medical Students in the Middle East and North Africa: a Systematic Review and Meta-analysis

Sonia Chaabane, PhD^1*^, Karima Chaabna, PhD^1^, Salina Khawaja, MSc^1^, Jasmine Aboughanem, MSW^1^, Dhruv Mittal, MBBS candidate^2^, Ravinder Mamtani, MD, MSc^1^, Sohaila Cheema, MBBS, MPH^1^

^1^Institute for Population Health, Weill Cornell Medicine – Qatar, Education City, Qatar foundation, P.O.Box. 24144, Qatar

^2^Intern, Institute for Population Health, Weill Cornell Medicine – Qatar, Education City, Qatar foundation, P.O.Box. 24144, Qatar

# Supplementary Data, Figures and Tables

# Supplementary Table S1: PRISMA checklist 2020

# Supplementary Table S2: PRISMA checklist for search strategy

# Supplementary Box S1: Search strategy

# Supplementary Table S3: ICSD-3 and specific diagnostic categories

# Supplementary Box S2: List of excluded studies

# Supplementary Table S4: Quality assessment of the included studies

**Table S1: PRISMA checklist 2020**

| **Section and Topic** | **Item #** | **Checklist item** | **Location where item is reported** |
| --- | --- | --- | --- |
| **TITLE** | | |  |
| Title | 1 | Identify the report as a systematic review. | Page 1 |
| **ABSTRACT** | | |  |
| Abstract | 2 | See the PRISMA 2020 for Abstracts checklist. | NA |
| **INTRODUCTION** | | |  |
| Rationale | 3 | Describe the rationale for the review in the context of existing knowledge. | Page 6 |
| Objectives | 4 | Provide an explicit statement of the objective(s) or question(s) the review addresses. | Page 6 |
| **METHODS** | | |  |
| Eligibility criteria | 5 | Specify the inclusion and exclusion criteria for the review and how studies were grouped for the syntheses. | Page 5-11 |
| Information sources | 6 | Specify all databases, registers, websites, organisations, reference lists and other sources searched or consulted to identify studies. Specify the date when each source was last searched or consulted. | Page 5-11 |
| Search strategy | 7 | Present the full search strategies for all databases, registers and websites, including any filters and limits used. | Page 5-11 7  Box S1 |
| Selection process | 8 | Specify the methods used to decide whether a study met the inclusion criteria of the review, including how many reviewers screened each record and each report retrieved, whether they worked independently, and if applicable, details of automation tools used in the process. | Page 5-11 |
| Data collection process | 9 | Specify the methods used to collect data from reports, including how many reviewers collected data from each report, whether they worked independently, any processes for obtaining or confirming data from study investigators, and if applicable, details of automation tools used in the process. | Page 5-11 |
| Data items | 10a | List and define all outcomes for which data were sought. Specify whether all results that were compatible with each outcome domain in each study were sought (e.g. for all measures, time points, analyses), and if not, the methods used to decide which results to collect. | Page 5-11  Table 1 |
|  | 10b | List and define all other variables for which data were sought (e.g. participant and intervention characteristics, funding sources). Describe any assumptions made about any missing or unclear information. | Page 5-11 |
| Study risk of bias assessment | 11 | Specify the methods used to assess risk of bias in the included studies, including details of the tool(s) used, how many reviewers assessed each study and whether they worked independently, and if applicable, details of automation tools used in the process. | Pages 5-11 |
| Effect measures | 12 | Specify for each outcome the effect measure(s) (e.g. risk ratio, mean difference) used in the synthesis or presentation of results. | Page 5-11 |
| Synthesis methods | 13a | Describe the processes used to decide which studies were eligible for each synthesis (e.g. tabulating the study intervention characteristics and comparing against the planned groups for each synthesis (item #5)). | Page 5-11 |
|  | 13b | Describe any methods required to prepare the data for presentation or synthesis, such as handling of missing summary statistics, or data conversions. | Page 5-11 |
|  | 13c | Describe any methods used to tabulate or visually display results of individual studies and syntheses. | Page 5-11 |
|  | 13d | Describe any methods used to synthesize results and provide a rationale for the choice(s). If meta-analysis was performed, describe the model(s), method(s) to identify the presence and extent of statistical heterogeneity, and software package(s) used. | Pages 5-11 |
|  | 13e | Describe any methods used to explore possible causes of heterogeneity among study results (e.g. subgroup analysis, meta-regression). | Page 5-11 |
|  | 13f | Describe any sensitivity analyses conducted to assess robustness of the synthesized results. | Pages 5-11 |
| Reporting bias assessment | 14 | Describe any methods used to assess risk of bias due to missing results in a synthesis (arising from reporting biases). | Page 5-11 |
| Certainty assessment | 15 | Describe any methods used to assess certainty (or confidence) in the body of evidence for an outcome. | Pages 5-11 |
| **RESULTS** | | |  |
| Study selection | 16a | Describe the results of the search and selection process, from the number of records identified in the search to the number of studies included in the review, ideally using a flow diagram. | Page 12-20  Figure 1 |
|  | 16b | Cite studies that might appear to meet the inclusion criteria, but which were excluded, and explain why they were excluded. | Page 12-20  Figure 1  Box S2 |
| Study characteristics | 17 | Cite each included study and present its characteristics. | Table 1 |
| Risk of bias in studies | 18 | Present assessments of risk of bias for each included study. | Table S4 |
| Results of individual studies | 19 | For all outcomes, present, for each study: (a) summary statistics for each group (where appropriate) and (b) an effect estimate and its precision (e.g. confidence/credible interval), ideally using structured tables or plots. | Table 1  Pages 12-20 |
| Results of syntheses | 20a | For each synthesis, briefly summarise the characteristics and risk of bias among contributing studies. | Pages 12-20  Tables 1,2,3 |
|  | 20b | Present results of all statistical syntheses conducted. If meta-analysis was done, present for each the summary estimate and its precision (e.g. confidence/credible interval) and measures of statistical heterogeneity. If comparing groups, describe the direction of the effect. | Pages 12-20  Table 2 |
|  | 20c | Present results of all investigations of possible causes of heterogeneity among study results. | Pages 12-20  Table 4 |
|  | 20d | Present results of all sensitivity analyses conducted to assess the robustness of the synthesized results. | Pages 12-20  Tables 2,4 |
| Reporting biases | 21 | Present assessments of risk of bias due to missing results (arising from reporting biases) for each synthesis assessed. | Page 19-20  Figure 2 |
| Certainty of evidence | 22 | Present assessments of certainty (or confidence) in the body of evidence for each outcome assessed. | Pages 19-20 |
| **DISCUSSION** | | |  |
| Discussion | 23a | Provide a general interpretation of the results in the context of other evidence. | Pages 21-25 |
|  | 23b | Discuss any limitations of the evidence included in the review. | Pages 21-25 |
|  | 23c | Discuss any limitations of the review processes used. | Pages 21-25 |
|  | 23d | Discuss implications of the results for practice, policy, and future research. | Pages 21-25 |
| **OTHER INFORMATION** | | |  |
| Registration and protocol | 24a | Provide registration information for the review, including register name and registration number, or state that the review was not registered. | Page 5 |
|  | 24b | Indicate where the review protocol can be accessed, or state that a protocol was not prepared. | Page 5 |
|  | 24c | Describe and explain any amendments to information provided at registration or in the protocol. | NA |
| Support | 25 | Describe sources of financial or non-financial support for the review, and the role of the funders or sponsors in the review. | NA |
| Competing interests | 26 | Declare any competing interests of review authors. | Page 26 |
| Availability of data, code and other materials | 27 | Report which of the following are publicly available and where they can be found: template data collection forms; data extracted from included studies; data used for all analyses; analytic code; any other materials used in the review. | Page 26 |

*From:*  Page MJ, McKenzie JE, Bossuyt PM, Boutron I, Hoffmann TC, Mulrow CD, et al. The PRISMA 2020 statement: an updated guideline for reporting systematic reviews. BMJ 2021;372:n71. doi: 10.1136/bmj.n71

For more information, visit: <http://www.prisma-statement.org/>

**Table S2: PRISMA checklist for search strategy**

| **Section/topic** | **#** | **Checklist item** | **Location(s) Reported** |
| --- | --- | --- | --- |
| **INFORMATION SOURCES AND METHODS** | | | |
| Database name | 1 | Name each individual database searched, stating the platform for each. | Page 5 |
| Multi-database searching | 2 | If databases were searched simultaneously on a single platform, state the name of the platform, listing all of the databases searched. | Page 5  Box S1 |
| Study registries | 3 | List any study registries searched. | N/A |
| Online resources and browsing | 4 | Describe any online or print source purposefully searched or browsed (e.g., tables of contents, print conference proceedings, web sites), and how this was done. | Page 5  Box S1 |
| Citation searching | 5 | Indicate whether cited references or citing references were examined, and describe any methods used for locating cited/citing references (e.g., browsing reference lists, using a citation index, setting up email alerts for references citing included studies). | Page5  Box S1 |
| Contacts | 6 | Indicate whether additional studies or data were sought by contacting authors, experts, manufacturers, or others. | N/A |
| Other methods | 7 | Describe any additional information sources or search methods used. | Page 5  Box S1 |
| **SEARCH STRATEGIES** | | | |
| Full search strategies | 8 | Include the search strategies for each database and information source, copied and pasted exactly as run. | Method |
| Limits and restrictions | 9 | Specify that no limits were used, or describe any limits or restrictions applied to a search (e.g., date or time period, language, study design) and provide justification for their use. | Page 5  Box S1 |
| Search filters | 10 | Indicate whether published search filters were used (as originally designed or modified), and if so, cite the filter(s) used. | Page 5  Box S1 |
| Prior work | 11 | Indicate when search strategies from other literature reviews were adapted or reused for a substantive part or all of the search, citing the previous review(s). | N/A |
| Updates | 12 | Report the methods used to update the search(es) (e.g., rerunning searches, email alerts). | N/A |
| Dates of searches | 13 | For each search strategy, provide the date when the last search occurred. | Box S1 |
| **PEER REVIEW** | | | |
| Peer review | 14 | Describe any search peer review process. | Page 5  Box S1 |
| **MANAGING RECORDS** | | | |
| Total Records | 15 | Document the total number of records identified from each database and other information sources. | Page 5  Box S1 |
| Deduplication | 16 | Describe the processes and any software used to deduplicate records from multiple database searches and other information sources. | Page 5  Box S1 |
|  |  |  |  |
| PRISMA-S: An Extension to the PRISMA Statement for Reporting Literature Searches in Systematic Reviews | | |  |
| Rethlefsen ML, Kirtley S, Waffenschmidt S, Ayala AP, Moher D, Page MJ, Koffel JB, PRISMA-S Group. | | |  |
| Last updated February 27, 2020. | |  |  |

**Box S1: Search strategy**

**Pubmed**:

Filter: Inception to February 15^th^, 2022

Total reports screened: 1037

("sleep"[MeSH Terms] OR "sleep wake disorders"[MeSH Terms] OR "sleep*"[Text Word] OR insomnia [Text Word] OR somnolence [Text Word]) AND ("students, medical"[MeSH Terms] OR ("medical"[Text Word] AND "students"[Text Word]) OR "medical student*"[Text Word] OR ("medical"[Text Word] AND "student"[Text Word])

**Web of Science**:

Filter: Inception to February 15^th^, 2022

Total reports screened: 1009

(sleep* OR insomnia OR somnolence) AND (“medical student*”)

**Advanced Google Scholar:**

Filter: Inception to June 25^th^, 2022

Total reports screened: 200

Two searches were conducted as there was a limit on the number of words to include in search filters. The MENA countries were split in 2 searches as shown below:

Search 1:

Filter “with all of the words”: Medical AND students AND sleep AND disorders.

AND

Filter “with at least one of the words”: MENA OR Middle OR East OR North OR Africa OR Algeria OR Bahrain OR Djibouti OR Egypt OR Iraq OR Jordan OR Kuwait OR Lebanon OR Libya OR Morocco OR Oman.

Search 2:

Filter “with all of the words”: Medical AND students AND sleep AND disorders.

AND

Filter “with at least one of the words: Palestine OR Qatar OR Saudi OR Arabia OR KSA OR Sudan OR Syria OR Tunisia OR United OR Arab OR Emirates OR UAE OR Yemen.

**Table S3: ICSD-3 major and specific diagnostic categories of sleep disorders** [1]

| **Category 1: Insomnia** |
| --- |
| Chronic insomnia disorder |
| Short-term insomnia disorder |
| Other insomnia disorder |
| **Category 2: Sleep-Related Breathing Disorders** |
| Obstructive sleep apnea disorders |
| Obstructive sleep apnea, adult |
| Obstructive sleep apnea, pediatric |
| **Central sleep apnea syndromes** |
| Central sleep apnea with Cheyne-Stokes breathing |
| Central sleep apnea due to a medical disorder without Cheyne-Stokes breathing |
| Central sleep apnea due to high altitude periodic breathing |
| Central sleep apnea due to a medication or substance |
| Primary central sleep apnea |
| Primary central sleep apnea of infancy |
| Primary central sleep apnea of prematurity |
| Treatment-emergent central sleep apnea |
| **Sleep-related hypoventilation disorders** |
| Obesity hypoventilation syndrome |
| Congenital central alveolar hypoventilation syndrome |
| Late-onset central hypoventilation with hypothalamic dysfunction |
| Idiopathic central alveolar hypoventilation |
| Sleep-related hypoventilation due to a medication or substance |
| **Sleep-related hypoventilation due to a medical disorder** |
| Sleep-related hypoxemia disorder |
| **Category 3: Central Disorders of Hypersomnolence** |
| Narcolepsy type 1 |
| Narcolepsy type 2 |
| Idiopathic hypersomnia |
| Kleine-Levin syndrome |
| Hypersomnia due to a medical disorder |
| Hypersomnia due to a medication or substance |
| Hypersomnia associated with a psychiatric disorder |
| Insufficient sleep syndrome |
| **Category 4: Circadian Rhythm Sleep-Wake Disorders** |
| Delayed sleep-wake phase disorder |
| Advanced sleep-wake phase disorder |
| Irregular sleep-wake rhythm disorder |
| Non-24-h sleep-wake rhythm disorder |
| Shift work disorder |
| Jet lag disorder |
| Circadian sleep-wake disorder not otherwise specified |
| **Category 5: Parasomnias** |
| **Non rapid eye movement (NREM)-related parasomnias** |
| Confusional arousals |
| Sleepwalking |
| Sleep terrors |
| Sleep-related eating disorder |
| **REM-related parasomnias** |
| REM sleep behavior disorder |
| Recurrent isolated sleep paralysis |
| Nightmare disorder |
| **Other parasomnias** |
| Exploding head syndrome |
| Sleep-related hallucinations |
| Sleep enuresis |
| Parasomnia due to a medical disorder |
| Parasomnia due to a medication or substance |
| Parasomnia, unspecified |
| **Category 6: Sleep-Related Movement Disorders** |
| Restless legs syndrome |
| Periodic limb movement disorder |
| Sleep-related leg cramps |
| Sleep-related bruxism |
| Sleep-related rhythmic movement disorder |
| Benign sleep myoclonus of infancy |
| Propriospinal myoclonus at sleep onset |
| Sleep-related movement disorder due to a medical disorder |
| Sleep-related movement disorder due to a medication or substance |
| Sleep-related movement disorder, unspecified |
| **Category 7: Other sleep disorders** |
| Sleep disorders that could not be included in other categories |

ICSD = *International Classification of Sleep Disorders*

**Box S2: List of excluded studies**

1. More anthropology and less sleep for medical students1980 1980-12-20-27. 1662 p.

2. Al-Khani AM, Sarhandi MI, Zaghloul MS, Ewid M, Saquib N. A cross-sectional survey on sleep quality, mental health, and academic performance among medical students in Saudi Arabia. BMC research notes. 2019;12(1):665.

3. Almeida CM, Stabenow R, Lima P, Takatani M, Almeida C. M, Stabenow R, et al. Sleep in medical students of Amazonas - Brazil. JOURNAL OF THE NEUROLOGICAL SCIENCES. 2015;357:E173-E.

4. Almetrek M, Alqahtani M, Alsamghan A, Alqahtani H, Alshahrani M, Alshahrani S, et al. Sleep quality among male medical students in King Khalid University. JOURNAL OF THE NEUROLOGICAL SCIENCES. 2015;357:E173-E.

5. Almoajel A, Al-Zahrani AN, AL-Qtaibi MS, Almoajel Alia, Al-Zahrani Arwa Nasser, AL-Qtaibi Malak Saud. Health behaviours affecting academic performance among university students in Riyadh, Saudi Arabia: KSU female students as an example. AUSTRALASIAN MEDICAL JOURNAL. 2017;10(10):870-8.

6. Almohaya A, Qrmli A, Almagal N, Alamri K, Bahammam S, Al-Enizi M, et al. Sleep medicine education and knowledge among medical students in selected Saudi Medical Schools. BMC medical education. 2013;13:133.

7. Alóe F, Pedroso A, Tavares SM. Epworth Sleepiness Scale outcome in 616 Brazilian medical students. Brazil1997 1997-6. 220-6 p.

8. Alqahtani JS, Al-Harbi R, Bathnain B, Al-Ghamdi F, Al-Shehri I, AlRabeeah S, et al. Evaluation of Sleep Hygiene Among Medical Students in Saudi Medical College: An Exploratory Study. AMERICAN JOURNAL OF RESPIRATORY AND CRITICAL CARE MEDICINE. 2018;197.

9. Alsubhi AM, Yones DK, Almughazzawi K, Alqaedi RO, Alsharif ZI, Alsubhi Arwa Musaad, et al. FACTORS THAT CAUSE MEMORY IMPAIRMENT AMONG MEDICAL STUDENTS IN TAIBAH UNIVERSITY AT MEDINA, SAUDI ARABIA. INDO AMERICAN JOURNAL OF PHARMACEUTICAL SCIENCES. 2018;5(12):17264-81.

10. Alsuhaymi ZS, Alreheli AQ, Alawfi AM, ,, Alhazmi AM, Aljuhani RZ, Alruhaili EMS, et al. IMPACT OF SLEEP HABITS ON ACADEMIC ACHIEVEMENT AMONG FOURTH-YEAR MEDICAL STUDENTS AT TAIBAH UNIVERSITY. INDO AMERICAN JOURNAL OF PHARMACEUTICAL SCIENCES. 2019;6(1):790-800.

11. Alzunidi MA, Alowayyid JA, Benhadi RA, Farouk HH, Alshammari MH, Sultan AS, et al. Sleep Patterns and Academic Performance among Medicine and Pharm D students in Almaarefa University 2018-2019. WORLD FAMILY MEDICINE. 2022;20(1):130-45.

12. Anaya F, Abu Alia W, Hamoudeh F, Nazzal Z, Maraqa B. Epidemiological and clinical characteristics of headache among medical students in Palestine: a cross sectional study. BMC neurology. 2022;22(1):4.

13. Arbabisarjou A, Hashemi SM, Sharif MR, Haji Alizadeh K, Yarmohammadzadeh P, Feyzollahi Z. The Relationship between Sleep Quality and Social Intimacy, and Academic Burn-Out in Students of Medical Sciences. Global journal of health science. 2015;8(5):231-8.

14. Arzani-Birgani A, Zarei J, Favaregh L, Ghanaatiyan E. Internet addiction, mental health, and sleep quality in students of medical sciences, Iran: A cross-sectional study. Journal of education and health promotion. 2021;10:409.

15. Ayala EE, Berry R, Winseman JS, Mason HR. A Cross-Sectional Snapshot of Sleep Quality and Quantity Among US Medical Students. Academic psychiatry : the journal of the American Association of Directors of Psychiatric Residency Training and the Association for Academic Psychiatry. 2017;41(5):664-8.

16. Bilir F, Akdemir R, Bilir C. Some, but not all of the premenstrual syndrome symptoms affect the medical exam scores in medical students. Pakistan journal of medical sciences. 2021;37(4):1190-5.

17. Bosie GD, Tefera TW, Hailu GS, Bosie Getu Dadi, Tefera Tesfaye Wolde, Hailu Gebremedhin Solomon. Knowledge, attitude and practice with respect to sleep among undergraduate medical students of Mekelle University. SLEEP AND BIOLOGICAL RHYTHMS. 2012;10(4):264-9.

18. Casuccio A, Bonanno V, Catalano R, Cracchiolo M, Giugno S, Sciuto V, et al. Knowledge, Attitudes, and Practices on Energy Drink Consumption and Side Effects in a Cohort of Medical Students. Journal of addictive diseases. 2015;34(4):274-83.

19. Chan GC, Koh D. Understanding the psychosocial and physical work environment in a Singapore medical school. Singapore medical journal. 2007;48(2):166-71.

20. Cvejic E, Huang S, Vollmer-Conna U. Can you snooze your way to an 'A'? Exploring the complex relationship between sleep, autonomic activity, wellbeing and performance in medical students. The Australian and New Zealand journal of psychiatry. 2018;52(1):39-46.

21. Cvejic E, Parker G, Harvey SB, Steel Z, Hadzi-Pavlovic D, Macnamara CL, et al. The health and well-being of Australia's future medical doctors: protocol for a 5-year observational cohort study of medical trainees. BMJ open. 2017;7(9):e016837.

22. Dixit A, Mittal T. Executive Functions are not Affected by 24 Hours of Sleep Deprivation: A Color-Word Stroop Task Study. Indian journal of psychological medicine. 2015;37(2):165-8.

23. Domínguez Rodríguez A, Martinez-Maqueda GI, Arenas Landgrave P, Martínez Luna SC, Ramírez-Martínez FR, Salinas Saldivar JT. Effectiveness of behavioral activation for depression treatment in medical students: Study protocol for a quasi-experimental design. SAGE open medicine. 2020;8:2050312120946239.

24. Dzaferovic A, Ulen K, Dzaferovic A, Ulen K. Sleep habits among medical students and correlation between sleep quality and academic performance. EUROPEAN JOURNAL OF PUBLIC HEALTH. 2018;28:358-.

25. El Ansari W, Stock C, John J, Deeny P, Phillips C, Snelgrove S, et al. HEALTH PROMOTING BEHAVIOURS AND LIFESTYLE CHARACTERISTICS OF STUDENTS AT SEVEN UNIVERSITIES IN THE UK. CENTRAL EUROPEAN JOURNAL OF PUBLIC HEALTH. 2011;19(4):197-204.

26. Eleftheriou A, Rokou A, Arvaniti A, Nena E, Steiropoulos P, Eleftheriou Anna, et al. Sleep Quality and Mental Health of Medical Students in Greece During the COVID-19 Pandemic. FRONTIERS IN PUBLIC HEALTH. 2021;9.

27. Enkhtuya S, Jambal S, Davaadavga N, Jamsranjav A, Enkhtuya S, Jambal S, et al. Sleep disturbances Bamong medical students. JOURNAL OF THE NEUROLOGICAL SCIENCES. 2019;405.

28. Erdinc O, Unal E, Aydin R, Uzuner G, Metintas S, Erdinc O, et al. The relationship between excessive daytime sleepiness and depression in medical students. SLEEP MEDICINE. 2015;16:S102-S.

29. EVERETT HC, EVERETT HC. SLEEP PARALYSIS IN MEDICAL-STUDENTS. JOURNAL OF NERVOUS AND MENTAL DISEASE. 1963;136(3):283-7.

30. Eyuboglu M, Eyuboglu D, Duran O, Karademir SB, Karaaslan F, Alyu FM, et al. Depression, anxiety, sleep problems and suicidal behavior among medical students: A cross-sectional comparison study between first and sixth year students. KLINIK PSIKIYATRI DERGISI-TURKISH JOURNAL OF CLINICAL PSYCHIATRY. 2021;24(1):61-8.

31. Ezquiaga AD, Vas CP, Nieto GP, Blanco SA, Schwemler M, Roca L, et al. Sleep quality evaluation in medical students. JOURNAL OF THE NEUROLOGICAL SCIENCES. 2015;357:E435-E.

32. Feng GS, Chen JW, Yang XZ. [Study on the status and quality of sleep-related influencing factors in medical college students]. China2005 2005-5. 328-31 p.

33. Fino E, Martoni M, Russo PM. Specific mindfulness traits protect against negative effects of trait anxiety on medical student wellbeing during high-pressure periods. Advances in health sciences education : theory and practice. 2021;26(3):1095-111.

34. Frank E, Carrera JS, Elon L, Hertzberg VS. Basic demographics, health practices, and health status of U.S. medical students. American journal of preventive medicine. 2006;31(6):499-505.

35. Gacic J, Jovic SJ, Terzic NS, Cvetkovic VM, Terzic MT, Stojanovic DG, et al. Gender differences in stress intensity and coping strategies among students, future emergency relief specialists. VOJNOSANITETSKI PREGLED. 2021;78(6):635-41.

36. Galvan JAA, Sriram S, Chinna K, Bin Shukry MS, Khan NHBM, Sabri FBM, et al. LOW PREVALENCE OF OVERWEIGHT AND OBESITY AMONG MEDICAL STUDENTS AT A UNIVERSITY IN MALAYSIA. SOUTHEAST ASIAN JOURNAL OF TROPICAL MEDICINE AND PUBLIC HEALTH. 2019;50(6):1179-87.

37. Gangwar A, Tiwari S, Rawat A, Verma A, Singh K, Kant S, et al. Circadian Preference, Sleep Quality, and Health-impairing Lifestyles Among Undergraduates of Medical University. Cureus. 2018;10(6):e2856.

38. Gassara I, Ennaoui R, Halwani N, Turki M, Aloulou J, Amami O, et al. Sleep quality among medical students. EUROPEAN PSYCHIATRY. 2016;33:S594-S.

39. Gemnani VK, Shaikh AN, Mangrio RH, Tunio MI, Abbasi SA, Malik A, et al. Prevalence of daytime sleepiness and its impact on academic performane amongst the university students. RAWAL MEDICAL JOURNAL. 2020;45(4):959-62.

40. Geoca A, Dowling M, Jain V, Geoca A, Dowling M, Jain V. RELATIONSHIP BETWEEN CHRONOTYPE AND SLEEP DURATION AMONG MEDICAL STUDENTS. SLEEP. 2020;43:A298-A.

41. George M, Ahmed MS, George N, Simon S, George Meera, Ahmed Malik Shanawaz, et al. Internet: A Double-Edged Sword? - A Cross-Sectional Study. INDIAN JOURNAL OF MEDICAL SPECIALITIES. 2019;10(3):126-30.

42. Ghahramanyan L, Karamyan A, Budumyan A, Khachatryan S, Ghahramanyan L, Karamyan A, et al. Sleep complaints, sleep habits and their association with academic performance among medical students in Armenia. JOURNAL OF SLEEP RESEARCH. 2016;25:375-6.

43. Gomes AA, Tavares J, de Azevedo MHP, Gomes Ana Allen, Tavares Jose, de Azevedo Maria Helena P. Sleep and Academic Performance in Undergraduates: A Multi-measure, Multi-predictor Approach. CHRONOBIOLOGY INTERNATIONAL. 2011;28(9):786-801.

44. Gomes AA, Tavares J, De Azevedo MHP, Gomes Ana Allen, Tavares J, Pinto De Azevedo M. H. SLEEP-WAKE PATTERNS in Portuguese Undergraduates. ACTA MEDICA PORTUGUESA. 2009;22(5):545-52.

45. Gupta A, Jagzape A, Kumar M. Social media effects among freshman medical students during COVID-19 lock-down: An online mixed research. Journal of education and health promotion. 2021;10:55.

46. Gupta R, Taneja N, Anand T, Gupta A, Gupta R, Jha D, et al. Internet Addiction, Sleep Quality and Depressive Symptoms Amongst Medical Students in Delhi, India. COMMUNITY MENTAL HEALTH JOURNAL. 2021;57(4):771-6.

47. Hisler GC, Krizan Z, DeHart T. Does Stress Explain the Effect of Sleep on Self-Control Difficulties? A Month-Long Daily Diary Study. Personality & social psychology bulletin. 2019;45(6):864-77.

48. Hjiej G, Idrissi FEE, Janfi T, Bouhabs M, Hnaifi H, Belakbyer H, et al. Distant education in Moroccan medical schools following COVID-19 outbreak at the early phase of lockdown: Were the students really engaged? Scientific African. 2022;15:e01087.

49. Jaremków A, Markiewicz-Górka I, Pawlas K. Assessment of health condition as related to lifestyle among students in the examination period. International journal of occupational medicine and environmental health. 2020;33(3):339-51.

50. Jniene A, Errguig L, El Hangouche AJ, Rkain H, Aboudrar S, El Ftouh M, et al. Perception of Sleep Disturbances due to Bedtime Use of Blue Light-Emitting Devices and Its Impact on Habits and Sleep Quality among Young Medical Students. BioMed research international. 2019;2019:7012350.

51. L Hawick, J Cleland, S Kitto. 'I feel like I sleep here': how space and place influence medical student experiences. Medical education. 2018;52(10):1016-27.

52. L Jin, J Zhou, H Peng, S Ding, H Yuan. Investigation on dysfunctional beliefs and attitudes about sleep in Chinese college students. Neuropsychiatric disease and treatment. 2018;14:1425-32.

53. L Lins, FM Carvalho, MS Menezes, L Porto-Silva, H Damasceno. Health-related quality of life of students from a private medical school in Brazil. International journal of medical education. 2015;6:149-54.

54. L Lins, FM Carvalho, MS Menezes, L Porto-Silva, H Damasceno. Health-related quality of life of medical students in a Brazilian student loan programme. Perspectives on medical education. 2016;5(4):197-204.

55. Ł Mokros, A Witusik, J Michalska, W Łężak, M Panek, K Nowakowska-Domagała, et al. Sleep quality, chronotype, temperament and bipolar features as predictors of depressive symptoms among medical students. Chronobiology international. 2017;34(6):708-20.

56. L Machado, CTN Souza, RO Nunes, CN de Santana, CF Araujo, A Cantilino. Subjective well-being, religiosity and anxiety: a cross-sectional study applied to a sample of Brazilian medical students. Trends in psychiatry and psychotherapy. 2018;40(3):185-92.

57. L Paudel, P Sharma, AR Kadel, K Lakhey, S Singh, P Khanal, et al. Association Between Internet Addiction, Depression and Sleep Quality Among Undergraduate Students of Medical and Allied Sciences. Journal of Nepal Health Research Council. 2021;19(3):543-9.

58. L Talledo-Ulfe, OD Buitrago, Y Filorio, F Casanova, L Campos, F Cortés, et al. Factors associated with uninvestigated dyspepsia in students at 4 Latin American schools of medicine: A multicenter study. Revista de gastroenterologia de Mexico (English). 2018;83(3):215-22.

59. L Wang, P Qin, Y Zhao, S Duan, Q Zhang, Y Liu, et al. Prevalence and risk factors of poor sleep quality among Inner Mongolia Medical University students: A cross-sectional survey. Psychiatry research. 2016;244:243-8.

60. LA Fowler, S Ellis. The Effect of 12 Hour Shifts, Time of Day, and Sleepiness on Emotional Empathy and Burnout in Medical Students. Clocks & sleep. 2019;1(4):501-9.

61. LA Nogueira-Martins, R Fagnani Neto, PC Macedo, VA Cítero, JJ Mari. The mental health of graduate students at the Federal University of São Paulo: a preliminary report. Brazilian journal of medical and biological research = Revista brasileira de pesquisas medicas e biologicas. 2004;37(10):1519-24.

62. Lebedeva ER, Kobzeva NR, Gilev DV, Kislyak NV, Olesen J. Psychosocial factors associated with migraine and tension-type headache in medical students. Cephalalgia : an international journal of headache. 2017;37(13):1264-71.

63. Lemma S, Gelaye B, Berhane Y, Worku A, Williams MA, Lemma Seblewngel, et al. Sleep quality and its psychological correlates among university students in Ethiopia: a cross-sectional study. BMC PSYCHIATRY. 2012;12.

64. Lima AM, Kolodiuk FF, Ferreira LGF, Miguel MAL, Araujo JF, Lima A. M., et al. How bad is the sleep-wake cycle on first year medical students? JOURNAL OF SLEEP RESEARCH. 2014;23:249-.

65. Lima DVG, Kluthcovsky ACGC, Fernandes LGR, Okarenski G. Quality of sleep and use of computers and cell-phones among university students. Brazil2019 2019-12. 1454-8 p.

66. LM Fan, A Collins, L Geng, JM Li. Impact of unhealthy lifestyle on cardiorespiratory fitness and heart rate recovery of medical science students. BMC public health. 2020;20(1):1012.

67. LM Pop, M Iorga, LR Șipoș, R Iurcov. Gender Differences in Healthy Lifestyle, Body Consciousness, and the Use of Social Networks among Medical Students. Medicina (Kaunas, Lithuania). 2021;57(7).

68. LQ Qin, J Li, Y Wang, J Wang, JY Xu, T Kaneko. The effects of nocturnal life on endocrine circadian patterns in healthy adults. Life sciences. 2003;73(19):2467-75.

69. LT van Venrooij, PC Barnhoorn, EJ Giltay, MS van Noorden. Burnout, depression and anxiety in preclinical medical students: a cross-sectional survey. International journal of adolescent medicine and health. 2015;29(3).

70. Lupusor A, Zubco S, Vovc V, Lupusor A., Zubco S., Vovc V. Sleep quality of first year vs. sixth year medical students from the State University of Medicine and Pharmacy of the Republic of Moldova. JOURNAL OF SLEEP RESEARCH. 2018;27.

71. Luqman R, Ghous M, Nawaz J, Ali A, Kanwal M, Yaqoob I, et al. Factors associated with sleep deprivation and their impact on academic performance of hostelites of twin cities of Pakistan. JOURNAL OF THE PAKISTAN MEDICAL ASSOCIATION. 2020;70(5):851-5.

72. Lyshova OV, Lyshov VF, Lyshova O. V., Lyshov V. F. Sleep disturbances, daytime sleepiness and symptoms of sleep apnea syndrome in first year medical students. JOURNAL OF SLEEP RESEARCH. 2014;23:246-.

73. M Ansari. Sleeping Pattern of Medical Students Preceding Viva Examination and Their Performance. JNMA; journal of the Nepal Medical Association. 2015;53(200):262-5.

74. M Sarıaydın, E Günay, M Ünlü. [Frequency of restless legs syndrome and relationship between depression, anxiety and sleep quality among medical school students]. Tuberkuloz ve toraks. 2018;66(3):217-23.

75. M Saygın, Ö Öztürk, T Gonca, M Has, UB Hayri, Y Kurt, et al. Investigation of Sleep Quality and Sleep Disorders in Students of Medicine. Turkish thoracic journal. 2016;17(4):132-40.

76. M Shah, S Hasan, S Malik, CT Sreeramareddy. Perceived stress, sources and severity of stress among medical undergraduates in a Pakistani medical school. BMC medical education. 2010;10:2.

77. M Tanaka, K Mizuno, S Fukuda, Y Shigihara, Y Watanabe. Relationships between dietary habits and the prevalence of fatigue in medical students. Nutrition (Burbank, Los Angeles County, Calif). 2008;24(10):985-9.

78. M Tanaka, S Fukuda, K Mizuno, H Kuratsune, Y Watanabe. Stress and coping styles are associated with severe fatigue in medical students. Behavioral medicine (Washington, DC). 2009;35(3):87-92.

79. M Veldi, A Aluoja, V Vasar. Sleep quality and more common sleep-related problems in medical students. Sleep medicine. 2005;6(3):269-75.

80. MA Sawah, N Ruffin, M Rimawi, C Concerto, E Aguglia, E Chusid, et al. Perceived Stress and Coffee and Energy Drink Consumption Predict Poor Sleep Quality in Podiatric Medical Students A Cross-sectional Study. Journal of the American Podiatric Medical Association. 2015;105(5):429-34.

81. MA Safhi, RA Alafif, NM Alamoudi, MM Alamoudi, WA Alghamdi, SF Albishri, et al. The association of stress with sleep quality among medical students at King Abdulaziz University. Journal of family medicine and primary care. 2020;9(3):1662-7.

82. Ma XH, Meng DX, Zhu LW, Xu HY, Guo J, Yang LM, et al. Bedtime procrastination predicts the prevalence and severity of poor sleep quality of Chinese undergraduate students. JOURNAL OF AMERICAN COLLEGE HEALTH.

83. Maalej M, Guirat M, Mejdoub Y, Omri S, Feki R, Zouari L, et al. Quality of sleep, anxiety and depression among medical students during exams period: a cross sectional study. JOURNAL OF SLEEP RESEARCH. 2018;27.

84. Magali COJ, Ulises GSJ, Aveiro-Robalo TR, Daniela GTL, Valladares-Garrido MJ, Magali Coronel-Ocampos Johanna, et al. Association between sleep quality and sleep paralysis in medical students from a private university in Paraguay. PAKISTAN JOURNAL OF MEDICAL & HEALTH SCIENCES. 2020;14(3):1162-6.

85. Maghsoudi S, Amra B, Teimouri A, Maghsoudi Samin, Amra Babak, Teimouri Azam. The assessment of the correlation between sleep quality and irritable bowel syndrome among medical students. IMMUNOPATHOLOGIA PERSA. 2022;8(1).

86. Mahani S, Panchal P, Mahani Sundeep, Panchal Pavan. Evaluation of Knowledge, Attitude and Practice Regarding Stress Management among Undergraduate Medical Students at Tertiary Care Teaching Hospital. JOURNAL OF CLINICAL AND DIAGNOSTIC RESEARCH. 2019;13(8):FC5-FC9.

87. Maheshwari G, Shaukat F. Impact of Poor Sleep Quality on the Academic Performance of Medical Students. Cureus. 2019;11(4):e4357.

88. Mahfouz MS, Ali SA, Bahari AY, Ajeebi RE, Sabei HJ, Somaily SY, et al. Association Between Sleep Quality and Physical Activity in Saudi Arabian University Students. NATURE AND SCIENCE OF SLEEP. 2020;12:775-82.

89. Maia BR, Soares MJ, Gomes A, Marques M, Pereira AT, Valente J, et al. PERSONALITY TRAITS AND SLEEP PATTERNS/PROBLEMS IN MEDICAL STUDENTS. EUROPEAN PSYCHIATRY. 2009;24.

90. Maisuradze LM, Darchia N, Maisuradze L. M., Darchia N. Sleep habits and difficulties in Georgian medical students: preliminary data. JOURNAL OF SLEEP RESEARCH. 2008;17:138-.

91. Mansour AE, Almokhlef S, Alqifari R, Alduwayrij M, Mansour Ali E., Almokhlef Salman, et al. Lifestyle diseases and associated risk behaviours among medical students in Saudi Arabia. WORLD FAMILY MEDICINE. 2020;18(1):30-6.

92. Marques DR, Gomes AA, de Azevedo MHP, Marques Daniel Ruivo, Gomes Ana Allen, Pinto de Azevedo Maria Helena. Confirmatory Factor Analysis of the Portuguese Version of the Arousal Predisposition Scale. CURRENT PSYCHOLOGY. 2019;38(1):59-65.

93. Marques M, Soares MJ, Gomes A, Maia BR, Pereira AT, Valente J, et al. SLEEP PATTERNS AND HEALTH BEHAVIOURS IN MEDICAL STUDENTS. EUROPEAN PSYCHIATRY. 2009;24.

94. Maya SD, Lubert CD, Londono DMM, de la Portilla Maya Sonia, Dussan Lubert Carmen, Montoya Londono Diana Marcela. Characterization of sleep quality and excessive daytime sleepiness in a sample of students of the medical program of the University of Manizales (Colombia). ARCHIVOS DE MEDICINA. 2017;17(2):278-89.

95. MB Ali, T Warda, FZ Raza, T Laeeq, MB Ali, M Ali, et al. Knowledge and attitudes about burn complications in medical students. Burns : journal of the International Society for Burn Injuries. 2020;46(4):876-81.

96. MD Slivkoff, C Johnson, S Tackett. First-Year Medical Student Experiences Adjusting to the Immediate Aftermath of COVID-19. Medical science educator. 2021;31(2):1-8.

97. ME Kurtz, RD Paulsen, D Ferguson. How effectively are osteopathic medical students coping with a stressful life-style? The Journal of the American Osteopathic Association. 1990;90(7):613-22.

98. ME McCarthy, WF Waters. Decreased attentional responsivity during sleep deprivation: orienting response latency, amplitude, and habituation. United States1997 1997-2. 115-23 p.

99. ME Machado-Duque, JE Echeverri Chabur, JE Machado-Alba. [Excessive Daytime Sleepiness, Poor Quality Sleep, and Low Academic Performance in Medical Students]. Revista colombiana de psiquiatria. 2015;44(3):137-42.

100. Medeiros ALD, Mendes DBF, Lima PF, Araujo JF, Medeiros ALD, Mendes DBF, et al. The relationships between sleep-wake cycle and academic performance in medical students. BIOLOGICAL RHYTHM RESEARCH. 2001;32(2):263-70.

101. Mendes TB, de Souza KC, Franca CN, Rossi FE, Santos RPG, Duailibi K, et al. PHYSICAL ACTIVITY AND SYMPTOMS OF ANXIETY AND DEPRESSION AMONG MEDICAL STUDENTS DURING A PANDEMIC. REVISTA BRASILEIRA DE MEDICINA DO ESPORTE. 2021;27(6):582-7.

102. MF Martins, S Vanoni, VP Carlini. [Psychostimulants consumption for neuroenhancement among medical students from National University of Córdoba]. Revista de la Facultad de Ciencias Medicas (Cordoba, Argentina). 2020;77(4):254-9.

103. Milasauskiene E, Burkauskas J, Podlipskyte A, Király O, Demetrovics Z, Ambrasas L, et al. Compulsive Internet Use Scale: Psychometric Properties and Associations With Sleeping Patterns, Mental Health, and Well-Being in Lithuanian Medical Students During the Coronavirus Disease 2019 Pandemic. Frontiers in psychology. 2021;12:685137.

104. Nena E, Fasoulakis Z, Trypsianis G, Constantinidis TC, Kontomanolis E. Attitudes and thoughts of medical practitioners towards their profession in the era of financial crisis in Greece. Journal of preventive medicine and hygiene. 2020;61(1):E60-E5.

105. Ossai EN, Eze II, Onyenakazi RC, Ugebe E, Eze B, Obasi O. How large is the burden of depression in a medical school? A cross-sectional study among medical students in Nigeria. The Pan African medical journal. 2021;40:71.

106. Preišegolavičiūtė E, Leskauskas D, Adomaitienė V. Associations of quality of sleep with lifestyle factors and profile of studies among Lithuanian students. Switzerland2010 2010. 482-9 p.

107. Purohit G, Shah T, Harsoda JM. Prevalence of Obesity in Medical students and its correlation with cardiovascular risk factors: Emergency Alarm for Today? Kathmandu University medical journal (KUMJ). 2015;13(52):341-5.

108. Rique GL, Fernandes Filho GM, Ferreira AD, de Sousa-Muñoz RL. Relationship between chronotype and quality of sleep in medical students at the Federal University of Paraiba, Brazil. Sleep science (Sao Paulo, Brazil). 2014;7(2):96-102.

109. Schmidt G, Valdez M, Farrell M, Bishop F, Klam WP, Doan AP. Behaviors Associated with Internet Use in Military Medical Students and Residents. Military medicine. 2019;184(11):750-7.

110. Simonds GR, Marvin EA, Apfel LS, Elias Z, Howes GA, Witcher MR, et al. Clinical Neuroscience in Practice: An Experiential Learning Course for Undergraduates Offered by Neurosurgeons and Neuroscientists. Journal of undergraduate neuroscience education : JUNE : a publication of FUN, Faculty for Undergraduate Neuroscience. 2018;16(2):A112-A9.

111. Sivagnanam G, Thirumalaikolundusubramanian P, Sugirda P, Rajeswari J, Namasivayam K, Gitanjali B. Study of the knowledge, beliefs, and practice of sleep among medical undergraduates of Tamilnadu, India. MedGenMed : Medscape general medicine. 2004;6(4):5.

112. SMA Jahangeer, N Hasnain, MT Tariq, A Jamil, SY Zia, W Amir. Frequency and Association of Stress Levels with Modes of Commuting Among Medical Students of a Developing Country. The Malaysian journal of medical sciences : MJMS. 2021;28(4):113-22.

113. SN Pasha, UA Khan. Frequency of snoring and symptoms of sleep apnea among Pakistani medical students. Journal of Ayub Medical College, Abbottabad : JAMC. 2003;15(1):23-5.

114. Song YQ, Liu ZR, Chen HG, Guo Q, Huang YQ, Song Yuqing, et al. Incidence and Risk Factors of Depressive Symptoms in Chinese College Students. NEUROPSYCHIATRIC DISEASE AND TREATMENT. 2020;16:2449-57.

115. Soyakin B, Maharaj N, Jojua N, Sakhelashvili I, Soyakin B., Maharaj N., et al. SLEEP DISTURBANCES AND STRESS AMONG THE FOREIGN MEDICAL STUDENTS OF EUROPEAN UNIVERSITY, GEORGIA. SLEEP MEDICINE. 2019;64:S331-S2.

116. SP Behere, R Yadav, PB Behere. A comparative study of stress among students of medicine, engineering, and nursing. Indian journal of psychological medicine. 2011;33(2):145-8.

117. SP Dharmadhikari, SD Harshe, PP Bhide. Prevalence and Correlates of Excessive Smartphone Use among Medical Students: A Cross-sectional Study. Indian journal of psychological medicine. 2019;41(6):549-55.

118. SR Daugherty, Jr Baldwin DC. Sleep deprivation in senior medical students and first-year residents. Academic medicine : journal of the Association of American Medical Colleges. 1996;71(1):S93-5.

119. SS Sullivan, MT Cao. Sleep and Health: Medical Students' Perspectives and Lessons Learned. Academic psychiatry : the journal of the American Association of Directors of Psychiatric Residency Training and the Association for Academic Psychiatry. 2017;41(5):679-81.

120. Stores G, Crawford C. Medical student education in sleep and its disorders. Journal of the Royal College of Physicians of London. 1998;32(2):149-53.

121. Sui G, Liu G, Jia L, Wang L, Yang G. The association between ambient air pollution exposure and mental health status in Chinese female college students: a cross-sectional study. Environmental science and pollution research international. 2018;25(28):28517-24.

122. Sun JB, Chen M, Cai WJ, Wang Z, Wu SN, Sun X, et al. Chronotype: implications for sleep quality in medical students. CHRONOBIOLOGY INTERNATIONAL. 2019;36(8):1115-23.

123. Supangat, EN Sakinah, MY Nugraha, TS Qodar, BW Mulyono, AI Tohari. COVID-19 Vaccines Programs: adverse events following immunization (AEFI) among medical Clerkship Student in Jember, Indonesia. BMC pharmacology & toxicology. 2021;22(1):58.

124. Swamy RS, Kumar N, Adnan FS, Yacob FNM, Ismail FN, Samsuddin H, et al. Effect of light exposure during sleep on the curricular and extracurricular activities of medical students. BANGLADESH JOURNAL OF MEDICAL SCIENCE. 2017;16(4):541-4.

125. Syafriani N, Lailiyya N, Nurhayati T, Syafriani N., Lailiyya N., Nurhayati T. The prevalence of insomnia and the description of sleep hygiene in medical students, Universitas Padjadjaran, Indonesia. SLEEP MEDICINE. 2015;16:S104-S.

126. V Leichtfried, G Putzer, D Perkhofer, W Schobersberger, A Benzer. Circadian melatonin profiles during single 24-h shifts in anesthetists. Sleep & breathing = Schlaf & Atmung. 2011;15(3):503-12.

127. V Singh, S Pandey, A Singh, R Gupta, R Prasad, MP Singh Negi. Study pattern of snoring and associated risk factors among medical students. Bioscience trends. 2012;6(2):57-62.

128. VA Kumar, V Chandrasekaran, H Brahadeeswari. Prevalence of smartphone addiction and its effects on sleep quality: A cross-sectional study among medical students. Industrial psychiatry journal. 2019;28(1):82-5.

129. Vajda C, Czernin M, Matzer F, Schenkeli E, Lorenzoni N, Fazekas C, et al. Gender related difference in sleep quality and tiredness in Austrian medical students. EUROPEAN JOURNAL OF PUBLIC HEALTH. 2017;27.

130. van den Berg JF, Kivela L, Antypa N, van den Berg Julia F., Kivela Liia, Antypa Niki. Chronotype and depressive symptoms in students: An investigation of possible mechanisms. CHRONOBIOLOGY INTERNATIONAL. 2018;35(9):1248-61.

131. Vardar E, Vardar SA, Molla T, Kaynak C, Ersoz E, Vardar Erdal, et al. Psychological symptoms and sleep quality in young subjects with different circadian preferences. BIOLOGICAL RHYTHM RESEARCH. 2008;39(6):493-500.

132. Venkatesan N, Nanda R, Patel S, Mohapatra E, Venkatesan Nanditha, Nanda Rachita, et al. Impact of Sleep Patterns on Glycaemic Levels in Healthy Young Adults. JOURNAL OF EVOLUTION OF MEDICAL AND DENTAL SCIENCES-JEMDS. 2020;9(47):3533-7.

133. Villanueva EW, Meissner H, Walters RW. Medical Student Perceptions of the Learning Environment, Quality of Life, and the School of Medicine's Response to the COVID-19 Pandemic: A Single Institution Perspective. Medical science educator. 2021;31(2):589-98.

134. Vizcarra DR, Delgado RN, Meza MS, Escobar F, Vizcarra DR, Delgado RN, et al. Sleep knowledge in a sample of Latin-American medical students. SLEEP. 2002;25:A514-A.

135. VO Korobchansky, YO Oliinyk, VG Nesterenko, VV Sarkis-Ivanova, OV Hryhorian. HYGIENIC ASPECTS OF LIFESTYLE OF KHARKIV NATIONAL MEDICAL UNIVERSITY JUNIOR STUDENTS IN THE CONDITIONS OF QUARANTINE. Wiadomosci lekarskie (Warsaw, Poland : 1960). 2021;74(3):736-40.

136. Vo TQ, Nguyen HTT, Ta APN, Vo Trung Quang, Nguyen Hieu Thanh Thi, Ta Anh Phuong Ngoc. Effect of sociodemographic factors on quality of life of medical students in southern Vietnam: A survey using the WHOQOL-BREF assessment. JOURNAL OF PHARMACY & PHARMACOGNOSY RESEARCH. 2020;8(3):211-24.

137. Vohra M, AlSuwaine B, AlTulaiqi W, AlOtaibi T, AlShehri M, AlQahtani A, et al. THE ASSOCIATION BETWEEN CAFFEINE CONSUMPTION AND SLEEPING HABITS AMONG MEDICAL STUDENTS. INDO AMERICAN JOURNAL OF PHARMACEUTICAL SCIENCES. 2019;6(3):4913-21.

138. Volpp KG, Shea JA, Small DS, Basner M, Zhu JS, Norton L, et al. Effect of a Protected Sleep Period on Hours Slept During Extended Overnight In-hospital Duty Hours Among Medical Interns A Randomized Trial. JAMA-JOURNAL OF THE AMERICAN MEDICAL ASSOCIATION. 2012;308(21):2208-17.

139. W Cai, S Chen, L Li, P Yue, X Yu, L Gao, et al. Gender-specific physical activity-related injuries and risk factors among university students in China: a multicentre population-based cross-sectional study. BMJ open. 2020;10(12):e040865.

140. W Chen, YD Shen, R Chen, GX Ding. [Investigation on sleep status of college and high school students]. China2005 2005-1. 48-50 p.

141. W Naseer, O Gul, H Saeed, FH Qizilbash, Q Jawed, SF Mohsin, et al. Assessment and comparison of sleep patterns among medical and non-medical undergraduates of Karachi: A cross-sectional study - SPECIAL REPORT. JPMA The Journal of the Pakistan Medical Association. 2019;69(6):917-21.

142. W Wu, Y Zhang, P Wang, L Zhang, G Wang, G Lei, et al. Psychological stress of medical staffs during outbreak of COVID-19 and adjustment strategy. Journal of medical virology. 2020;92(10):1962-70.

143. Wali RM, Bagabas TM, Hassanein AA, SaadAlameri M, Al Ouqla KF, Wali Razaz Mohammed, et al. The Prevalence and Risk Factors of Depression and Anxiety Disorders among Medical Students in King Saud Bin Abdulaziz University for Health Sciences, Jeddah 2019. ANNALS OF MEDICAL AND HEALTH SCIENCES RESEARCH. 2021;11:1-6.

144. Wang YQ, Zhao Y, Liu L, Chen Y, Ai D, Yao YS, et al. The Current Situation of Internet Addiction and Its Impact on Sleep Quality and Self-Injury Behavior in Chinese Medical Students. PSYCHIATRY INVESTIGATION. 2020;17(3):237-42.

145. Wang YQ, Zhao Y, Liu L, Chen Y, Ai D, Yao YS, et al. The Current Situation of Internet Addiction and Its Impact on Sleep Quality and Self-Injury Behavior in Chinese Medical Students (vol 17, pg 237, 2020). PSYCHIATRY INVESTIGATION. 2020;17(4):385-.

146. Webb E, Ashton CH, Kelly P, Kamali F, Webb E, Ashton CH, et al. Alcohol and drug use in UK university students. LANCET. 1996;348(9032):922-5.

147. Wells M, Roth L, McWilliam M, Thompson K, Chande N, Wells Malcolm, et al. A cross-sectional study of the association between overnight call and irritable bowel syndrome in medical students. CANADIAN JOURNAL OF GASTROENTEROLOGY AND HEPATOLOGY. 2012;26(5):281-4.

148. WM Sweileh, AF Sawalha, SH Zyoud, SW Al-Jabi, FF Shamseh, HS Khalaf. Epidemiological, clinical and pharmacological aspects of headache in a university undergraduate population in Palestine. Cephalalgia : an international journal of headache. 2010;30(4):439-46.

149. Wojdak-Haasa E, Zarzeczna-Baran M, Pegiel-Kamrat J. [Ways of spending free time by students of the Medical University of Gdansk in relation to their health behavior]. Poland2002 2002. 565-70 p.

150. X Guo, T Su, H Xiao, R Xiao, Z Xiao. Using 24-h Heart Rate Variability to Investigate the Sleep Quality and Depression Symptoms of Medical Students. Frontiers in psychiatry. 2021;12:781673.

151. X Gu, Y Xie. Migraine attacks among medical students in Soochow University, Southeast China: a cross-sectional study. Journal of pain research. 2018;11:771-81.

152. X Zhong, Y Liu, J Pu, L Tian, S Gui, X Song, et al. Depressive symptoms and quality of life among Chinese medical postgraduates: a national cross-sectional study. Psychology, health & medicine. 2019;24(8):1015-27.

153. XF Pan, Y Wen, Y Zhao, JM Hu, SQ Li, SK Zhang, et al. Prevalence of depressive symptoms and its correlates among medical students in China: a national survey in 33 universities. Psychology, health & medicine. 2016;21(7):882-9.

154. Y Ge, S Xin, D Luan, Z Zou, M Liu, X Bai, et al. Association of physical activity, sedentary time, and sleep duration on the health-related quality of life of college students in Northeast China. Health and quality of life outcomes. 2019;17(1):124.

155. Y Gao, W Cai, L Gao, J Wang, J Liang, H Kwok, et al. Physical activity-related injuries among university students: a multicentre cross-sectional study in China. BMJ open. 2018;8(9):e021845.

156. Z Yazdi, Z Loukzadeh, P Moghaddam, S Jalilolghadr. Sleep Hygiene Practices and Their Relation to Sleep Quality in Medical Students of Qazvin University of Medical Sciences. Journal of caring sciences. 2016;5(2):153-60.

157. Zafar M, Ansari K, Zafar Mubashir, Ansari Khaled. Sleep Disorders Among Undergraduate Health Students in Bristol, United Kingdom. MEDITERRANEAN JOURNAL OF CLINICAL PSYCHOLOGY. 2020;8(3).

158. Zahid S, Surani A, Khaliqdina SJ, Surani AA, Khaliqdina SAJ, Surani S, et al. Sleep quality among medical students in Karachi, Pakistan. EUROPEAN JOURNAL OF MEDICAL RESEARCH. 2010;15:204-.

159. Zaidi GA, Rehman ST, Shafiq MM, Zehra T, Israar M, Hussain SM. Knowledge of obstructive sleep apnoea in final year medical students and junior doctors-a multi-centre cross-sectional study. The clinical respiratory journal. 2021;15(3):345-50.

160. Zarea K, Rahmani M, Hassani F, Hakim A, Zarea Kourosh, Rahmani Mahnaz, et al. Epidemiology and associated factors of migraine headache among iranian medical students: A descriptive-analytical study. CLINICAL EPIDEMIOLOGY AND GLOBAL HEALTH. 2018;6(3):109-14.

161. Zarnoog SS, Al Omrani AN, Alrumaih AA, Alabdullatif HA, Almarri FK, Alshareef RI, et al. Prevalence of irritable bowel syndrome and its associated risk factors among medical students in Riyadh, Saudi Arabia: A cross sectional study. MEDICAL SCIENCE. 2021;25(118):3479-88.

162. Zdun-Ryzewska A, Nadrowska N, Basinski K, Walkiewicz M, Blazek M, Zdun-Ryzewska Agata, et al. Who is a tired student? Fatigue and its predictors from a gender perspective. JOURNAL OF UNIVERSITY TEACHING AND LEARNING PRACTICE. 2021;18(6).

163. Zeb NG, Zareen N, Asra WA, Ahamed KA, Zeb Nida Gulzar, Zareen Nusrat, et al. Correlation between sleep habits and academic performance in medical students of Majmaah University, Kingdom of Saudi Arabia. RAWAL MEDICAL JOURNAL. 2020;45(1):201-5.

164. Zeekash S, Ali A, Asif F, Zeekash Sana, Ali Ahsan, Asif Faryal. FACTORS CAUSING GENERALIZED ANXIETY DISORDER IN MEDICAL STUDENTS ACCORDING TO ICD-10 CRITERIA. INDO AMERICAN JOURNAL OF PHARMACEUTICAL SCIENCES. 2018;5(10):10236-46.

165. Zhai XY, Ye M, Wang C, Gu Q, Huang T, Wang K, et al. Associations among physical activity and smartphone use with perceived stress and sleep quality of Chinese college students. MENTAL HEALTH AND PHYSICAL ACTIVITY. 2020;18.

166. Zhang CH, Li G, Fan ZY, Tang XJ, Zhang F, Zhang Chang-hong, et al. Mobile Phone Addiction Mediates the Relationship Between Alexithymia and Learning Burnout in Chinese Medical Students: A Structural Equation Model Analysis. PSYCHOLOGY RESEARCH AND BEHAVIOR MANAGEMENT. 2021;14:455-65.

167. Zhang LY, Wu JY, Deng CY, Zhang MM, Li CJ, Wang Q, et al. Mental health and personality implications among medical students during the outbreak of the COVID-19 pandemic. SOCIAL BEHAVIOR AND PERSONALITY. 2021;49(8).

168. Zhang X, Gao F, Kang Z, Zhou HG, Zhang JF, Li JJ, et al. Perceived Academic Stress and Depression: The Mediation Role of Mobile Phone Addiction and Sleep Quality. FRONTIERS IN PUBLIC HEALTH. 2022;10.

169. Ziade NR, Fayad F, Badra B, Ziade N. R., Fayad F., Badra B. LOW BACK PAIN IN MEDICAL STUDENTS LINKED TO POOR SLEEP QUALITY: RESULTS FROM THE PAX-I STUDY. ANNALS OF THE RHEUMATIC DISEASES. 2017;76:1001-.

170. Abdelaziz AMY, Alotaibi KT, Alhurayyis JH, Alqahtani TA, Alghamlas AM, Algahtani HM, et al. The association between physical symptoms and depression among medical students in Bahrain. International journal of medical education. 2017;8:423-7.

171. Abdulah Deldar Morad, Piro Rasoul Sabri. Sleep disorders as primary and secondary factors in relation with daily functioning in medical students. Annals of Saudi medicine. 2018;38(1):57-64.

172. Abdulghani HM, Al-Drees AA, Khalil MS, Ahmad F, Ponnamperuma GG, Amin Z. What factors determine academic achievement in high achieving undergraduate medical students? A qualitative study. Medical teacher. 2014;36:S43-8.

173. Afzal H, Afzal S, Siddique SA, Naqvi SA. Measures used by medical students to reduce test anxiety. JPMA The Journal of the Pakistan Medical Association. 2012;62(9):982-6.

174. Agarwal G, Mosquera M, Ring M, Victorson D. Work engagement in medical students: An exploratory analysis of the relationship between engagement, burnout, perceived stress, lifestyle factors, and medical student attitudes. Medical teacher. 2020;42(3):299-305.

175. Aldahash FD, Alasmari SA, Alnomsi SJ, Alshehri AM, Alharthi NF, Aloufi AAH, et al. Relationship of body mass index to sleep duration, and current smoking among medical students in Tabuk City, Saudi Arabia. Electronic physician. 2018;10(9):7273-8.

176. Amelot A, Mathon B, Haddad R, Renault MC, Duguet A, Steichen O. Low Back Pain Among Medical Students: A Burden and an Impact to Consider! Spine. 2019;44(19):1390-5.

177. Amelot A, Mathon B, Haddad R, Renault MC, Duguet A, Steichen O, et al. Low Back Pain Among Medical Students. SPINE. 2019;44(19):1390-5.

178. Anjum MU, Shahid MU, Iqbal MR, Anjum Muhammad Usama, Shahid Muhammad Usman, Iqbal Muhammad Roshaan. A CROSS-SECTIONAL RESEARCH TO ASSESS THE DEPENDENCE OF EDUCATIONAL EFFICIENCY ON SLEEPING PATTERN AMONG MEDICAL STUDENTS. INDO AMERICAN JOURNAL OF PHARMACEUTICAL SCIENCES. 2019;6(4):7152-5.

179. Anuradha R, Priyadharshini S, Patil A, Anuradha R, Priyadharshini S, Patil Aruna. Lifestyle Behaviour among Undergraduate Medical Students in Tamil Nadu: A Cross- sectional Study. JOURNAL OF CLINICAL AND DIAGNOSTIC RESEARCH. 2021;15(10):LC1-LC4.

180. Arif A, Iram M, Nofal S, Sohail A, Riaz B, Ahmed S, et al. Prevalence of Depression, Anxiety and Stress in Medical Students, interns and residents in Pakistan. PAKISTAN JOURNAL OF MEDICAL & HEALTH SCIENCES. 2021;15(9):2333-6.

181. Arif M, Hussain R, Nazir A, Wasim AZ, Khan AR, Arif K, et al. 4th year MBBS Students: Knowledge and Practice for Energy Drinks Consumption and their Side Effects. PAKISTAN JOURNAL OF MEDICAL & HEALTH SCIENCES. 2018;12(4):1405-9.

182. Arshad D, Joyia UM, Fatima S, Khalid N, Rishi AI, Rahim NUA, et al. The adverse impact of excessive smartphone screen-time on sleep quality among young adults: A prospective cohort. Sleep science (Sao Paulo, Brazil). 2021;14(4):337-41.

183. Aslam HM, Mughal A, Edhi MM, Saleem S, Rao MH, Aftab A, et al. Assessment of pattern for consumption and awareness regarding energy drinks among medical students. Archives of public health = Archives belges de sante publique. 2013;71(1):31.

184. Backović DV, Maksimović M, Davidović D, Zivojinović JI, Stevanović D. [Stress and mental health among medical students]. Srpski arhiv za celokupno lekarstvo. 2013;141(11):780-4.

185. Bandla H, Franco R, Statza T, Feroah T, Rice TB, Poindexter K, et al. Integrated selective: an innovative teaching strategy for sleep medicine instruction for medical students. Sleep medicine. 2007;8(2):144-8.

186. Blasche G, Zilic J, Frischenschlager O. Task-related increases in fatigue predict recovery time after academic stress. Journal of occupational health. 2016;58(1):89-95.

187. Braga PB, Lacerda AHBB, Lopes AT, Ventura A, Vigna NS, Braga FM, et al. Daily sleep duration and recurrent headaches in medical students. CEPHALALGIA. 2011;31(1):128-.

188. Brick CA, Seely DL, Palermo TM. Association between sleep hygiene and sleep quality in medical students. Behavioral sleep medicine. 2010;8(2):113-21.

189. Brockmann P, Nunez F, Brockmann P, Nunez F. SEASONAL CHANGES IN SOMNOLENCE, CHRONOTYPE, AND PUPILLOGRAPHIC INDICES IN MEDICAL STUDENTS. SLEEP MEDICINE. 2017;40:E42-E.

190. Butt AH, Bashir MM, Hassan U, Butt Abdul Haseeb, Bashir Muhammad Mubeen, Hassan Umair. CROSS SECTIONAL STUDY ON THE QUALITY AND PATTERNS OF SLEEP IN RELATION TO CONSUMPTION OF ENERGY DRINKS AMONG STUDENTS. INDO AMERICAN JOURNAL OF PHARMACEUTICAL SCIENCES. 2018;5(8):7741-5.

191. Caglar S, Kesgin MT, Coskun H, Caglar Songul, Tokur Kesgin Makbule, Coskun Hamit. The validity and reliability study of the Cleveland Adolescent Sleepiness Questionnaire and examination of the sleepiness of university students. PERSPECTIVES IN PSYCHIATRIC CARE.

192. Cai ZZ, Jin M, Zu TT, Yang PP, Cai Zhenzhen, Jin Ming, et al. Study on the Relationship between Medical Students' Worry Level, Coping Style and Sleep. 29 AVENUE LAVMIERE, PARIS, 75019, FRANCE: ATLANTIS PRESS; 2016 2016. 99-104 p.

193. Casuso-Holgado MJ, Moreno-Morales N, Labajos-Manzanares MT, Montero-Bancalero FJ, Jesus Casuso-Holgado Ma, Moreno-Morales Noelia, et al. The association between perceived health symptoms and academic stress in Spanish Higher Education students. EUROPEAN JOURNAL OF EDUCATION AND PSYCHOLOGY. 2019;12(2):109-23.

194. Chen CY, Yu NW, Huang TH, Wang WS, Fang JT. Harm avoidance and depression, anxiety, insomnia, and migraine in fifth-year medical students in Taiwan. Neuropsychiatric disease and treatment. 2018;14:1273-80.

195. Cheng S, Jia CX, Wang YJ, Cheng Shuo, Jia Cunxian, Wang Yongjie. Only Children Were Associated with Anxiety and Depressive Symptoms among College Students in China. INTERNATIONAL JOURNAL OF ENVIRONMENTAL RESEARCH AND PUBLIC HEALTH. 2020;17(11).

196. Chia CF, Lai JHY, Cheung PK, Kwong LT, Lau FPM, Leung KH, et al. Dysmenorrhoea among Hong Kong university students: prevalence, impact, and management. HONG KONG MEDICAL JOURNAL. 2013;19(3):222-8.

197. Chia CF, Lai JH, Cheung PK, Kwong LT, Lau FP, Leung KH, et al. Dysmenorrhoea among Hong Kong university students: prevalence, impact, and management. Hong Kong medical journal = Xianggang yi xue za zhi. 2013;19(3):222-8.

198. Choi HJ, Lee YJ, Cho YW, Moon HJ, Choi Hyun Ji, Lee Yeo Jin, et al. The effects of chronotype and social jetlag on medical students. SLEEP AND BIOLOGICAL RHYTHMS. 2019;17(3):269-76.

199. Chrzanowska D, Wdowiak L, Bojar I. The origin of stress, its causes, symptoms and frequency of appearance among the students of Medical University of Lublin. Annales Universitatis Mariae Curie-Sklodowska Sectio D: Medicina. 2004;59(1):438-43.

200. Chutani A, Shenvi DN, Singhal A, Chutani Arun, Shenvi D. N, Singhal Anita. Sleep, Sleepiness and Medical College Students: A Comparative Study among Medical and Paramedical Students of a Tertiary Care Teaching Hospital from a West Indian Metropolitan City. ANNALS OF MEDICAL AND HEALTH SCIENCES RESEARCH. 2017;7(2):85-91.

201. Corrêa CC, Oliveira FK, Pizzamiglio DS, Ortolan EVP, Weber SAT. Sleep quality in medical students: a comparison across the various phases of the medical course2017 2017-7. 285-9 p.

202. Costa LS, Marins MC, Ansel JC, Tavares CP, Queiroz FT, Rocha JB, et al. Sleep Quality in Medical Students A Cross-sectional Study. HYPERTENSION. 2017;70.

203. Cruz-Sanchez RA, Ferrer-Juarez LE, Reyes-Garduno ME, Gil-Trejo MJ, Sanchez-Moguel SM, Gonzalez-Salinas S, et al. Short-term changes in the mental health of recently enrolled university students. JOURNAL OF MENTAL HEALTH AND HUMAN BEHAVIOUR. 2019;24(2):126-31.

204. da Silva RCD, Garcez A, Pattussi MP, Olinto MTA, Dutra da Silva Renato Canevari, Garcez Anderson, et al. Prevalence and factors associated with excessive and severe daytime sleepiness among healthcare university students in the Brazilian Midwest. JOURNAL OF SLEEP RESEARCH.

205. Dabek J, Skorus P, Lepich T, Bajor G, Gasior Z, Dabek Jozefa, et al. KNOWLEDGE OF CORONARY ARTERIOSCLEROSIS RISK FACTORS AND THEIR OCCURRENCE AND THE LIFESTYLES OF THE FIRST-YEAR MEDICAL STUDENTS. HEALTH PROBLEMS OF CIVILIZATION. 2018;12(2):78-87.

206. Doos Ali Vand H, Gharraee B, Farid AA, Bandi MG. Prediction of insomnia severity based on cognitive, metacognitive, and emotional variables in college students. Explore (New York, NY). 2014;10(4):233-40.

207. Duan H, Gong M, Zhang Q, Huang X, Wan B. Research on sleep status, body mass index, anxiety and depression of college students during the post-pandemic era in Wuhan, China. Journal of affective disorders. 2022;301:189-92.

208. Duran-Aguero S, Sepulveda R, Guerrero-Wyss M, Duran-Aguero Samuel, Sepulveda Ricardo, Guerrero-Wyss Marion. Sleep disorders and anthropometric measures in Chilean university students. REVISTA ESPANOLA DE NUTRICION HUMANA Y DIETETICA. 2019;23(3):153-61.

209. ElHawary H, Salimi A, Barone N, Alam P, Thibaudeau S. The effect of COVID-19 on medical students' education and wellbeing: a cross-sectional survey. Canadian medical education journal. 2021;12(3):92-9.

210. Fadl AFB, Al-Towerqi AM, Alharbi AA, Kabrah DK, Almalki AA, Algethami BN, et al. Stress and a sedentary lifestyle are associated with irritable bowel syndrome in medical students from Saudi Arabia. WORLD FAMILY MEDICINE. 2022;20(1):101-8.

211. Falloon K, Wearn A, Bhoopatkar H, Nakatsuji M, Moir F, Falloon K, et al. Sleep quality in medical students - A New Zealand perspective. JOURNAL OF SLEEP RESEARCH. 2019;28.

212. Fan AP, Kosik RO, Mandell GA, Tran DT, Cheng HM, Chen CH, et al. Suicidal ideation in medical students: who is at risk? Annals of the Academy of Medicine, Singapore. 2012;41(9):377-82.

213. Fanjaud G, Calvet U, Rous de Feneyrols A, Barrere M, Bes A, Arbus L. [The role of paradoxical sleep in learning in man]. Revue d'electroencephalographie et de neurophysiologie clinique. 1982;12(4):337-43.

214. Faris E, Jahrami H, Al-Hilali MM, Chehyber NJ, Ali SO, Shahda SD, et al. Energy drink consumption is associated with reduced sleep quality among college students: a cross-sectional study. NUTRITION & DIETETICS. 2017;74(3):268-74.

215. Fekih-Romdhane F, ElKhouni C, Sassi H, Cheour M. The Role of Personal Factors and Learning Environment in Suicidal Ideation Among Tunisian Medical Students. Crisis. 2021;42(1):20-31.

216. FitzGerald LZ, Boland D, FitzGerald Leah Z, Boland David. The Impact of Behavioral and Psychological Factors on Physical Fitness in Medical and Nursing Students. HOLISTIC NURSING PRACTICE. 2018;32(3):125-32.

217. Fowler LA, Ellis SM, Fowler L. A, Ellis S. M. THE EFFECT OF 12-HOUR EMT SHIFTS ON MEDICAL STUDENT FATIGUE, EMPATHY AND BURNOUT. SLEEP MEDICINE. 2019;64:S118-S9.

218. Grady F, Roberts LW. Sleep Deprived and Overwhelmed: Sleep Behaviors of Medical Students in the USA. United States2017 2017-10. 661-3 p.

219. Gruba G, Kasiak PS, Gębarowska J, Adamczyk N, Sikora Z, Jodczyk AM, et al. PaLS Study of Sleep Deprivation and Mental Health Consequences of the COVID-19 Pandemic among University Students: A Cross-Sectional Survey. International journal of environmental research and public health. 2021;18(18).

220. Haghighi M, Gerber M, Haghighi Mohammad, Gerber Markus. Does Mental Toughness Buffer the Relationship Between Perceived Stress, Depression, Burnout, Anxiety, and Sleep? INTERNATIONAL JOURNAL OF STRESS MANAGEMENT. 2019;26(3):297-305.

221. Haile YG, Alemu SM, Habtewold TD, Haile Yohannes Gebreegziabhere, Alemu Sisay Mulugeta, Habtewold Tesfa Dejenie. Insomnia and Its Temporal Association with Academic Performance among University Students: A Cross-Sectional Study. BIOMED RESEARCH INTERNATIONAL. 2017;2017.

222. Haile YG, Alemu SM, Habtewold TD, Haile Yohannes Gebreegziabhere, Alemu Sisay Mulugeta, Habtewold Tesfa Dejenie. Common mental disorder and its association with academic performance among Debre Berhan University students, Ethiopia. INTERNATIONAL JOURNAL OF MENTAL HEALTH SYSTEMS. 2017;11.

223. Haj-Yahia MM, de Zoysa P, Haj-Yahia Muhammad M, de Zoysa Piyanjli. Rates and psychological effects of exposure to family violence among Sri Lankan university students. CHILD ABUSE & NEGLECT. 2008;32(10):994-1002.

224. Hanan R, Shafi MW, Anwar S, Nawaz N, Hanan Rana, Shafi Muhammad Waqar, et al. Hypertension and Pre-Hypertension with Associated Risk Factors Among MBBS Students of Shalamar Medical and Dental College, Lahore. ANNALS OF KING EDWARD MEDICAL UNIVERSITY LAHORE PAKISTAN. 2017;23(4):477-82.

225. Haq IU, Mariyam Z, Zeb F, Jiang P, Wu XY, Shah J, et al. Identification of Body Composition, Dietary Patterns and Its Associated Factors in Medical University Students in China. ECOLOGY OF FOOD AND NUTRITION. 2020;59(1):65-78.

226. Haque M, Rahman NAA, Majumder MAA, Rahman NIA, Haque SZ, Zulkifli Z, et al. Assessment of Academic/Non-Academic Factors and Extracurricular Activities Influencing Performance of Medical Students of Faculty of Medicine, Universiti Sultan Zainal Abidin, Malaysia. ADVANCES IN HUMAN BIOLOGY. 2018;8(1):3-18.

227. Haregu A, Gelaye B, Pensuksan WC, Lohsoonthorn V, Lertmaharit S, Rattananupong T, et al. Circadian rhythm characteristics, poor sleep quality, daytime sleepiness and common psychiatric disorders among Thai college students. ASIA-PACIFIC PSYCHIATRY. 2015;7(2):182-9.

228. Harmat L, Takacs J, Bodizs R, Harmat L, Takacs J, Bodizs R. Music improves sleep quality in medical students. JOURNAL OF SLEEP RESEARCH. 2006;15:133-4.

229. Hasan MT, Hossain S, Das Gupta R, Podder V, Mowri NA, Ghosh A, et al. Depression, sleeping pattern, and suicidal ideation among medical students in Bangladesh: a cross-sectional pilot study. JOURNAL OF PUBLIC HEALTH-HEIDELBERG. 2022;30(2):465-73.

230. Henry M, Huerga-Garcia L, Perez-Morell AA, Henry-Gonzalez AA, Henry M, Huerga-Garcia L, et al. Pittsburgh Sleep Quality Index in younger medical students. Are they poor sleepers and do they consume sleeping medication? EUROPEAN NEUROPSYCHOPHARMACOLOGY. 2016;26:S742-S3.

231. Hirata FC, Lima MCO, Sales de Bruin VM, Norega PR, Wenceslau GP, Carvalhedo de Bruin PF, et al. Depression in medical school: The influence of morningness-eveningness. CHRONOBIOLOGY INTERNATIONAL. 2007;24(5):939-46.

232. Hodoba D. Chewing can relieve sleepiness in a night of sleep deprivation. United States1999 1999. 101-5 p.

233. Hosen I, Al Mamun F, Mamun MA. The role of sociodemographics, behavioral factors, and internet use behaviors in students' psychological health amid COVID-19 pandemic in Bangladesh. Health science reports. 2021;4(4):e398.

234. Hossain S, Anjum A, Uddin ME, Rahman MA, Hossain MF, Hossain Sahadat, et al. Impacts of socio-cultural environment and lifestyle factors on the psychological health of university students in Bangladesh: A longitudinal study. JOURNAL OF AFFECTIVE DISORDERS. 2019;256:393-403.

235. Huda MN, Billah M, Sharmin S, Amanullah ASM, Hossin MZ, Huda Md. Nazmul, et al. Associations between family social circumstances and psychological distress among the university students of Bangladesh: To what extent do the lifestyle factors mediate? BMC PSYCHOLOGY. 2021;9(1).

236. Huen LLE, Chan TWG, Yu WMM, Wing YK, Huen Lok-Lam E, Chan Tze-Wai G, et al. Do medical students in Hong Kong have enough sleep? SLEEP AND BIOLOGICAL RHYTHMS. 2007;5(3):226-30.

237. Hussain A, Farrukh H, Gulzar A, Naheed K, Mishap HF, Arif Z, et al. Ocular, Medical and Psychological Effects in Medical Students who use Smart Phones. PAKISTAN JOURNAL OF MEDICAL & HEALTH SCIENCES. 2021;15(2):443-5.

238. Ilic I, Milicic V, Grujicic S, Zivanovic Macuzic I, Kocic S, Ilic MD. Prevalence and correlates of low back pain among undergraduate medical students in Serbia, a cross-sectional study. PeerJ. 2021;9:e11055.

239. Jahrami H, Alshomili H, Almannai N, Althani N, Aloffi A, Algahtani H, et al. Predictors of Excessive Daytime Sleepiness in Medical Students: A Meta-Regression. Clocks & sleep. 2019;1(2):209-19.

240. Jakubiec D, Kornafel D, Cygan A, Górska-Kłęk L, Chromik K. Lifestyle of students from different universities in Wroclaw, Poland. Roczniki Panstwowego Zakladu Higieny. 2015;66(4):337-44.

241. Janatmakan Amiri A, Morovatdar N, Soltanifar A, Rezaee R. Prevalence of Sleep Disturbance and Potential Associated Factors among Medical Students from Mashhad, Iran. Sleep disorders. 2020;2020:4603830.

242. Johnson H, Guhl G, Arora J, Walling A. Migraine in students of a US medical school. Family medicine. 2014;46(8):615-9.

243. K HG, Rao Vanamali D, Baa J. Smartphone Use 'More than Intention': Is it Detrimental for Sleep and Behaviour of Medical Students? The Journal of the Association of Physicians of India. 2020;68(4):80-1.

244. Kanikowska D, Sikorska D, Kuczyńska B, Grzymisławski M, Bręborowicz A, Witowski J. Do medical students adhere to advice regarding a healthy lifestyle? A pilot study of BMI and some aspects of lifestyle in medical students in Poland. Advances in clinical and experimental medicine : official organ Wroclaw Medical University. 2017;26(9):1391-8.

245. Lane A, McGrath J, Cleary E, Guerandel A, Malone KM. Worried, weary and worn out: mixed-method study of stress and well-being in final-year medical students. BMJ open. 2020;10(12):e040245.

246. Lawson HJ, Wellens-Mensah JT, Attah Nantogma S. Evaluation of Sleep Patterns and Self-Reported Academic Performance among Medical Students at the University of Ghana School of Medicine and Dentistry. Sleep disorders. 2019;2019:1278579.

247. Lehnkering H, Siegmund R. Influence of chronotype, season, and sex of subject on sleep behavior of young adults. Chronobiology international. 2007;24(5):875-88.

248. Lehnkering H, Strauss A, Wegner B, Siegmund R. Actigraphic investigations on the activity-rest behavior of right- and left-handed students. England2006 2006. 593-605 p.

249. Luciano F, Cenacchi V, Vegro V, Pavei G. COVID-19 lockdown: Physical activity, sedentary behaviour and sleep in Italian medicine students. European journal of sport science. 2021;21(10):1459-68.

250. Marcel FD. Enabling and encouraging sleep deprivation among medical students2020 2020-3. e1-e4 p.

251. Marques DR, Allen Gomes AC, Drake CL, Roth T, de Azevedo MHP. Assessing Stress-Induced Sleep Reactivity in College Students: The European Portuguese Version of the Ford Insomnia Response to Stress Test (FIRST). Behavioral sleep medicine. 2018;16(4):337-46.

252. Marques DR, Gomes AA, de Azevedo MHP. Profiles of Subjective Daytime Sleepiness through Cluster Analysis. The Psychiatric quarterly. 2020;91(1):147-63.

253. Marques DR, Gomes AA, Di Milia L, Azevedo MH. Circadian preferences in young adults: Psychometric properties and factor structure of the Portuguese version of the Preferences Scale (PS-6). Chronobiology international. 2017;34(3):403-10.

254. Marques DR, Meia-Via AMS, da Silva CF, Gomes AA. Associations between sleep quality and domains of quality of life in a non-clinical sample: results from higher education students. Sleep health. 2017;3(5):348-56.

255. Mazar D, Gileles-Hillel A, Reiter J. Sleep education improves knowledge but not sleep quality among medical students. Journal of clinical sleep medicine : JCSM : official publication of the American Academy of Sleep Medicine. 2021;17(6):1211-5.

256. Miguel AQC, Tempski P, Kobayasi R, Mayer FB, Martins MA. Predictive factors of quality of life among medical students: results from a multicentric study. BMC psychology. 2021;9(1):36.

257. Milojevich HM, Lukowski AF. Sleep and Mental Health in Undergraduate Students with Generally Healthy Sleep Habits. PloS one. 2016;11(6):e0156372.

258. Mirghani HO. The effect of chronotype (morningness/eveningness) on medical students' academic achievement in Sudan. Journal of Taibah University Medical Sciences. 2017;12(6):512-6.

259. Mirghani HO, Albalawi KS, Alali OY, Albalawi WM, Albalawi KM, Aljohani TR, et al. Breakfast skipping, late dinner intake and chronotype (eveningness-morningness) among medical students in Tabuk City, Saudi Arabia. The Pan African medical journal. 2019;34:178.

260. Mirghani HO, Mohammed OS, Almurtadha YM, Ahmed MS. Good sleep quality is associated with better academic performance among Sudanese medical students. BMC research notes. 2015;8:706.

261. Mok D, Bednarz J Jr, Zieren J, Ferguson T, Glass J, Smith K, et al. Double the Prevalence of Stage 2 Hypertension Readings in a Small Group of American Pre-clinical Medical Students Compared to Young Adults Diagnosed with Stage 2 Hypertension in the United States. Cureus. 2020;12(3):e7448.

262. Nechita F, Nechita D, Pîrlog MC, Rogoveanu I. Stress in medical students. Romanian journal of morphology and embryology = Revue roumaine de morphologie et embryologie. 2014;55(3):1263-6.

263. Nepal DB, Kumar T, Mandal MB, Deshpande SB. Sleep duration of an individual has adverse influence on auditory episodic memory. Indian journal of physiology and pharmacology. 2007;51(4):326-32.

264. P Bogui, M Keita, C Dah, N Fidier, ML Buguet-Brown, A Buguet. [The sleep of Africans and Europeans in the Ivory Coast: questionnaire study]. Sante (Montrouge, France). 2002;12(2):263-70.

265. P Csépe, E Dinya, P Balázs, SM Hosseini, G Küzdy, L Rosivall. Impact of the first wave of COVID-19 pandemic on the Hungarian university students' social and health behaviour. Zeitschrift fur Gesundheitswissenschaften = Journal of public health. 2021:1-7.

266. P Giri, M Baviskar, D Phalke. Study of sleep habits and sleep problems among medical students of pravara institute of medical sciences loni, Western maharashtra, India. Annals of medical and health sciences research. 2013;3(1):51-4.

267. P Mishra, M Panigrahi, D Ankit. Cognition and Alertness in Medical Students: Effects of a Single Night of Partial Sleep Deprivation. Annals of neurosciences. 2020;27(2):57-62.

268. P Schestatsky, L Dall-Agnol, L Gheller, LC Stefani, PR Sanches, IC de Souza, et al. Pain-autonomic interaction after work-induced sleep restriction. European journal of neurology. 2013;20(4):638-46.

269. P Stalin, SB Abraham, K Kanimozhy, RV Prasad, Z Singh, AJ Purty. Mobile Phone Usage and its Health Effects Among Adults in a Semi-Urban Area of Southern India. Journal of clinical and diagnostic research : JCDR. 2016;10(1):LC14-6.

270. P Tempski, PL Bellodi, HB Paro, SC Enns, MA Martins, LB Schraiber. What do medical students think about their quality of life? A qualitative study. BMC medical education. 2012;12:106.

271. P Xiao, L Chen, X Dong, Z Zhao, J Yu, D Wang, et al. Anxiety, Depression, and Satisfaction With Life Among College Students in China: Nine Months After Initiation of the Outbreak of COVID-19. Frontiers in psychiatry. 2021;12:777190.

272. Pablo MP, Sandagon MJ, Jorge M, Philina Pablo Ma, Jane Sandagon Mary, Jorge Manuel. CORRELATION BETWEEN EXCESSIVE DAYTIME SLEEPINESS AND THE RISK FOR OBSTRUCTIVE SLEEP APNEA WITH ACADEMIC PERFORMANCE AMONG MEDICAL STUDENTS. RESPIROLOGY. 2010;15:108-.

273. Pagnin D, de Queiroz V. Influence of burnout and sleep difficulties on the quality of life among medical students. SpringerPlus. 2015;4:676.

274. Pagnin D, de Queiroz V, Carvalho YT, Dutra AS, Amaral MB, Queiroz TT. The relation between burnout and sleep disorders in medical students. Academic psychiatry : the journal of the American Association of Directors of Psychiatric Residency Training and the Association for Academic Psychiatry. 2014;38(4):438-44.

275. Pagnin D, De Queiroz V, De Oliveira Filho MA, Gonzalez NV, Salgado AE, Cordeiro e Oliveira B, et al. Burnout and career choice motivation in medical students. Medical teacher. 2013;35(5):388-94.

276. Pandejpong D, Paisansudhi S, Udompunthurak S. Factors associated with consumption of caffeinated-beverage among Siriraj pre-clinical year medical students, A 2-year consecutive survey. Journal of the Medical Association of Thailand = Chotmaihet thangphaet. 2014;97:S189-96.

277. Perez-Dominguez F, Polanco-Ilabaca F, Pinto-Toledo F, Michaeli D, Achiardi J, Santana V, et al. Lifestyle Changes Among Medical Students During COVID-19 Pandemic: A Multicenter Study Across Nine Countries. Health education & behavior : the official publication of the Society for Public Health Education. 2021;48(4):446-54.

278. Pérez-Olmos I, Ibáñez-Pinilla M. Night shifts, sleep deprivation, and attention performance in medical students. International journal of medical education. 2014;5:56-62.

279. Perveen I, Parvin R, Saha M, Bari MS, Huda MN, Ghosh MK. Prevalence of Irritable Bowel Syndrome (IBS), Migraine and Co-Existing IBS-Migraine in Medical Students. Journal of clinical and diagnostic research : JCDR. 2016;10(11):OC09-OC13.

280. Peterson DF, Degenhardt BF, Smith CM. Correlation between prior exercise and present health and fitness status of entering medical students. The Journal of the American Osteopathic Association. 2003;103(8):361-6.

281. Printemps C, Cohen S, Poisson MA, Gibert MH, McCann CC, Quera Salva MA. [Sleep and vigilance in students]. France1999 1999-3. 17-28 p.

282. R Tyssen, P Vaglum, NT Grønvold, Ø Ekeberg. The relative importance of individual and organizational factors for the prevention of job stress during internship: a nationwide and prospective study. Medical teacher. 2005;27(8):726-31.

283. R Wilf-Miron, I Kagan, M Saban. Health behaviors of medical students decline towards residency: how could we maintain and enhance these behaviors throughout their training. Israel journal of health policy research. 2021;10(1):13.

284. Rafeeq S, Naman MS, Ijaz MT, Touseef M, Rai IA, Chaudhry MA, et al. Association of excessive smartphone use with sleep in students of a private medical college in Lahore, Pakistan. RAWAL MEDICAL JOURNAL. 2021;46(4):947-50.

285. Rafiq CA, Ali AA, Ahmad T, Rafiq Ch Ahmer, Ali Ahmad Afnan, Ahmad Taimoor. Effect of Sleep Disturbance on Academic Performance of Students of Public Sector Medical College of Pakistan. INTERNATIONAL JOURNAL OF ADVANCED BIOTECHNOLOGY AND RESEARCH. 2017;8(4):546-+.

286. Rajendran D, Vinod PB, Karthika M, Prathibha MT, Rajendran Deepa, Vinod P. B., et al. EXCESSIVE DAYTIME SLEEPINESS IN MEDICAL STUDENTS. JOURNAL OF EVOLUTION OF MEDICAL AND DENTAL SCIENCES-JEMDS. 2018;7(6):747-9.

287. Ramon-Arbues E, Gea-Caballero V, Granada-Lopez JM, Juarez-Vela R, Pellicer-Garcia B, Anton-Solanas I, et al. The Prevalence of Depression, Anxiety and Stress and Their Associated Factors in College Students. INTERNATIONAL JOURNAL OF ENVIRONMENTAL RESEARCH AND PUBLIC HEALTH. 2020;17(19).

288. Ramos JN, Muraro AP, Nogueira PS, Ferreira MG, Rodrigues PRM, Ramos Juliana Nunes, et al. Poor sleep quality, excessive daytime sleepiness and association with mental health in college students. ANNALS OF HUMAN BIOLOGY. 2021;48(5):382-8.

289. Randjelovic P, Stojiljkovic N, Radulovic N, Ilic I, Stojanovic N, Ilic S, et al. The association of smartphone usage with subjective sleep quality and daytime sleepiness among medical students. BIOLOGICAL RHYTHM RESEARCH. 2019;50(6):857-65.

290. Rangel TC, Raposo MCF, Rocha PAS, Rangel Tathiana Correa, Falcao Raposo Maria Cristina, Sampaio Rocha-Filho Pedro Augusto. The prevalence and severity of insomnia in university students and their associations with migraine, tension-type headache, anxiety and depression disorders: a cross-sectional study. SLEEP MEDICINE. 2021;88:241-6.

291. Raoof AM, Asaad YA, Al-Hadithi TS. Distribution of Chronotypes among a Sample of Iraqi Kurdish Medical Students. Sultan Qaboos University medical journal. 2014;14(3):e356-60.

292. Rathi A, Ransing RS, Mishra KK, Narula N, Rathi Anurag, Ransing Ramdas Sarjerao, et al. Quality of Sleep among Medical Students: Relationship with Personality Traits. JOURNAL OF CLINICAL AND DIAGNOSTIC RESEARCH. 2018;12(9):VC1-VC4.

293. RC Dutra da Silva, A Garcez, MP Pattussi, MTA Olinto. Prevalence and factors associated with excessive and severe daytime sleepiness among healthcare university students in the Brazilian Midwest. Journal of sleep research. 2021:e13524.

294. Rebello CR, Kallingappa PB, Hegde PG. Assessment of perceived stress and association with sleep quality and attributed stressors among 1(st)-year medical students: A cross-sectional study from Karwar, Karnataka, India. Ci ji yi xue za zhi = Tzu-chi medical journal. 2018;30(4):221-6.

295. Rehman R, Zafar A, Mohib A, Hussain M, Ali R, Rehman Rehana, et al. Self-reported academic performance in relation to health behaviours among Bahria University students. JOURNAL OF THE PAKISTAN MEDICAL ASSOCIATION. 2018;68(2):195-9.

296. Rejeb H, Kaddoussi R, Ben Saida I, Ben Khelifa M, Najjar A, Aissa S, et al. Sleep patterns and predictors of poor sleep quality among Tunisian medical students (MS). EUROPEAN RESPIRATORY JOURNAL. 2018;52.

297. Rempel AML, Barlow PB, Kaldjian LC. Medical Education and the Ethics of Self-Care: A Survey of Medical Students Regarding Professional Challenges and Expectations for Living Healthy Lifestyles. Southern medical journal. 2021;114(12):783-8.

298. RF Damiano, IN de Oliveira, ODS Ezequiel, AL Lucchetti, G Lucchetti. The root of the problem: identifying major sources of stress in Brazilian medical students and developing the Medical Student Stress Factor Scale. Revista brasileira de psiquiatria (Sao Paulo, Brazil : 1999). 2020;43(1):35-42.

299. Riemenschneider H, Balázs P, Balogh E, Bartels A, Bergmann A, Cseh K, et al. Do socio-cultural factors influence medical students' health status and health-promoting behaviors? A cross-sectional multicenter study in Germany and Hungary. BMC public health. 2016;16:576.

300. Rizzatti F, Neto OR, Xavier JA, Rizzatti F., Neto O. R., Xavier J. A. Evaluation of the Sleep Quality of Medical Students and Its Relationship with Body Mass Index and Physical Activity Status. AMERICAN JOURNAL OF RESPIRATORY AND CRITICAL CARE MEDICINE. 2019;199.

301. RK Jha, DK Shah, S Basnet, KR Paudel, P Sah, AK Sah, et al. Facebook use and its effects on the life of health science students in a private medical college of Nepal. BMC research notes. 2016;9:378.

302. RL Cook, RJ Noecker, GW Suits. Time allocation of students in basic clinical clerkships in a traditional curriculum. Academic medicine : journal of the Association of American Medical Colleges. 1992;67(4):279-81.

303. RN Rodrigues, CA Viegas, AA Abreu E Silva, P Tavares. Daytime sleepiness and academic performance in medical students. Arquivos de neuro-psiquiatria. 2002;60(1):6-11.

304. Rodriguez M, Ascuntar N, Gonzalez P, Fors M, Rodriguez Montserrat, Ascuntar Nathalia, et al. Excessive daytime somnolence in a sample of Ecuadorian undergraduate medical students and its relationship with academic performance. COGENT EDUCATION. 2021;8(1).

305. Romo-Nava F, Tafoya SA, Gutiérrez-Soriano J, Osorio Y, Carriedo P, Ocampo B, et al. The association between chronotype and perceived academic stress to depression in medical students. Chronobiology international. 2016;33(10):1359-68.

306. Rosenthal DM, Conserve DF, Severe D, Gedeon MA, Zizi F, Casimir G, et al. Sleep Apnea Symptoms and Cardiovascular Disease Risks among Haitian Medical Students. Journal of sleep disorders & therapy. 2017;6(2).

307. Rossener R, Morais IR, Caldeira MF, Hachul H, Rossener Raissa, Morais Isabella Rocha, et al. Assessing sleep among the next generation of healthcare delivery professionals. SLEEP SCIENCE. 2020;13(1):92-3.

308. RS Shah, DS Christian. Association of socio-demographic, dietary and lifestyle factors with Premenstrual Syndrome (PMS) among undergraduate medical students of a tertiary care institute in Ahmedabad, Gujarat. Journal of family medicine and primary care. 2020;9(11):5719-24.

309. Rudinskaitė I, Mačiūtė E, Gudžiūnaitė G, Gerulaitytė G. Burnout Syndrome Amongst Medicine Students in Lithuania and Germany. Acta medica Lituanica. 2020;27(2):53-60.

310. Ruivo Marques D, Allen Gomes A, Nicassio PM, Azevedo MHP. Pre-Sleep Arousal Scale (PSAS): psychometric study of a European Portuguese version. Sleep medicine. 2018;43:60-5.

311. Russo PM, Mattarozzi K, Mazzetti M, Martoni M, Russo P. M., Mattarozzi K., et al. Sleep related problems and academic performance in medical students. JOURNAL OF SLEEP RESEARCH. 2016;25:374-5.

312. Ruzhenkov VA, Ruzhenkova VV, Lukyantseva IS, Boeva AV, Moskvitina US, Ruzhenkov Victor A., et al. Academical Stress for the First and Second Year Medical Students and Possible Risks to Mental Health. INTERNATIONAL JOURNAL OF ADVANCED BIOTECHNOLOGY AND RESEARCH. 2017;8(4):2218-25.

313. S Assaad, C Costanian, G Haddad, F Tannous. Sleep patterns and disorders among university students in Lebanon. Journal of research in health sciences. 2014;14(3):198-204.

314. S Al-Kandari, A Alsalem, S Al-Mutairi, D Al-Lumai, A Dawoud, M Moussa. Association between sleep hygiene awareness and practice with sleep quality among Kuwait University students. Sleep health. 2017;3(5):342-7.

315. S Ball, A Bax. Self-care in medical education: effectiveness of health-habits interventions for first-year medical students. Academic medicine : journal of the Association of American Medical Colleges. 2002;77(9):911-7.

316. S Basu, P Sharma, R Rustagi, R Sharma, N Sharma. Measuring addiction to internet gaming among Indian medical students: Development and preliminary psychometric properties of a new scale. Industrial psychiatry journal. 2020;29(1):33-7.

317. S Basu, R Sharma, P Sharma, N Sharma. Addiction-like behavior associated with social media usage in undergraduate students of a government medical college in Delhi, India. Indian journal of psychiatry. 2021;63(1):35-40.

318. Saguem BN, Nakhli J, Romdhane I, Nasr SB. Predictors of sleep quality in medical students during COVID-19 confinement. L'Encephale. 2022;48(1):3-12.

319. Saraswathi I, Saikarthik J, Senthil Kumar K, Madhan Srinivasan K, Ardhanaari M, Gunapriya R. Impact of COVID-19 outbreak on the mental health status of undergraduate medical students in a COVID-19 treating medical college: a prospective longitudinal study. PeerJ. 2020;8:e10164.

320. SB Goldin, MM Wahi, OS Farooq, HA Borgman, HL Carpenter, LR Wiegand, et al. Student quality-of-life declines during third year surgical clerkship. The Journal of surgical research. 2007;143(1):151-7.

321. SB Karia, N Mehta, D Harshe, A De Sousa, N Shah. Insomnia, dreams, and suicide: Connecting links. Industrial psychiatry journal. 2016;25(2):155-9.

322. Sengupta D, Gupta S, Mukherjee SS, Das S, Sengupta Debalina, Gupta Sumana, et al. Relationship between Sleep Quality and Internet Addiction among First Year Medical Students in a Government Medical College in West Bengal: A Cross-sectional Study. JOURNAL OF CLINICAL AND DIAGNOSTIC RESEARCH. 2021;15(10):LC32-LC5.

323. Seo MW, Lim ES, Park SH, Seo MW, Lim ES, Park SH. Sleep deprivation headache in medical students. CEPHALALGIA. 2005;25(10):960-1.

324. SH Kathem, AA Al-Jumail, M Noor-Aldeen, N Najah, DA Khalid. Measuring depression and anxiety prevalence among Iraqi healthcare college students using hospital anxiety and depression scale. Pharmacy practice. 2021;19(2):2303.

325. Shafique Z, Syed F, Naz S, Urooj S, Khan S, Javed S, et al. Assessment of factors affecting the sleep hygiene of medical students in Bahawalpur, Pakistan: a cross-sectional study. SLEEP SCIENCE. 2021;14(3):273-9.

326. Shaik L, Singh R, Devara J, Basa P, Shah K, Shaik L., et al. Psychiatric impact of mobile usage on medical student life: Ringxiety, nomophobia, and sleep. EUROPEAN PSYCHIATRY. 2021;64:S395-S.

327. Shaikh BT, Kahloon A, Kazmi M, Khalid H, Nawaz K, Khan N, et al. Students, stress and coping strategies: a case of Pakistani medical school. Education for health (Abingdon, England). 2004;17(3):346-53.

328. Shapiro CM, Press P, Weiss R. Sleep behavior and examination results of medical students. Journal of medical education. 1980;55(11):960-2.

329. Shatla MM, Alotaibi NT, Munshi SA, Yaseen EE, Qanadeely EM, Alshaddadi WA, et al. Covid-19 and its impact on education, social life, and mental health among medical students in Saudi Arabia. MEDICAL SCIENCE. 2021;25(116):2708-17.

330. Shehata SF, Al-Malki AQ, Alqahtani AJ, Tamraa AAA, Almutlaq AH, Alshamrani AS, et al. Prevalence of primary headache among King Khalid University students in 2019. WORLD FAMILY MEDICINE. 2020;18(10):57-65.

331. Shehata SF, Alshahrani MS, Aldarami MS, Asiri FA, Alghamdi HA, Shehata Shehata Farag, et al. Prevalence and association between sleep, stress, and physical activity among medical students in southern region, Saudi Arabia. WORLD FAMILY MEDICINE. 2020;18(11):93-101.

332. Shiba S, Vivek N, Chandrika P, Shiba S., Vivek N., Chandrika Poorna. INTERNET ADDICTION AND ITS ASSOCIATION WITH PSYCHOPATHOLOGY AND QUALITY OF SLEEP AMONG MEDICAL STUDENTS. INDIAN JOURNAL OF PSYCHIATRY. 2019;61(9):S504-S.

333. Shigihara Y, Tanaka M, Watanabe Y, Shigihara Yoshihito, Tanaka Masaaki, Watanabe Yasuyoshi. Relationship Between Fatigue and Photosensitivity. BEHAVIORAL MEDICINE. 2010;36(4):109-12.

334. Shrestha D, Adhikari SP, Rawal N, Budhathoki P, Pokharel S, Adhikari Y, et al. Sleep quality among undergraduate students of a medical college in Nepal during COVID-19 pandemic: an online survey. F1000Research. 2021;10:505.

335. Singh HK, Joshi A, Malepati RN, Najeeb S, Balakrishna P, Pannerselvam NK, et al. A survey of E-learning methods in nursing and medical education during COVID-19 pandemic in India. Nurse education today. 2021;99:104796.

336. Sitticharoon C, Srisuma S, Kanavitoon S, Summachiwakij S. Exploratory study of factors related to educational scores of first preclinical year medical students. United States2014 2014-3. 25-33 p.

337. Suarez DE, Cardozo AC, Ellmer D, Trujillo EM. Short report: cross sectional comparison of anxiety and depression symptoms in medical students and the general population in Colombia. England2021 2021-3. 375-80 p.

338. Suárez DE, Cardozo AC, Villarreal ME, Trujillo EM. Non-Heterosexual Medical Students Are Critically Vulnerable to Mental Health Risks: The Need to Account for Sexual Diversity in Wellness Initiatives. Teaching and learning in medicine. 2021;33(1):1-9.

339. Taylor DJ, Vatthauer KE, Bramoweth AD, Ruggero C, Roane B. The role of sleep in predicting college academic performance: is it a unique predictor? Behavioral sleep medicine. 2013;11(3):159-72.

340. Torun F, Torun SD. The psychological impact of the COVID-19 pandemic on medical students in Turkey. Pakistan journal of medical sciences. 2020;36(6):1355-9.

341. Tsurgan AM, Dementiev AA, Lyapkalo AA, Solovyov DA, Kharitonov VI, Stuneeva GI, et al. [The characteristics of life-style of medical students]. Problemy sotsial'noi gigieny, zdravookhraneniia i istorii meditsiny. 2021;29(6):1531-5.

342. Uçar C, Özgöçer T, Yildiz S. Late-night exercise affects the autonomic nervous system activity but not the hypothalamo-pituitary-adrenal axis in the next morning. The Journal of sports medicine and physical fitness. 2018;58(1):57-65.

343. Younes F, Halawi G, Jabbour H, El Osta N, Karam L, Hajj A, et al. Internet Addiction and Relationships with Insomnia, Anxiety, Depression, Stress and Self-Esteem in University Students: A Cross-Sectional Designed Study. PloS one. 2016;11(9):e0161126.

344. Zhang C, Wang T, Zeng P, Zhao M, Zhang G, Zhai S, et al. Reliability, Validity, and Measurement Invariance of the General Anxiety Disorder Scale Among Chinese Medical University Students. Frontiers in psychiatry. 2021;12:648755.

345. Zhang C, Zeng P, Tan J, Sun S, Zhao M, Cui J, et al. Relationship of Problematic Smartphone Use, Sleep Quality, and Daytime Fatigue Among Quarantined Medical Students During the COVID-19 Pandemic. Frontiers in psychiatry. 2021;12:755059.

346. Zhang H, Gao H, Zhu Y, Zhu Y, Dang W, Wei R, et al. Relationship Between Myopia and Other Risk Factors With Anxiety and Depression Among Chinese University Freshmen During the COVID-19 Pandemic. Frontiers in public health. 2021;9:774237.

347. A Mishra, G Banwari, P Yadav. Premenstrual dysphoric disorder in medical students residing in hostel and its association with lifestyle factors. Industrial psychiatry journal. 2015;24(2):150-7.

348. Abdel Rahman AG, Al Hashim BN, Al Hiji NK, Al-Abbad Z. Stress among medical Saudi students at College of Medicine, King Faisal University. Journal of preventive medicine and hygiene. 2013;54(4):195-9.

349. Abuduxike G, Asut O, Abuduxike Gulifeiya, Asut Ozen. Assessment of the Healthy Lifestyle Behaviors and Associated Factors among First-Year Medical Students in Northern Cyprus. CYPRUS JOURNAL OF MEDICAL SCIENCES. 2021;6(3):192-200.

350. Akram A, Akram A. Sleep disorders among medical students of Bolan medical college Quetta, Pakistan. JOURNAL OF SLEEP RESEARCH. 2016;25:340-.

351. Al-Towairqi SA, Alharthi WJ, Almalki AS, Dabi MM, Althobaiti RA, Al-Towairqi Sameer A, et al. Prevalence and risk factors of gastroesophageal reflux disease among female Medical students at Taif University, Saudi Arabia. WORLD FAMILY MEDICINE. 2020;18(12):77-81.

352. Alam MD, Lu J, Ni L, Hu SH, Xu Y, Alam Md Dhedharul, et al. Psychological Outcomes and Associated Factors Among the International Students Living in China During the COVID-19 Pandemic. FRONTIERS IN PSYCHIATRY. 2021;12.

353. Alammar AM, Al Saleem SA, Al-Garni AM, Alalammar RS, alhumayed RS, Alammar Afnan Mastour, et al. Impact of Using Social Media on Mental Health among University Medical Students in Abha City, Southern Saudi Arabia. WORLD FAMILY MEDICINE. 2021;19(1):40-7.

354. Albaker AB, Abdalla SM, Almuhaydib AS, Alobaid OA, Alanazi FM, Alfadhel TO, et al. Impact of Video Games on Physical and Psychological Wellbeing Among Medical Students. JOURNAL OF RESEARCH IN MEDICAL AND DENTAL SCIENCE. 2021;9(5):327-33.

355. Aldhawyan AF, Alfaraj AA, Elyahia SA, Alshehri SZ, Alghamdi AA. Determinants of Subjective Poor Sleep Quality in Social Media Users Among Freshman College Students. Nature and science of sleep. 2020;12:279-88.

356. Algarni ASA, Alqahtani WSS, Alotaibi FSA, Asiri MAM, Al Hoban MAM, Alshehri ZKS, et al. GENDER DIFFERENCES IN HABITS FOR A HEALTHY LIFESTYLE AMONG MEDICAL STUDENTS, SAUDI ARABIA. INDO AMERICAN JOURNAL OF PHARMACEUTICAL SCIENCES. 2019;6(1):636-42.

357. Algarni SA, Aljohani AS, Algarni Sawsan Abdullah, Aljohani Abdulaziz Saleh. Effect of smartphone addiction on sleep quality among medical students at Taibah University, Medina, Saudi Arabia. MEDICAL SCIENCE. 2021;25(118):3266-78.

358. Alghadir A, Manzar MD, Anwer S, Albougami A, Salahuddin M, Alghadir Ahmad, et al. Psychometric Properties of the Generalized Anxiety Disorder Scale Among Saudi University Male Students. NEUROPSYCHIATRIC DISEASE AND TREATMENT. 2020;16:1427-32.

359. Alharbi MF, Alharbi RM, Alshweash M, Alharbi F, Almetrafe A, Alfozan F, et al. The role of lifestyle habits in the prevalence of overweight and obesity among students. AMAZONIA INVESTIGA. 2021;10(47):250-62.

360. Ali A, Albahrani A, Alnasser A, Alsalman A, Alaithan M, Alswaidan M, et al. The Effect of caffeine on sleep among medical students at King Faisal University Saudi Arabia. WORLD FAMILY MEDICINE. 2021;19(6):51-8.

361. Almojali AI, Almalki SA, Alothman AS, Masuadi EM, Alaqeel MK. The prevalence and association of stress with sleep quality among medical students. Journal of epidemiology and global health. 2017;7(3):169-74.

362. Alodhayani AA, Alshaikh OM, Ghomraoui FA, AlShaibani TJ, Algadheeb AS, Bendahmash AW, et al. Correlation between obesity and sleep disturbance in Saudi medical students. Journal of physical therapy science. 2017;29(2):181-6.

363. Alotaibi AD, Alosaimi FM, Alajlan AA, Bin Abdulrahman KA. The relationship between sleep quality, stress, and academic performance among medical students. Journal of family & community medicine. 2020;27(1):23-8.

364. Asghar AA, Faiq A, Shafique S, Siddiqui F, Asghar N, Malik S, et al. Prevalence and Predictors of the Burnout Syndrome in Medical Students of Karachi, Pakistan. Cureus. 2019;11(6):e4879.

365. Awasthi AA, Taneja N, Maheshwari S, Gupta T, Bhavika. Prevalence of Internet Addiction, Poor Sleep Quality, and Depressive Symptoms Among Medical Students: A Cross-Sectional Study. Osong public health and research perspectives. 2020;11(5):303-8.

366. Delgado ÁHA, Almeida JPR, Mendes LSB, Oliveira IN, Ezequiel ODS, Lucchetti ALG, et al. Are surface and deep learning approaches associated with study patterns and choices among medical students? A cross-sectional study. Sao Paulo medical journal = Revista paulista de medicina. 2018;136(5):414-20.

367. Dixit A, Thawani R, Goyal A, Vaney N. Psychomotor performance of medical students: effect of 24 hours of sleep deprivation. Indian journal of psychological medicine. 2012;34(2):129-32.

368. Flindall IR, Leff DR, Pucks N, Sugden C, Darzi A. The Preservation of Cued Recall in the Acute Mentally Fatigued State: A Randomised Crossover Study. World journal of surgery. 2016;40(1):56-65.

369. Iorga M, Muraru ID, Munteanu C, Petrariu FD, Iorga Magdalena, Muraru Iulia-Diana, et al. DEPRESSION, ANXIETY AND STRESS AMONG MEDICAL STUDENTS. MEDICAL-SURGICAL JOURNAL-REVISTA MEDICO-CHIRURGICALA. 2019;123(3):496-505.

370. Mandal A, Ghosh A, Sengupta G, Bera T, Das N, Mukherjee S. Factors affecting the performance of undergraduate medical students: a perspective. Indian journal of community medicine : official publication of Indian Association of Preventive & Social Medicine. 2012;37(2):126-9.

371. Mboya IB, John B, Kibopile ES, Mhando L, George J, Ngocho JS. Factors associated with mental distress among undergraduate students in northern Tanzania. BMC psychiatry. 2020;20(1):28.

372. Mengi A, Singh A, Gupta V. An institution-based study to assess the prevalence of Nomophobia and its related impact among medical students in Southern Haryana, India. Journal of family medicine and primary care. 2020;9(5):2303-8.

373. Micu A, Cojocaru C, Luca G, Mihăescu T. [Quality of sleep in students]. Romania2012 2012-1. 25-7 p.

374. Mohammadbeigi A, Absari R, Valizadeh F, Saadati M, Sharifimoghadam S, Ahmadi A, et al. Sleep Quality in Medical Students; the Impact of Over-Use of Mobile Cell-Phone and Social Networks. Journal of research in health sciences. 2016;16(1):46-50.

375. Molla A, Wondie T. Magnitude of Poor Sleep Hygiene Practice and Associated Factors among Medical Students in Ethiopia: A Cross-Sectional Study. Sleep disorders. 2021;2021:6611338.

376. Nadeem A, Cheema MK, Naseer M, Javed H. Comparison of quality of sleep between medical and non-medical undergraduate Pakistani students. Pakistan2018 2018-10. 1465-70 p.

377. Nakhostin-Ansari A, Sherafati A, Aghajani F, Khonji MS, Aghajani R, Shahmansouri N. Depression and Anxiety among Iranian Medical Students during COVID-19 Pandemic. Iranian journal of psychiatry. 2020;15(3):228-35.

378. Nteveros A, Kyprianou M, Artemiadis A, Charalampous A, Christoforaki K, Cheilidis S, et al. Burnout among medical students in Cyprus: A cross-sectional study. PloS one. 2020;15(11):e0241335.

379. Paiva T, Goncalves E, Paiva T., Goncalves E. Sleepiness in Portuguese medical students. JOURNAL OF SLEEP RESEARCH. 2006;15:134-.

380. Panigrahi A, Behera BK, Sarma NN, Panigrahi Ansuman, Behera Basanta Kumar, Sarma Nibir Nath. Prevalence, pattern, and associated psychosocial factors of headache among undergraduate students of health profession. CLINICAL EPIDEMIOLOGY AND GLOBAL HEALTH. 2020;8(2):365-70.

381. Patel A, Al-Saffar A, Sharma M, Masiak A, Zdrojewski Z. Prevalence of fibromyalgia in medical students and its association with lifestyle factors - a cross-sectional study. Reumatologia. 2021;59(3):138-45.

382. Pathak R, Aye AM, Pathak A, Pathak R., Aye A. M., Pathak A. Study of stress among first and second year medical and dental students. RESEARCH JOURNAL OF PHARMACEUTICAL BIOLOGICAL AND CHEMICAL SCIENCES. 2017;8(2):2679-84.

383. Patil A, Bhavya, Chaudhury S, Srivastava S. Eyeing computer vision syndrome: Awareness, knowledge, and its impact on sleep quality among medical students. Industrial psychiatry journal. 2019;28(1):68-74.

384. Pavan IS, deFreitas BP, Bittencourt LA, Kobbaz TM, Marcelino LD, Fernandes BD, et al. Reduced hours, snore and low sleep quality as cardiovascular risk factors - Cross-study in medical student. JOURNAL OF CLINICAL HYPERTENSION. 2020;22(4):725-.

385. Peltzer K, Pengpid S, Peltzer Karl, Pengpid Supa. Nocturnal sleep problems among university students from 26 countries. SLEEP AND BREATHING. 2015;19(2):499-508.

386. Pereira-Morales AJ, Adan A, Bussi IL, Camargo A, Pereira-Morales Angela J., Adan Ana, et al. Anxiety symptomatology, sex and chronotype: The mediational effect of diurnal sleepiness. CHRONOBIOLOGY INTERNATIONAL. 2018;35(10):1354-64.

387. Pereira-Morales AJ, Camargo A. Psychological distress among undergraduate medical students: the influence of excessive daytime sleepiness and family functioning. Psychology, health & medicine. 2019;24(8):936-50.

388. PF Lima, AL Medeiros, JF Araujo. Sleep-wake pattern of medical students: early versus late class starting time. Brazil2002 2002-11. 1373-7 p.

389. Piro RS, Alhakem SSM, Azzez SS, Abdulah DM, Piro Rasoul S., Alhakem Salim S. Miho, et al. Prevalence of sleep disorders and their impact on academic performance in medical students/University of Duhok. SLEEP AND BIOLOGICAL RHYTHMS. 2018;16(1):125-32.

390. PM Niemi, PT Vainiomäki. Medical students' distress--quality, continuity and gender differences during a six-year medical programme. Medical teacher. 2006;28(2):136-41.

391. Pop LM, Iorga M, Muraru ID, Petrariu FD, Pop Lavinia-Maria, Iorga Magdalena, et al. Assessment of Dietary Habits, Physical Activity and Lifestyle in Medical University Students. SUSTAINABILITY. 2021;13(6).

392. Potapova EA, Zemlyanoy DA, Kondratyev GV, Potapova Elena A., Zemlyanoy Dmytry A., Kondratyev Gleb, V. Features of Life and Well-Being in Medical Students During Distance Learning in the Course of the COVID-19 Epidemic. PSIKHOLOGICHESKAYA NAUKA I OBRAZOVANIE-PSYCHOLOGICAL SCIENCE AND EDUCATION. 2021;26(3):70-81.

393. PP Chang, DE Ford, LA Mead, L Cooper-Patrick, MJ Klag. Insomnia in young men and subsequent depression. The Johns Hopkins Precursors Study. American journal of epidemiology. 1997;146(2):105-14.

394. Q Ma, X Zhang, L Zou. The Mediating Effect of Alexithymia on the Relationship Between Schizotypal Traits and Sleep Problems Among College Students. Frontiers in psychiatry. 2020;11:153.

395. QL Li, JY Zhao, J Tian, T Sun, CX Zhao, HC Guo, et al. The Association Among Achievement Goal Orientations, Academic Performance, and Academic Well-Being Among Chinese Medical Students: A Cross-Sectional Study. Frontiers in psychology. 2021;12:694019.

396. R Ali, N Mohammed, H Aly. Internet addiction among medical students of Sohag University, Egypt. The Journal of the Egyptian Public Health Association. 2017;92(2):86-95.

397. R Abdelmoaty Goweda, A Hassan-Hussein, M Ali Alqahtani, MM Janaini, AH Alzahrani, BM Sindy, et al. Prevalence of sleep disorders among medical students of Umm Al-Qura University, Makkah, Kingdom of Saudi Arabia. Journal of public health research. 2020;9:2020.

398. R Czabak-Garbacz, A Skibniewska, P Mazurkiewicz, A Wisowska. [Hygiene during leisure time among third year students from the Department of Nursing and Health Sciences]. Poland2002 2002. 203-11 p.

399. R Dragun, NN Veček, M Marendić, A Pribisalić, G Đivić, H Cena, et al. Have Lifestyle Habits and Psychological Well-Being Changed among Adolescents and Medical Students Due to COVID-19 Lockdown in Croatia? Nutrients. 2020;13(1).

400. R Deutsch, Z Ehsan. Sleepless in a pandemic: a medical student's perspective. Journal of clinical sleep medicine : JCSM : official publication of the American Academy of Sleep Medicine. 2021;17(4):867-8.

401. R Javaid, AU Momina, MZ Sarwar, SA Naqi. Quality of Sleep and Academic Performance among Medical University Students. Journal of the College of Physicians and Surgeons--Pakistan : JCPSP. 2020;30(8):844-8.

402. R Jalali, H Khazaei, BK Paveh, Z Hayrani, L Menati. The Effect of Sleep Quality on Students' Academic Achievement. Advances in medical education and practice. 2020;11:497-502.

403. R Jacobs, M Lanspa, M Kane, J Caballero. Predictors of emotional wellbeing in osteopathic medical students in a COVID-19 world. Journal of osteopathic medicine. 2021;121(5):455-61.

404. R Kawyannejad, M Mirzaei, A Valinejadi, B Hemmatpour, HA Karimpour, J AminiSaman, et al. General health of students of medical sciences and its relation to sleep quality, cell phone overuse, social networks and internet addiction. BioPsychoSocial medicine. 2019;13:12.

405. R Khan, R Rehman, M Baig, M Hussain, M Khan, F Syed. Dimensions of physical wellness among medical students of public and private medical colleges in Pakistan. Saudi medical journal. 2015;36(6):754-8.

406. R Mazurkiewicz, D Korenstein, R Fallar, J Ripp. The prevalence and correlations of medical student burnout in the pre-clinical years: a cross-sectional study. Psychology, health & medicine. 2012;17(2):188-95.

407. R Pecotić, M Valić, G Kardum, V Sevo, Z Dogas. [Sleep habits of medical students, physicians and nurses regarding age, sex, shift work and caffein consumption]. Croatia2008 2008-3. 87-91 p.

408. R Shao, P He, B Ling, L Tan, L Xu, Y Hou, et al. Prevalence of depression and anxiety and correlations between depression, anxiety, family functioning, social support and coping styles among Chinese medical students. BMC psychology. 2020;8(1):38.

409. R Singh, R Shriyan, R Sharma, S Das. Pilot Study to Assess the Quality of Life, Sleepiness and Mood Disorders among First Year Undergraduate Students of Medical, Engineering and Arts. Journal of clinical and diagnostic research : JCDR. 2016;10(5):JC01-5.

410. R Shad, R Thawani, A Goel. Burnout and Sleep Quality: A Cross-Sectional Questionnaire-Based Study of Medical and Non-Medical Students in India. Cureus. 2015;7(10):e361.

411. Redig AJ. In search of sleep. Academic medicine : journal of the Association of American Medical Colleges. 2009;84(5):565.

412. Rizvi AH, Awaiz M, Ghanghro Z, Jafferi MA, Aziz S. Pre-examination stress in second year medical students in a government college. Journal of Ayub Medical College, Abbottabad : JAMC. 2010;22(2):152-5.

413. S Bogati, T Singh, S Paudel, B Adhikari, D Baral. Association of the Pattern and Quality of Sleep with Consumption of Stimulant Beverages, Cigarette and Alcohol among Medical Students. Journal of Nepal Health Research Council. 2020;18(3):379-85.

414. S C, D Braganza, N Edwin. Quality of life among interns at a southern Indian tertiary care hospital: a cohort study. The National medical journal of India. 2014;27(4):214-6.

415. S Chatterjee, SK Kar. Smartphone Addiction and Quality of Sleep among Indian Medical Students. Psychiatry. 2021;84(2):182-91.

416. S Dhamija, B Shailaja, B Chaudhari, S Chaudhury, D Saldanha. Prevalence of smartphone addiction and its relation with sleep disturbance and low self- esteem among medical college students. Industrial psychiatry journal. 2021;30:S189-S94.

417. S Fulda. [Diaries of sleep behavior]. Austria1995 1995. 476-7 p.

418. S Gupta, A Bhardwaj, A Nadda, A Gill, A Mittal, S Gupta. A comparative study of sleep quality in different phases of the medical course: A study from Haryana (North India). Journal of family medicine and primary care. 2020;9(4):2006-10.

419. S García-Ptacek, D García Azorín, R Sanchez Salmador, ML Cuadrado, J Porta-Etessam. Hallucinations and aberrant perceptions are prevalent among the young healthy adult population. Neurologia (Barcelona, Spain). 2013;28(1):19-23.

420. S Imani-Masouleh, VK Mandagere, CD Irwin, RG Patel. Medics Sleep: Improving sleep education in medical training. The clinical teacher. 2021;18(4):354-5.

421. S Kaye, J Pathman, JA Skelton. Development and Implementation of a Student-Led Lifestyle Medicine Curriculum. American journal of lifestyle medicine. 2019;13(3):253-61.

422. S Montagnese, L Zarantonello, C Formentin, C Zancato, MB Bonetto, A Biscontin, et al. Sleep, Circadian Rhythmicity and Response to Chronotherapy in University Students: Tips from Chronobiology Practicals. Journal of circadian rhythms. 2021;19:1.

423. S Qanash, F Al-Husayni, H Falata, O Halawani, E Jahra, B Murshed, et al. Effect of Electronic Device Addiction on Sleep Quality and Academic Performance Among Health Care Students: Cross-sectional Study. JMIR medical education. 2021;7(4):e25662.

424. S Romiszewski, FEK May, EJ Homan, B Norris, MA Miller, A Zeman. Medical student education in sleep and its disorders is still meagre 20 years on: A cross-sectional survey of UK undergraduate medical education. Journal of sleep research. 2020;29(6):e12980.

425. S Steinemann, J Omori. Use of a personal digital assistant to monitor surgery student work and sleep hours. American journal of surgery. 2006;191(2):272-5.

426. S Salih, M Fageehi, S Hakami, E Ateya, M Hakami, H Hakami, et al. Academic Difficulties Among Medical Students at Jazan University: A Case-Control Study. Advances in medical education and practice. 2021;12:723-9.

427. S Seger, NNB Nasharuddin, SL Fernandez, SRBM Yunus, NTM Shun, P Agarwal, et al. Prevalence and factors associated with irritable bowel syndrome among medical students in a Malaysian private university: a cross sectional study. The Pan African medical journal. 2020;37:151.

428. S Sharma, PR Srijithesh. Sleeping over a sleep disorder - Awareness of obstructive sleep apnoea as a modifiable risk factor for hypertension and stroke: A survey among health care professionals and medical students. Annals of Indian Academy of Neurology. 2013;16(2):151-3.

429. S Selvaraj, SZ Eusufzai, JA Asif, N Bin Jamayet, WMAW Ahmad, MK Alam. Comparison of knowledge level and attitude towards obstructive sleep apnoea amongst dental and medical undergraduate students of Universiti Sains Malaysia. Work (Reading, Mass). 2021;69(1):173-80.

430. S Tebeka, O Huillard, B Pignon, YL Nguyen, C Dubertret, J Mallet. Medical students and the response to COVID-19: Educational preparedness and psychological impact of their involvement in communicating with patients' relatives. L'Encephale. 2021.

431. S Tariq, S Tariq, S Tariq, S Jawed. Perceived stress, severity and sources of stress among female medical students in a private medical college in Pakistan. JPMA The Journal of the Pakistan Medical Association. 2020;70(1):162-7.

432. S Yasir, B Kant, MF Dar. Frequency of dysmenorrhoea, its impact and management strategies adopted by medical students. Journal of Ayub Medical College, Abbottabad : JAMC. 2014;26(3):349-52.

433. S Yogesh, S Abha, S Priyanka. Mobile usage and sleep patterns among medical students. Indian journal of physiology and pharmacology. 2014;58(1):100-3.

434. SA Tafoya, MM Jurado, NJ Yépez, M Fouilloux, MC Lara. [Sleep difficulties and psychological symptoms in medicine students in Mexico]. Argentina2013 2013. 247-51 p.

435. SA Tafoya, V Aldrete-Cortez, S Ortiz, C Fouilloux, F Flores, AM Monterrosas. Resilience, sleep quality and morningness as mediators of vulnerability to depression in medical students with sleep pattern alterations. Chronobiology international. 2019;36(3):381-91.

436. Saat NZM, Hanawi SA, Chan KS, Hanafiah H, Teh SC, Aznan SR, et al. Sleep Quality among University Students: Associations between Demographic Factors and Physical Activity Level. INTERNATIONAL JOURNAL OF PHARMACEUTICAL RESEARCH AND ALLIED SCIENCES. 2020;9(3):57-65.

437. Sabbagh A, Kryger M, Sabbagh A, Kryger M. Sleep and sleepiness in first year medical students. SLEEP. 2004;27:136-.

438. Said AH, Yusof MZ, Mohd FN, Azmi MAMH, Hanapiah HM, Abdullah AW, et al. Poor Sleep Quality among Medical Students in International Islamic University Malaysia (IIUM) and Its Association with Mental Health and other Factors. IIUM MEDICAL JOURNAL MALAYSIA. 2020;19(2):49-57.

439. SANTIBANEZ I, FERNANDEZ GA, SANTIBANEZ I, FERNANDEZ GA. PATTERNS OF WORKING AND EATING IN SLEEP-WAKEFULNESS CYCLES OF MEDICAL-STUDENTS. ARCHIVOS DE BIOLOGIA Y MEDICINA EXPERIMENTALES. 1988;21(2):R331-R.

440. SANTIBANEZ I, FERNANDEZGONI A, SANCHEZ M, MOYA L, ROMERO H, SANTIBANEZ I, et al. NORMAL CIRCADIAN SLEEP WAKEFULNESS BEHAVIORS IN A POPULATION OF MEDICAL-STUDENTS. ARCHIVOS DE BIOLOGIA Y MEDICINA EXPERIMENTALES. 1987;20(2):R248-R.

441. Sathe H, Saraf A, Talapalliwar M, Patil V, Kumar V, Karia S, et al. Excessive Daytime sleepiness and sleep quality in medical students and their association with smartphone and internet addiction: A cross-sectional study. ANNALS OF INDIAN PSYCHIATRY. 2021;5(2):139-43.

442. Segal AB, Bruno S, Forte WC. Immune function in acute stress. Singapore2006 2006-7. 136-40 p.

443. Sharma A, Sharma PK, Puri P. Prevalence and the risk factors of gastro-esophageal reflux disease in medical students. Medical journal, Armed Forces India. 2018;74(3):250-4.

444. Siddiqui AF, Al-Musa H, Al-Amri H, Al-Qahtani A, Al-Shahrani M, Al-Qahtani M. Sleep Patterns and Predictors of Poor Sleep Quality among Medical Students in King Khalid University, Saudi Arabia. The Malaysian journal of medical sciences : MJMS. 2016;23(6):94-102.

445. Surani AA, Zahid S, Surani A, Ali S, Mubeen M, Khan RH. Sleep quality among medical students of Karachi, Pakistan. JPMA The Journal of the Pakistan Medical Association. 2015;65(4):380-2.

446. SZ Abbasi, N Mubeen, T Ayub, MA Khan, Z Abbasi, N Baig. Comparison of stress levels among medical and dental students in the clinical years of training and their coping strategies. JPMA The Journal of the Pakistan Medical Association. 2020;70(6):1006-8.

447. T Eller, A Aluoja, V Vasar, M Veldi. Symptoms of anxiety and depression in Estonian medical students with sleep problems. Depression and anxiety. 2006;23(4):250-6.

448. T Fujikawa, O Tochikubo, N Kura, S Umemura. Factors related to elevated 24-h blood pressure in young adults. Clinical and experimental hypertension (New York, NY : 1993). 2009;31(8):705-12.

449. T Hori, H Tachikawa, T Ishii, N Shimada, T Takemori, A Lebowitz, et al. [An analysis of mental disorders of international students visiting the Mental Health Service at Tsukuba University Health Center]. Seishin shinkeigaku zasshi = Psychiatria et neurologia Japonica. 2012;114(1):3-12.

450. T Kawada. Agreement rates for sleep/wake judgments obtained via accelerometer and sleep diary: a comparison. United States2008 2008-11. 1026-9 p.

451. T Kawada. Sleep, Depression, and Burnout in Medical Students: Risk Assessment. United States2017 2017-10. 682-3 p.

452. T Noor, A Sajjad, A Asma. Frequency, character and predisposing factor of headache among students of medical college of Karachi. JPMA The Journal of the Pakistan Medical Association. 2016;66(2):159-64.

453. T Nishijima, T Kizawa, K Hosokawa, F Endo, Y Kasai, Y Yamashiro, et al. Prevalence of sleep-disordered breathing in Japanese medical students based on type-3 out-of-center sleep test. Sleep medicine. 2018;41:9-14.

454. T Pramanik, MT Sherpa, R Shrestha. Internet addiction in a group of medical students: a cross sectional study. Nepal Medical College journal : NMCJ. 2012;14(1):46-8.

455. T Reang, H Bhattacharjya. A study to assess the emotional disorders with special reference to stress of medical students of agartala government medical college and govinda ballabh pant hospital. Indian journal of community medicine : official publication of Indian Association of Preventive & Social Medicine. 2013;38(4):207-11.

456. T Stecker. Well-being in an academic environment. Medical education. 2004;38(5):465-78.

457. T Tamaki, Y Kaneita, T Ohida, E Yokoyama, Y Osaki, H Kanda, et al. Prevalence of and factors associated with smoking among Japanese medical students. Journal of epidemiology. 2010;20(4):339-45.

458. T Van der Veer, MH Frings-Dresen, JK Sluiter. Health behaviors, care needs and attitudes towards self-prescription: a cross-sectional survey among Dutch medical students. PloS one. 2011;6(11):e28038.

459. TA Bosi Bağcı, A Kanadıkırık, E Somyürek, G Gerçek, HB Tanrıkulu, E Öntaş, et al. Impact of COVID-19 on eating habits, sleeping behaviour and physical activity status of final-year medical students in Ankara, Turkey. Public health nutrition. 2021;24(18):6369-76.

460. Tahir M, Butt MA, Nazir M, Tahir Muhammad, Butt Muhammad Asad, Nazir Mudassar. ANXIETY LEVEL AMONG M.B.B.S STUDENTS DURING EXAMS. INDO AMERICAN JOURNAL OF PHARMACEUTICAL SCIENCES. 2019;6(5):9235-40.

461. Tahir M, Ul Haiy A, Tahir M, Kuraishi RT, Saqib S, Tahir Maliha, et al. Sleeping Habits among Medical Students of King Edward Medical University, Associated Stress and Effects on Academic Performance. ANNALS OF KING EDWARD MEDICAL UNIVERSITY LAHORE PAKISTAN. 2020;26(2):379-83.

462. Takemura T, Kondoh H, Takemura F, Ohnuma S, Suzuki M, Kanbayashi T, et al. A study on subjective sleepiness in medical students by using Epworth Sleepiness Scale. SLEEP. 2007;30:A216-A.

463. Tauseef HM, Siddque HMW, Akhtar MF, Hurera Abu, Tauseef Hafiz Muhammad, Siddque Hafiz Muhammad Waqas, et al. Frequency of Irritable Bowel Syndrome (IBS) and its Risk Factors among MBBS Students of Allama Iqbal Medical College, Lahore. PAKISTAN JOURNAL OF MEDICAL & HEALTH SCIENCES. 2021;15(7):2062-7.

464. Teimouri A, Amra B. Association between Sleep Quality and Gastroesophageal Reflux in Medical Students. Middle East journal of digestive diseases. 2021;13(2):139-44.

465. Telgote SA, Ghogare AS, Khadse V, Karwande SG, Telgote Shilpa Avinash, Ghogare Ajinkya Sureshrao, et al. Smartphone Addiction and its Impact on Insomnia among the Undergraduate Medical Students of a Teaching Hospital of Maharashtra, India-A Cross-sectional study. JOURNAL OF CLINICAL AND DIAGNOSTIC RESEARCH. 2021;15(12):VC1-VC5.

466. Theron C, van Zyl TL, Joubert A, Kleynhans B, van der Walt P, Hattingh MG, et al. Late-night simulation: Opinions of fourth- and fifth-year medical students at the University of the Free State, Bloemfontein, South Africa. AFRICAN JOURNAL OF HEALTH PROFESSIONS EDUCATION. 2021;13(2):123-8.

467. Thomas PC, Sundar B, Thomas Pradeep C., Sundar Bindu. Sleep Quality, Day Time Sleepiness and Academic Performance in First Year Medical Students. JOURNAL OF EVOLUTION OF MEDICAL AND DENTAL SCIENCES-JEMDS. 2019;8(39):2934-8.

468. TJ Arabatzis, J Marsidi, M Ashraf, C Supino, R Smith. Gender Disparities in Symptoms of Depression After Medical School Exams and Student Coping Strategies for Postexam Depression Symptoms. Journal of medical education and curricular development. 2022;9:23821205211055391.

469. TJ Wang, CL Kang, JL Tsai, WT Song, AS Lien. Social media (Facebook) improper use and the influence of sleeping quality in Taiwan's university students. Science progress. 2021;104(2):368504211011878.

470. Tlili MA, Aouicha W, Sahli J, Testouri A, Hamoudi M, Mtiraoui A, et al. Prevalence of burnout among health sciences students and determination of its associated factors. PSYCHOLOGY HEALTH & MEDICINE. 2021;26(2):212-20.

471. TM Kobbaz, LA Bittencourt, BV Pedrosa, BDM Fernandes, LD Marcelino, BP Freitas, et al. The lifestyle of Brazilian medical students: What changed and how it protected their emotional wellbeing during the COVID-19 pandemic. Australian journal of general practice. 2021;50(9):668-72.

472. TM Wolf, GE Kissling. Changes in life-style characteristics, health, and mood of freshman medical students. Journal of medical education. 1984;59(10):806-14.

473. Toubasi AA, Khraisat BR, AbuAnzeh RB, Kalbouneh HM. A cross sectional study: The association between sleeping quality and stress among second and third medical students at the University of Jordan. International journal of psychiatry in medicine. 2021:912174211011287.

474. U Vollmer-Conna, JE Beilharz, E Cvejic, CL Macnamara, M Doherty, Z Steel, et al. The well-being of medical students: A biopsychosocial approach. The Australian and New Zealand journal of psychiatry. 2020;54(10):997-1006.

475. Ucuz İ, Kayhan Tetik B, Öncü EH, Demiralp İ. Effects of mental and physical performance on attention, anger control and sleep quality. Perspectives in psychiatric care. 2019;55(4):632-5.

476. Ukraintsev II, Schastnyy ED, Bokhan NA, Ukraintsev I. I., Schastnyy E. D., Bokhan N. A. Incidence rate and clinical characteristics of seasonal affective disorders in senior medical students. BYULLETEN SIBIRSKOY MEDITSINY. 2021;20(3):112-9.

477. Ul Haq I, Mariyam Z, Li M, Huang X, Jiang P, Zeb F, et al. A Comparative Study of Nutritional Status, Knowledge Attitude and Practices (KAP) and Dietary Intake between International and Chinese Students in Nanjing, China2018 2018-9-3.

478. V Krutsch, A Clement, T Heising, L Achenbach, J Zellner, M Gesslein, et al. Influence of poor preparation and sleep deficit on injury incidence in amateur small field football of both gender. Archives of orthopaedic and trauma surgery. 2020;140(4):457-64.

479. Vgontzas A, Li W, Mostofsky E, Mittleman MA, Bertisch SM. Baseline sleep quality, stress, and depressive symptoms, and subsequent headache occurrence in a six-week prospective cohort study of patients with episodic migraine. Headache. 2021;61(5):727-33.

480. Vujcic I, Stojilovic N, Dubljanin E, Ladjevic N, Ladjevic I, Sipetic-Grujicic S. Low Back Pain among Medical Students in Belgrade (Serbia): A Cross-Sectional Study. Pain research & management. 2018;2018:8317906.

481. Waqas A, Khan S, Sharif W, Khalid U, Ali A. Association of academic stress with sleeping difficulties in medical students of a Pakistani medical school: a cross sectional survey. PeerJ. 2015;3:e840.

482. Weigel A, Hofmeister D, Pröbster K, Brähler E, Gumz A. Eating pathology in medical students in Eastern Germany: comparison with general population and a sample at the time of the German reunification. Eating and weight disorders : EWD. 2016;21(3):445-51.

483. Worobetz A, Retief PJ, Loughran S, Walsh J, Casey M, Hayes P, et al. A feasibility study of an exercise intervention to educate and promote health and well-being among medical students: the 'MED-WELL' programme. BMC medical education. 2020;20(1):183.

484. Y Jeong, JY Kim, JS Ryu, KE Lee, EH Ha, H Park. The Associations between Social Support, Health-Related Behaviors, Socioeconomic Status and Depression in Medical Students. Epidemiology and health. 2010;32:e2010009.

485. Y Koushik, K Hs. Electronic gadget Screen-time, Sleep Quality & Quantity and Academic performance in Medical Students. The Journal of the Association of Physicians of India. 2020;68(1):102.

486. Y Liu, L Liu, Y Yang, Y He, Y Zhang, M Wang, et al. A school-based study of irritable bowel syndrome in medical students in beijing, china: prevalence and some related factors. Gastroenterology research and practice. 2014;2014:124261.

487. Y Mino, N Yasuda, T Fujimura, H Ohara. Caffeine consumption among medical students. Arukoru kenkyu to yakubutsu izon = Japanese journal of alcohol studies & drug dependence. 1990;25(6):475-85.

488. Y Okami, T Kato, G Nin, K Harada, W Aoi, S Wada, et al. Lifestyle and psychological factors related to irritable bowel syndrome in nursing and medical school students. Journal of gastroenterology. 2011;46(12):1403-10.

489. Y Qiu, M Yao, Y Guo, X Zhang, S Zhang, Y Zhang, et al. Health-Related Quality of Life of Medical Students in a Chinese University: A Cross-Sectional Study. International journal of environmental research and public health. 2019;16(24).

490. Y Rong, GM Luscombe, TA Davenport, Y Huang, N Glozier, IB Hickie. Recognition and treatment of depression: a comparison of Australian and Chinese medical students. Germany2009 2009-8. 636-42 p.

491. Y Shen, F Meng, SN Tan, Y Zhang, EC Anderiescu, RE Abeysekera, et al. Excessive daytime sleepiness in medical students of Hunan province: Prevalence, correlates, and its relationship with suicidal behaviors. Journal of affective disorders. 2019;255:90-5.

492. Y Song, K Sznajder, C Cui, Y Yang, Y Li, X Yang. Anxiety and its relationship with sleep disturbance and problematic smartphone use among Chinese medical students during COVID-19 home confinement - A structural equation model analysis. Journal of affective disorders. 2022;296:315-21.

493. Y Shigihara, M Tanaka, Y Watanabe. Relationship between fatigue and photosensitivity. Behavioral medicine (Washington, DC). 2010;36(4):109-12.

494. Y Wang, X Jing, W Han, Y Jing, L Xu. Positive and negative affect of university and college students during COVID-19 outbreak: a network-based survey. International journal of public health. 2020;65(8):1437-43.

495. Y Wang, Y Zhao, L Liu, Y Chen, D Ai, Y Yao, et al. The Current Situation of Internet Addiction and Its Impact on Sleep Quality and Self-Injury Behavior in Chinese Medical Students. Psychiatry investigation. 2020;17(4):385.

496. Y Yang, Q Miao, X Zhu, L Qin, W Gong, S Zhang, et al. Sleeping Time, BMI, and Body Fat in Chinese Freshmen and Their Interrelation. Obesity facts. 2020;13(2):179-90.

497. Y Zhang, Y Xiong, J Dong, T Guo, X Tang, Y Zhao. Caffeinated Drinks Intake, Late Chronotype, and Increased Body Mass Index among Medical Students in Chongqing, China: A Multiple Mediation Model. International journal of environmental research and public health. 2018;15(8).

498. Yavuz F, Kabaagil B, Ismailogullari S, Zararsiz G, Per H, Yavuz Fatih, et al. Investigation of the Prevalence of Sleep Disorders in Medical Students and Examination of Its Change by Classes, Gender and Body Mass Index. JOURNAL OF TURKISH SLEEP MEDICINE-TURK UYKU TIBBI DERGISI. 2019;6(3):88-92.

499. Yeung WF, Chung KF, Chan TC, Yeung Wing-Fai, Chung Ka-Fai, Chan Tommy Cy. Sleep-wake habits, excessive daytime sleepiness and academic performance among medical students in Hong Kong. BIOLOGICAL RHYTHM RESEARCH. 2008;39(4):369-77.

500. YH Hsieh, CY Hsu, CY Liu, TL Huang. The levels of stress and depression among interns and clerks in three medical centers in Taiwan--a cross-sectional study. Chang Gung medical journal. 2011;34(3):278-85.

501. YH Lin, YC Ho, SH Lin, YH Yeh, CY Liu, TB Kuo, et al. On-call duty effects on sleep-state physiological stability in male medical interns. PloS one. 2013;8(6):e65072.

502. YH Wan, R Gao, XY Tao, FB Tao, CL Hu. [Relationship between deliberate self-harm and suicidal behaviors in college students]. China2012 2012-5. 474-7 p.

503. YL Venevtseva, AK Melnikov, LV Putilin. [Psychosocial factors influencing the dynamics and sleep quality in medical students]. Zhurnal nevrologii i psikhiatrii imeni SS Korsakova. 2021;121(4):70-6.

504. YM Alrebdi, AK Awadh, MS Alfehaid, AA Alsindi, A Alaraj. Knowledge and Attitude Regarding Sleep Medicine among Medical Students at Qassim University, Saudi Arabia. Open access Macedonian journal of medical sciences. 2019;7(17):2895-901.

505. YM Al-Jehani, AM Althwanay, HM Buainain, AK Abuhaimed, AM Almulhim, FA Abusrir, et al. Burnout Prevalence and Associated Stressors in Medical Students of Traditional and Problem-Based Learning Curricula in a Saudi University. Saudi journal of medicine & medical sciences. 2020;8(2):125-32.

506. YM Tan, KL Goh, R Muhidayah, CL Ooi, O Salem. Prevalence of irritable bowel syndrome in young adult Malaysians: a survey among medical students. Journal of gastroenterology and hepatology. 2003;18(12):1412-6.

507. Yokusoglu C, Atasoy M, Tekeli N, Ural A, Ulus C, Taylan Y, et al. A Survey Focusing on Lucid Dreaming, Metacognition, and Dream Anxiety in Medical Students. NOROPSIKIYATRI ARSIVI-ARCHIVES OF NEUROPSYCHIATRY. 2017;54(3):255-9.

508. Z Bunjo, LJ Bunjo, S Bacchi, F Donnelly, JN Hudson, I Symonds. Sleep Patterns and Risky Driving Behaviors in Clinical Medical and Nursing Students. United States2019 2019-10. 555-6 p.

509. Z Győrffy, I Csala, I Sándor. [Medical students of Hungary. A changing profession or feminisation?]. Hungary2013 2013-12-8. 1950-8 p.

510. Z Liu, R Liu, Y Zhang, R Zhang, L Liang, Y Wang, et al. Association between perceived stress and depression among medical students during the outbreak of COVID-19: The mediating role of insomnia. Journal of affective disorders. 2021;292:89-94.

511. Z Pündük, H Gür, I Ercan. [A reliability study of the Turkish version of the mornings-evenings questionnaire]. Turkey2005 2005. 40-5 p.

512. Zailinawati AH, Teng CL, Chung YC, Teow TL, Lee PN, Jagmohni KS. Daytime sleepiness and sleep quality among Malaysian medical students. The Medical journal of Malaysia. 2009;64(2):108-10.

513. Zdun-Ryżewska A, Basiński K, Michalik A. A confirmatory factor analysis for an adapted and validated Polish version of the <i>Chalder Fatigue Questionnair</i>e. International journal of occupational medicine and environmental health. 2020;33(1):67-76.

514. Zeru AB, Gebeyaw ED, Ayele ET. Magnitude and associated factors of menstrual irregularity among undergraduate students of Debre Berhan University, Ethiopia. Reproductive health. 2021;18(1):101.

515. Zureick AH, Burk-Rafel J, Purkiss JA, Hortsch M. The interrupted learner: How distractions during live and video lectures influence learning outcomes. Anatomical sciences education. 2018;11(4):366-76.

516. Alsulami A, Bakhsh D, Baik M, Merdad M, Aboalfaraj N. Assessment of Sleep Quality and its Relationship to Social Media Use Among Medical Students. Medical science educator. 2019;29(1):157-61.

517. Anderson J, Beckitt D, Geal MA, Cocker P. How clinical students spend their time. British journal of medical education. 1968;2(1):4-10.

518. Assefa ZM, Haile TG, Wazema DH, Tafese WT, Berrie FW, Beketie ED, et al. Mental Health Disorders During COVID-19 Pandemic Among Southwest Ethiopia University Students: An Institutional-Based Cross-Sectional Study. SAGE OPEN NURSING. 2021;7.

519. Attal BA, Bezdan M, Abdulqader A. Quality of Sleep and Its Correlates among Yemeni Medical Students: A Cross-Sectional Study. Sleep disorders. 2021;2021:8887870.

520. Aung MN, Somboonwong J, Jaroonvanichkul V, Wannakrairot P, Aung Myo Nyein, Somboonwong Juraiporn, et al. Possible Link Between Medical Students' Motivation for Academic Work and Time Engaged in Physical Exercise. MIND BRAIN AND EDUCATION. 2016;10(4):264-71.

521. Bahammam AS, Al-Khairy OK, Al-Taweel AA. Sleep habits and patterns among medical students. Neurosciences (Riyadh, Saudi Arabia). 2005;10(2):159-62.

522. Bahammam AS, Alaseem AM, Alzakri AA, Almeneessier AS, Sharif MM. The relationship between sleep and wake habits and academic performance in medical students: a cross-sectional study. BMC medical education. 2012;12:61.

523. Bajwa MS, Bashir MM, Jawairia NE, Bajwa Momina Shahzad, Bashir Muhammad Mubeen, Jawairia Noor E. ATTRIBUTES OF SUCCESS AMONG MEDICAL STUDENTS TO PASS THE PROFESSIONAL. INDO AMERICAN JOURNAL OF PHARMACEUTICAL SCIENCES. 2018;5(8):7191-6.

524. Bandi PS, Raju TSN, Rani SR, Bandi Pushyami Satya, Raju T. S. N, Rani S. Radha. Smart Phone Addiction And Associated Sleep Disturbances In Medical Students. INDIAN JOURNAL OF PSYCHIATRY. 2017;59(6):S215-S.

525. Barahona-Correa JE, Aristizabal-Mayor JD, Lasalvia P, Ruiz ÁJ, Hidalgo-Martínez P. Sleep disturbances, academic performance, depressive symptoms and substance use among medical students in Bogota, Colombia. Sleep science (Sao Paulo, Brazil). 2018;11(4):260-8.

526. Baran S, Teul-Swiniarska I, Dzieciolowska-Baran E, Lorkowski J, Gawlikowska-Sroka A, Baran Sylwia, et al., editors. Mental Health of Polish Students and the Occurrence of Respiratory Tract Infections2013 2013; 233 SPRING STREET, NEW YORK, NY 10013, UNITED STATES: SPRINGER.

527. Barbosa-Medeiros MR, Figueiredo JFLM, Melo LD, Rossi-Barbosa LAR, Caldeira AP, Barbosa-Medeiros Mirna Rossi, et al. FACTORS ASSOCIATED WITH DAYTIME SLEEPINESS IN MEDICAL STUDENTS. REVISTA DE PESQUISA-CUIDADO E FUNDAMENTAL ONLINE. 2021;13:774-9.

528. Barbosa-Medeiros MR, Silva MO, de Assis KBO, Rossi-Barbosa LAR, Caldeira AP, Barbosa-Medeiros Mirna Rossi, et al. Risk Behaviors for Chronic Noncommunicable Diseases in Medical Students. MUNDO DA SAUDE. 2021;45(1):299-307.

529. Basdav J, Haffejee F, Puckree T, Basdav Jyotika, Haffejee Firoza, Puckree T. Impact of headaches on university students in Durban, South Africa. SPRINGERPLUS. 2016;5.

530. Bin Saif GA, Alotaibi HM, Alzolibani AA, Almodihesh NA, Albraidi HF, Alotaibi NM, et al. Association of psychological stress with skin symptoms among medical students. SAUDI MEDICAL JOURNAL. 2018;39(1):59-66.

531. Biswas B, Haldar A, Dasgupta A, Sembiah S, Karmakar A, Mallick N, et al. An Epidemiological Study on Burden of Psychological Morbidities and Their Determinants among Undergraduate Medical Students of a Government Medical College of Eastern India. INDIAN JOURNAL OF COMMUNITY HEALTH. 2018;30(3):280-6.

532. Blau JN. Common headaches: type, duration, frequency and implications. Headache. 1990;30(11):701-4.

533. Boozari B, Saneei P, Safavi SM. Association between sleep duration and sleep quality with sugar and sugar-sweetened beverages intake among university students. Sleep & breathing = Schlaf & Atmung. 2021;25(2):649-56.

534. Browne BJ, Van Susteren T, Onsager DR, Simpson D, Salaymeh B, Condon RE. Influence of sleep deprivation on learning among surgical house staff and medical students. Surgery. 1994;115(5):604-10.

535. Brubaker JR, Beverly EA. Burnout, Perceived Stress, Sleep Quality, and Smartphone Use: A Survey of Osteopathic Medical Students. The Journal of the American Osteopathic Association. 2020;120(1):6-17.

536. Brubaker JR, Swan A, Beverly EA. A brief intervention to reduce burnout and improve sleep quality in medical students. BMC medical education. 2020;20(1):345.

537. Chen B, Liu F, Ding S, Ying X, Wang L, Wen Y. Gender differences in factors associated with smartphone addiction: a cross-sectional study among medical college students. BMC psychiatry. 2017;17(1):341.

538. Chinawa JM, Chukwu BF, Obu HA. Sleep practices among medical students in Pediatrics Department of University of Nigeria Teaching Hospital, Ituku/Ozalla, Enugu, Nigeria. India2014 2014-3. 232-6 p.

539. Cleland J, Gates LJ, Waiter GD, Ho VB, Schuwirth L, Durning S. Even a little sleepiness influences neural activation and clinical reasoning in novices. Health science reports. 2021;4(4):e406.

540. Dagnew B, Andualem Z, Dagne H. Excessive daytime sleepiness and its predictors among medical and health science students of University of Gondar, Northwest Ethiopia: institution-based cross-sectional study. Health and quality of life outcomes. 2020;18(1):299.

541. Dagnew B, Dagne H, Andualem Z. Depression and Its Determinant Factors Among University of Gondar Medical and Health Science Students, Northwest Ethiopia: Institution-Based Cross-Sectional Study. Neuropsychiatric disease and treatment. 2020;16:839-45.

542. Fernandez-Mendoza J, Rodriguez-Muñoz A, Vela-Bueno A, Olavarrieta-Bernardino S, Calhoun SL, Bixler EO, et al. The Spanish version of the Insomnia Severity Index: a confirmatory factor analysis. Sleep medicine. 2012;13(2):207-10.

543. Ficker JH, Wiest GH, Lehnert G, Meyer M, Hahn EG. Are snoring medical students at risk of failing their exams? United States1999 1999-3-15. 205-9 p.

544. Greeson JM, Toohey MJ, Pearce MJ. An adapted, four-week mind-body skills group for medical students: reducing stress, increasing mindfulness, and enhancing self-care. United States2015 2015-5. 186-92 p.

545. Guo J, Meng D, Ma X, Zhu L, Yang L, Mu L. The impact of bedtime procrastination on depression symptoms in Chinese medical students. Sleep & breathing = Schlaf & Atmung. 2020;24(3):1247-55.

546. Harolds JA. Quality and Safety in Healthcare, Part LXVII: Counseling, Mental Health Services, and Wellness Curricula for Medical Students. Clinical nuclear medicine. 2021;46(3):220-2.

547. Islam MS, Akter R, Sikder MT, Griffiths MD, Islam Md Saiful, Akter Rejina, et al. Weight-Related Status and Associated Predictors with Psychological Well-being among First-Year University Students in Bangladesh: A Pilot Study. INTERNATIONAL JOURNAL OF MENTAL HEALTH AND ADDICTION.

548. Islas-Marroquín J, Delgado-Brambila HA. Studies on nap sleep in young students. Relationships between polygraphic data and the occurrence of dreams in replacing naps. United States1998 1998. 149-53 p.

549. Isman C, Gulen S, Gundogan N, Isman Cagla, Gulen Sebnem, Gundogan Nimet. The Relationship Between Digit Ratio and Circadian Typology Among Medical Students. TRAKYA UNIVERSITESI TIP FAKULTESI DERGISI. 2010;27(1):68-73.

550. Jandaghi G, Khalajinia Z, Jandaghi Gholamreza, Khalajinia Zohreh. Comparing lifestyles, social support, body mass index and history of menses between Mashhad University of Medical Sciences students with and without primary dysmenorrheal. SCIENTIFIC RESEARCH AND ESSAYS. 2010;5(18):2752-5.

551. Jarrett NL, Yamane DE, Gildner DJ, Pickett SM, Jarrett Nicole L, Yamane Daniel E, et al. THE INDIRECT EFFECT OF SLEEP QUALITY ON EMOTIONAL EXHAUSTION THROUGH EMOTION REGULATION DIFFICULTIES AND PERCEIVED STRESS IN A SAMPLE OF US MEDICAL STUDENTS. SLEEP. 2019;42.

552. Javadi SAHS, Javadi S. A. Haji Seyed. Evaluation of depression and anxiety, and their relationship with insomnia, nightmare and demographic variables in medical students. EUROPEAN PSYCHIATRY. 2017;41:S853-S.

553. Javed S, Safdar N, Ali AA, Azeem M, Javed Shoaib, Safdar Nouman, et al. To Study the Quality and Patterns of Sleep in Relation to Consumption of Energy Drinks among medical students of AllamaIqbal Medical College, Lahore. PAKISTAN JOURNAL OF MEDICAL & HEALTH SCIENCES. 2017;11(2):683-5.

554. Jin YL, Ding ZY, Fei Y, Jin W, Liu H, Chen ZX, et al. Social relationships play a role in sleep status in Chinese undergraduate students. PSYCHIATRY RESEARCH. 2014;220(1):631-8.

555. Johnson K, Simon N, Wicks M, Schaad D, Barr KP, O'Connor K, et al. SLEEP HABITS, QUALITY OF LIFE AND SATISFACTION WITH ACADEMIC SUCCESS IN SECOND YEAR MEDICAL STUDENTS. JOURNAL OF GENERAL INTERNAL MEDICINE. 2014;29:S209-S.

556. Joshi AR, Nagpal M, Joshi Anuradha Rajiv, Nagpal Mitsha. Assessment of Perceived Stress in Postgraduate Medical Students during Training Programme. JOURNAL OF CLINICAL AND DIAGNOSTIC RESEARCH. 2018;12(6):CC01-CC4.

557. Jr Baldwin DC, SR Daugherty, EJ Eckenfels, L Leksas. The experience of mistreatment and abuse among medical students. Research in medical education : proceedings of the annual Conference Conference on Research in Medical Education. 1988;27:80-4.

558. Jr Parkerson GR, WE Broadhead, CK Tse. The health status and life satisfaction of first-year medical students. Academic medicine : journal of the Association of American Medical Colleges. 1990;65(9):586-8.

559. JR Webb, JW Thomas, MA Valasek. Contemplating cognitive enhancement in medical students and residents. Perspectives in biology and medicine. 2010;53(2):200-14.

560. JU Ohaeri, AO Odejide, BA Ikuesan, JD Adeyemi. The pattern of isolated sleep paralysis among Nigerian medical students. Journal of the National Medical Association. 1989;81(7):805-8.

561. JY Al-Hashel, SF Ahmed, R Alroughani, PJ Goadsby. Migraine among medical students in Kuwait University. The journal of headache and pain. 2014;15(1):26.

562. K Ahrberg, M Dresler, S Niedermaier, A Steiger, L Genzel. The interaction between sleep quality and academic performance. Journal of psychiatric research. 2012;46(12):1618-22.

563. K Kongsomboon. Psychological problems and overweight in medical students compared to students from Faculty of Humanities, Srinakharinwirot University, Thailand. Journal of the Medical Association of Thailand = Chotmaihet thangphaet. 2010;93:S106-13.

564. K Kheirallah, S Bloukh, W Khasawneh, J Alsulaiman, A Khassawneh, AH Al-Mistarehi, et al. Medical students' relative immunity, or lack thereof, against COVID-19 emotional distress and psychological challenges; a descriptive study from Jordan. F1000Research. 2021;10:297.

565. K Nugent, R Raj, R Nugent. Sleep Patterns and Health Behaviors in Healthcare Students. Southern medical journal. 2020;113(3):104-10.

566. K Puvanendran, J Venkatramani, A Jain, M Farid. Sleep deprivation in junior doctors--house officers in Singapore. Industrial health. 2005;43(1):129-32.

567. K Parmar, R Tandon, N Kumar, RK Garg. Variations in electroencephalography with mobile phone usage in medical students. Neurology India. 2019;67(1):235-41.

568. K Roka, S Khadka, S Dahal, M Yadav, P Thapa, R Kc. Excessive Daytime Sleepiness among First to Fourth Year Undergraduate Students of a Medical College in Nepal: A Descriptive Cross-sectional Study. JNMA; journal of the Nepal Medical Association. 2020;58(229):640-4.

569. K Suwan, P Hatthachote, S Panichkul, V Phromphetcharat. Comparision of overweight and obesity in medical cadets before and after 6 months studying at Phramongkutklao College. Thailand2012 2012-5. S142-8 p.

570. K Sandefur, T Kondrashova. Increasing Self-Awareness of Medical Students Through the Use of Ultrasonography. The Journal of the American Osteopathic Association. 2018;118(3):190-8.

571. K Yeluri, K Hs, BG H, SC Bj. Electronic Gadget Screen-time, Perceived Sleep Quality & Quantity and Academic Performance in Medical Students. The Journal of the Association of Physicians of India. 2021;69(11):11-2.

572. K Zikmundová, H Zavázalová, S Vozehová, F Lavicka. [Characteristics of the quality of life of medical students]. Czech Republic2002 2002. 553-7 p.

573. KA Bin Abdulrahman, AM Khalaf, FB Bin Abbas, OT Alanezi. The Lifestyle of Saudi Medical Students. International journal of environmental research and public health. 2021;18(15).

574. Kanjo M, Alsaati RH, Jassomah OM, Alhindi SH, Jamjoom LF, Albogami MA, et al. The prevalence of migraine headache among students of Fakeeh College in Jeddah, Saudi Arabia. MEDICAL SCIENCE. 2021;25(108):320-7.

575. Kao PC, Kao Po-Chi. Medical students' attention in EFL class: roles of academic expectation stress and quality of sleep. APPLIED LINGUISTICS REVIEW.

576. Kashyap A, Rathi MM, Umashankar M, Kashyap Aditya, Rathi Madhur M., Umashankar M. A STUDY OF PREVALENCE OF SMARTPHONE DEPENDENCE AND ITS IMPACT ON SLEEP PATTERN AMONG MEDICAL STUDENTS. INDIAN JOURNAL OF PSYCHIATRY. 2019;61(9):S505-S.

577. Kay J, Howard T, Welch G. Health habits of medical students: some perils of the profession. Journal of the American College Health Association. 1980;28(4):238-9.

578. KC de Souza, TB Mendes, THS Gomes, AA da Silva, LHDS Nali, ALL Bachi, et al. Medical Students Show Lower Physical Activity Levels and Higher Anxiety Than Physical Education Students: A Cross-Sectional Study During the COVID-19 Pandemic. Frontiers in psychiatry. 2021;12:804967.

579. KD Sandoval, PV Morote-Jayacc, M Moreno-Molina, A Taype-Rondan. [Depression, stress and anxiety in students of human medicine in Ayacucho (Peru) in the context of the COVID-19 pandemic]2021 2021-11-9.

580. Kelly F, Federle VHDS, Oliveira M, Passos FG, Hettwer M, Kelly Francinny, et al. Influence of COVID-19 in medical students' sleeping circle. JOURNAL OF THE NEUROLOGICAL SCIENCES. 2021;429.

581. Kelly MR, Haynes P, Kelly M. R., Haynes P. Emotion regulation and sleep in medical students: A preliminary examination. SLEEP. 2008;31:A350-A.

582. KG Gomathi, S Ahmed, J Sreedharan. Psychological health of first-year health professional students in a medical university in the United arab emirates. Sultan Qaboos University medical journal. 2012;12(2):206-13.

583. Khullar S, Singh M, Das S, Kamya, Khullar Shilpa, Singh Mitasha, et al. To study the association between daytime sleepiness and cognition as tested by stroop test in Indian medical students. JOURNAL OF THE SCIENTIFIC SOCIETY. 2021;48(1):33-7.

584. Kim B, Roh H. Depressive symptoms in medical students: prevalence and related factors. Korean journal of medical education. 2014;26(1):53-8.

585. Kim J. Analysis of health consumers' behavior using self-tracker for activity, sleep, and diet2014 2014-6. 552-8 p.

586. KM Johnson, N Simon, M Wicks, K Barr, K O'Connor, D Schaad. Amount of Sleep, Daytime Sleepiness, Hazardous Driving, and Quality of Life of Second Year Medical Students. Academic psychiatry : the journal of the American Association of Directors of Psychiatric Residency Training and the Association for Academic Psychiatry. 2017;41(5):669-73.

587. Kongsomboon K, Neruntarat C, Kongsomboon Kittipong, Neruntarat Chairat. Sleep-disordered breathing and risk factors in Thailand. ASIAN BIOMEDICINE. 2011;5(4):519-23.

588. KP Lee, N Yeung, C Wong, B Yip, LHF Luk, S Wong. Prevalence of medical students' burnout and its associated demographics and lifestyle factors in Hong Kong. PloS one. 2020;15(7):e0235154.

589. KRASNOPEROV OV, PANCHENKO AL, KRASNOPEROV OV, PANCHENKO AL. INTERRELATIONS OF SUBJECTIVE CHARACTERISTICS OF SLEEP AND PERSONALITY-TRAITS. VOPROSY PSIKHOLOGII. 1991(6):139-42.

590. Krieger J, Turlot JC, Mangin P, Kurtz D. Breathing during sleep in normal young and elderly subjects: hypopneas, apneas, and correlated factors. Sleep. 1983;6(2):108-20.

591. Krishnan B, Sanjeev RK, Latti RG. Quality of Sleep Among Bedtime Smartphone Users. International journal of preventive medicine. 2020;11:114.

592. KS Purim, AT Guimarães, AC Titski, N Leite. Sleep deprivation and drowsiness of medical residents and medical students. Revista do Colegio Brasileiro de Cirurgioes. 2016;43(6):438-44.

593. Kumar S, Mahour J, Arjariya R, Singh J, Kumar Shailesh, Mahour Jitendra, et al. Changes in Sleep Across Different Phases of Life of Medical Students and Impact of Home Confinement on Sleep during COVID-19 Lockdown. JOURNAL OF CLINICAL AND DIAGNOSTIC RESEARCH. 2020;14(11):CC4-CC7.

594. Kumar TD, Anupama P, Kumar T. Dinesh, Anupama P. STUDY OF USAGE PATTERNS AND EFFECTS OF SMART PHONE AMONG THE MEDICAL STUDENTS OF NIZAMABAD, TELANGANA. JOURNAL OF EVOLUTION OF MEDICAL AND DENTAL SCIENCES-JEMDS. 2019;8(26):2081-5.

595. L AlFakhri, J Sarraj, S Kherallah, K Kuhail, A Obeidat, A Abu-Zaid. Perceptions of pre-clerkship medical students and academic advisors about sleep deprivation and its relationship to academic performance: a cross-sectional perspective from Saudi Arabia. BMC research notes. 2015;8:740.

596. L Fang, X Xu, X Lin, Y Chen, F Zheng, Y Bei, et al. [Association of mobile phone overuse with sleep disorder and unhealthy eating behaviors in college students of a medical university in Guangzhou]. Nan fang yi ke da xue xue bao = Journal of Southern Medical University. 2019;39(12):1500-5.

597. L Genzel, K Ahrberg, C Roselli, S Niedermaier, A Steiger, M Dresler, et al. Sleep timing is more important than sleep length or quality for medical school performance. Chronobiology international. 2013;30(6):766-71.

598. Lapinski J, Yost M, Sexton P, LaBaere RJ 2nd. Factors Modifying Burnout in Osteopathic Medical Students. Academic psychiatry : the journal of the American Association of Directors of Psychiatric Residency Training and the Association for Academic Psychiatry. 2016;40(1):55-62.

599. Liu BH, Huang YQ, Niu WY, Lv ZZ, Yue C, Wang PY. [Study on the factors influencing suicidal ideation among medical students in Beijing]. China2008 2008-2. 128-31 p.

600. Lu J, Fang GE, Shen SJ, Wang Y, Sun Q. A Questionnaire survey on sleeping in class phenomenon among Chinese medical undergraduates. England2011 2011. 508 p.

601. Luo JM, Liu EZ, Yang HD, Du CZ, Xia LJ, Zhang ZC, et al. Prevalence and Factors Associated With Suicidal Ideation in Medical Students With Migraine. Frontiers in psychiatry. 2021;12:683342.

602. M Amiri, B Dowran. Smartphone Overuse from Iranian University Students' Perspective: A Qualitative Study. Addiction & health. 2020;12(3):205-15.

603. M Alshahrani, Y Al Turki. Sleep hygiene awareness: Its relation to sleep quality among medical students in King Saud University, Riyadh, Saudi Arabia. Journal of family medicine and primary care. 2019;8(8):2628-32.

604. M Al Kazhali, M Shahwan, N Hassan, AA Jairoun. Social media use is linked to poor sleep quality: The opportunities and challenges to support evidence-informed policymaking in the UAE. Journal of public health (Oxford, England). 2021.

605. M Belingheri, A Pellegrini, R Facchetti, G De Vito, G Cesana, MA Riva. Self-reported prevalence of sleep disorders among medical and nursing students. Occupational medicine (Oxford, England). 2020;70(2):127-30.

606. M Dąbrowska-Galas, K Ptaszkowski, J Dąbrowska. Physical Activity Level, Insomnia and Related Impact in Medical Students in Poland. International journal of environmental research and public health. 2021;18(6).

607. M Fawzy, SA Hamed. Prevalence of psychological stress, depression and anxiety among medical students in Egypt. Psychiatry research. 2017;255:186-94.

608. M Gromadecka-Sutkiewicz, J Chraplewska. [Rhythm of life of Poznan Medical University students' and hygienic norms]. Poland2005 2005. 41-4 p.

609. M Irfan, MR Sethi, AS Abdullah, U Saleem, D Khan. Psychological distress in students appearing for the medical school entrance examination in Peshawar. JPMA The Journal of the Pakistan Medical Association. 2018;68(11):1603-7.

610. M Israel, U Patil, S Shinde, VM Ruikar. Obesity in Medical Students and its Correlation with Sleep Patterns and Sleep Duration. Indian journal of physiology and pharmacology. 2016;60(1):38-44.

611. M Karimy, F Parvizi, MR Rouhani, MD Griffiths, B Armoon, L Fattah Moghaddam. The association between internet addiction, sleep quality, and health-related quality of life among Iranian medical students. Journal of addictive diseases. 2020;38(3):317-25.

612. M Khero, M Fatima, MAA Shah, A Tahir. Comparison of the Status of Sleep Quality in Basic and Clinical Medical Students. Cureus. 2019;11(3):e4326.

613. M Luo, Y Feng, T Li. Sleep medicine knowledge, attitudes, and practices among medical students in Guangzhou, China. Sleep & breathing = Schlaf & Atmung. 2013;17(2):687-93.

614. M Nacar, F Cetinkaya, Z Baykan, G Yilmazel, F Elmali. Hazardous Health Behaviour among Medical Students: a Study from Turkey. Asian Pacific journal of cancer prevention : APJCP. 2015;16(17):7675-81.

615. M Nojomi, MF Ghalhe Bandi, S Kaffashi. Sleep pattern in medical students and residents. Archives of Iranian medicine. 2009;12(6):542-9.

616. M Nisar, RM Mohammad, A Arshad, I Hashmi, SM Yousuf, S Baig. Influence of Dietary Intake on Sleeping Patterns of Medical Students. Cureus. 2019;11(2):e4106.

617. M Najafi Kalyani, N Jamshidi, J Salami, E Pourjam. Investigation of the Relationship between Psychological Variables and Sleep Quality in Students of Medical Sciences. Depression research and treatment. 2017;2017:7143547.

618. M Okutani, S Komori, H Iwasaki, Y Mochizuki, I Kohno, S Mochizuki, et al. What time is the "biologic zero hour" of circadian variability? United States1997 1997-7. 756-62 p.

619. M Olarte-Durand, JB Roque-Aycachi, R Rojas-Humpire, JF Canaza-Apaza, S Laureano, A Rojas-Humpire, et al. [Mood and sleep quality in Peruvian medical students during COVID-19 pandemic]2021 2021-12-9.

620. M Ranjbaran, B Soleimani, M Mohammadi, N Ghorbani, M Khodadost, K Mansori, et al. Association between General Health and Mobile Phone Dependency among Medical University Students: A Cross-sectional Study in Iran. International journal of preventive medicine. 2019;10:126.

621. M Rezaei, M Khormali, S Akbarpour, K Sadeghniiat-Hagighi, M Shamsipour. Sleep quality and its association with psychological distress and sleep hygiene: a cross-sectional study among pre-clinical medical students. Sleep science (Sao Paulo, Brazil). 2018;11(4):274-80.

622. Meng J, Wang F, Chen R, Hua H, Yang Q, Yang D, et al. Association between the pattern of mobile phone use and sleep quality in Northeast China college students. Sleep & breathing = Schlaf & Atmung. 2021;25(4):2259-67.

623. Menon B, Kinnera N. Prevalence and characteristics of migraine in medical students and its impact on their daily activities. Annals of Indian Academy of Neurology. 2013;16(2):221-5.

624. MG Kamath, J Prakash, S Dash, S Chowdhury, ZB Ahmed, MZ Yusof. "Is there an Association Between Self-Reported Sleep Duration, Body Mass Index and Waist-Hip Ratio in Young Adults? A Cross-Sectional Pilot Study". Journal of clinical and diagnostic research : JCDR. 2014;8(9):BC05-7.

625. MH Taha, K Shehzad, AS Alamro, M Wadi. Internet Use and Addiction Among Medical Students in Qassim University, Saudi Arabia. Sultan Qaboos University medical journal. 2019;19(2):e142-e7.

626. MH Zainaldeen, NE Hasan, FAH Ahmed Ali, HS Altahoo, F Rashid-Doubell, S Fredericks. The influence of ball-juggling on emotional states, blood pressure and sleep-quality among medical students during end-of-year exam preparation. Complementary therapies in clinical practice. 2018;30:64-7.

627. MI Al Asqah, AI Al Orainey, MA Shukr, HM Al Oraini, YA Al Turki. The prevalence of internet gaming disorder among medical students at King Saud University, Riyadh, Saudi Arabia. A cross-sectional study. Saudi medical journal. 2020;41(12):1359-63.

628. Michaeli D, Keough G, Strotzer Q, Michaeli T, Michaeli Daniel, Keough Gregory, et al. Digital medical education and students' mental health: effects of the COVID-19 pandemic in Germany. JOURNAL OF MENTAL HEALTH TRAINING EDUCATION AND PRACTICE.

629. Migacz E, Wichniak A, Kukwa W, Migacz E., Wichniak A., Kukwa W. Are questionnaires reliable in diagnosing sleep-disordered breathing in university students? JOURNAL OF LARYNGOLOGY AND OTOLOGY. 2017;131(11):965-71.

630. Mihaltan F, Oana D, Lorena C, Ruxandra U, Mihaltan F., Oana D., et al. Sleep Disorders Among Medical Students In Romania. AMERICAN JOURNAL OF RESPIRATORY AND CRITICAL CARE MEDICINE. 2017;195.

631. MJ Oura, AR Moreira, P Santos. Stress among Portuguese Medical Students: A National Cross-Sectional Study. Journal of environmental and public health. 2020;2020:6183757.

632. MJ Tahir, NI Malik, I Ullah, HR Khan, S Perveen, R Ramalho, et al. Internet addiction and sleep quality among medical students during the COVID-19 pandemic: A multinational cross-sectional survey. PloS one. 2021;16(11):e0259594.

633. ML Mellum, AH Vestergaard, J Grauslund, AS Vergmann. Virtual vitreoretinal surgery: effect of distracting factors on surgical performance in medical students. Acta ophthalmologica. 2020;98(4):378-83.

634. ML Matthews, P Gross, WN Herbert. Post-call cognitive function and satisfaction in medical students on different call schedules: a prospective observational pilot study. American journal of obstetrics and gynecology. 2006;195(5):1484-8.

635. MM Halbach, CO Spann, G Egan. Effect of sleep deprivation on medical resident and student cognitive function: A prospective study. American journal of obstetrics and gynecology. 2003;188(5):1198-201.

636. MM Khan. Adverse effects of excessive mobile phone use. International journal of occupational medicine and environmental health. 2008;21(4):289-93.

637. MN Tan, V Mevsim, M Pozlu Cifci, H Sayan, AE Ercan, OF Ergin, et al. Who is happier among preclinical medical students: the impact of chronotype preference. Chronobiology international. 2020;37(8):1163-72.

638. Modna Y, Scott B, Modna Yuliya, Scott Bernadette. THE ROLE OF CIRCADIAN RHYTHMS AMONG MEDICAL STUDENTS IN TIME MANAGEMENT ORGANIZATION AND ACADEMIC ACHIEVEMENT. POD VODARENSKOU VEZI 4, PRAGUE 8, 18200, CZECH REPUBLIC: CENTRAL BOHEMIA UNIV; 2017 2017. 983-7 p.

639. Moharana S, Lipika M, Moharana DN, Pattnaik SS, Moharana Sandhyarani, Lipika M., et al. Yoga as a Health Promotion Lifestyle Tool: A Study on Medical Students from a Tertiary Care Centre. INTERNATIONAL JOURNAL OF SCIENTIFIC STUDY. 2017;5(3):272-6.

640. Monsalves S, Valladres X, Veloz M, Ocampo-Garces A, Monsalves S, Valladres X, et al. Sleepiness in medical students. JOURNAL OF THE NEUROLOGICAL SCIENCES. 2003;214(1):107-8.

641. Moon HJ, Yoo S, Cho YW, Moon H. J., Yoo S., Cho Y. W. The effect of chronotype and social jetlag on sleep, mental health, quality of life, and academic performance of medical students. JOURNAL OF THE NEUROLOGICAL SCIENCES. 2017;381:296-.

642. Morgan C, Attarian H, Guo R, Viola-Saltzman M, Morgan C., Attarian H., et al. EXCESSIVE SLEEPINESS AND POOR SLEEP QUALITY IN MEDICAL STUDENTS. SLEEP. 2012;35:A444-A.

643. Morgenthaler J, Wiesner CD, Hinze K, Abels LC, Prehn-Kristensen A, Göder R. Selective REM-sleep deprivation does not diminish emotional memory consolidation in young healthy subjects. PloS one. 2014;9(2):e89849.

644. Mozafari A, Mohamadi H, Tabaraie M, Arsang S, Mozafari A., Mohamadi H., et al. Morningness-eveningness chronotypes, sleep quality and insomnia among medical student of Qom. SLEEP MEDICINE. 2015;16:S100-S.

645. MP Hidalgo, CM de Souza, CB Zanette, PV Nunes. Association of daytime sleepiness and the morningness/eveningness dimension in young adult subjects in Brazil. Psychological reports. 2003;93(2):427-34.

646. MP Hidalgo, W Caumo. Sleep disturbances associated with minor psychiatric disorders in medical students. Neurological sciences : official journal of the Italian Neurological Society and of the Italian Society of Clinical Neurophysiology. 2002;23(1):35-9.

647. MP Loayza H, TS Ponte, CG Carvalho, MR Pedrotti, PV Nunes, CM Souza, et al. Association between mental health screening by self-report questionnaire and insomnia in medical students. Arquivos de neuro-psiquiatria. 2001;59(2):180-5.

648. MQ Yu, FS Dong, RD Song, HJ Liu, GY Ren, BY Huang, et al. [Study on effect of snore guard to upper airway structure of normal occlusion people by magnetic resonance imaging]. Hua xi kou qiang yi xue za zhi = Huaxi kouqiang yixue zazhi = West China journal of stomatology. 2007;25(4):349-53.

649. MR Shadzi, A Salehi, HM Vardanjani. Problematic Internet Use, Mental Health, and Sleep Quality among Medical Students: A Path-Analytic Model. Indian journal of psychological medicine. 2020;42(2):128-35.

650. MR Wolf, JB Rosenstock. Inadequate Sleep and Exercise Associated with Burnout and Depression Among Medical Students. Academic psychiatry : the journal of the American Association of Directors of Psychiatric Residency Training and the Association for Academic Psychiatry. 2017;41(2):174-9.

651. MT Brannick, GT Horn, MJ Schnaus, MM Wahi, SB Goldin. Medical student quality-of-life in the clerkships: a scale validation study. The American surgeon. 2015;81(4):370-6.

652. Muanprasong S, Taneepanichskul N, Muanprasong Sirilak, Taneepanichskul Nutta. SLEEP QUALITY AND MIGRAINE STATUS AMONG UNDERGRADUATE STUDENTS IN A LARGE URBAN UNIVERSITY THAILAND. JOURNAL OF HEALTH RESEARCH. 2017;31:S25-S31.

653. Muraru ID, Munteanu C, Iorga M, Petrariu FD, Muraru Iulia-Diana, Munteanu Catalina, et al. INVESTIGATING MEDICAL STUDENTS' HEALTH CONCERNS DURING THEIR ACADEMIC STUDIES. MEDICAL-SURGICAL JOURNAL-REVISTA MEDICO-CHIRURGICALA. 2019;123(4):728-34.

654. MW Johns. Factor analysis of subjectively reported sleep habits, and the nature of insomnia. Psychological medicine. 1975;5(1):83-8.

655. MW Johns. Reliability and factor analysis of the Epworth Sleepiness Scale. Sleep. 1992;15(4):376-81.

656. MW Johns. Sleepiness in different situations measured by the Epworth Sleepiness Scale. United States1994 1994-12. 703-10 p.

657. MW Johns, DW Bruce, JP Masterton. Psychological correlates of sleep habits reported by healthy young adults. The British journal of medical psychology. 1974;47(2):181-7.

658. MW Johns, HA Dudley, JP Masterton. The sleep habits, personality and academic performance of medical students. Medical education. 1976;10(3):158-62.

659. MW Johns, TJ Gay, MD Goodyear, JP Masterton. Sleep habits of healthy young adults: use of a sleep questionnaire. British journal of preventive & social medicine. 1971;25(4):236-41.

660. MW Kortz, BM Kongs, DR Bisesi, M Roffler, RM Sheehy. A retrospective and correlative analysis of academic and nonacademic predictors of COMLEX level 1 performance. Journal of osteopathic medicine. 2022.

661. MZ Satti, TM Khan, QU Qurat-Ul-Ain, MJ Azhar, H Javed, M Yaseen, et al. Association of Physical Activity and Sleep Quality with Academic Performance Among Fourth-year MBBS Students of Rawalpindi Medical University. Cureus. 2019;11(7):e5086.

662. N Abdali, M Nobahar, R Ghorbani. Evaluation of emotional intelligence, sleep quality, and fatigue among Iranian medical, nursing, and paramedical students: A cross-sectional study. Qatar medical journal. 2019;2019(3):15.

663. N Ahmed, M Sadat, D Cukor. Sleep Knowledge and Behaviors in Medical Students: Results of a Single Center Survey. Academic psychiatry : the journal of the American Association of Directors of Psychiatric Residency Training and the Association for Academic Psychiatry. 2017;41(5):674-8.

664. N Akram, N Khan, M Ameen, S Mahmood, K Shamim, M Amin, et al. Morningness-eveningness preferences, learning approach and academic achievement of undergraduate medical students. Chronobiology international. 2018;35(9):1262-8.

665. N Christodoulou, J Maruani, MP d'Ortho, M Lejoyeux, PA Geoffroy. Sleep quality of medical students and relationships with academic performances. L'Encephale. 2021.

666. N Ergin, BB Kılıç, A Ergin, S Varlı. Sleep quality and related factors including restless leg syndrome in medical students and residents in a Turkish university. Sleep & breathing = Schlaf & Atmung. 2021.

667. N Erbil, H Yücesoy. Relationship between premenstrual syndrome and sleep quality among nursing and medical students. Perspectives in psychiatric care. 2020.

668. N Jaber, M Oudah, A Kowatli, J Jibril, I Baig, E Mathew, et al. Dietary and Lifestyle Factors Associated with Dyspepsia among Pre-clinical Medical Students in Ajman, United Arab Emirates. Central Asian journal of global health. 2016;5(1):192.

669. N Saeed, N Javed. Lessons from the COVID-19 pandemic: Perspectives of medical students. Pakistan journal of medical sciences. 2021;37(5):1402-7.

670. N Sundas, S Ghimire, S Bhusal, R Pandey, K Rana, H Dixit. Sleep Quality among Medical Students of a Tertiary Care Hospital: A Descriptive Cross-sectional Study. JNMA; journal of the Nepal Medical Association. 2020;58(222):76-9.

671. N Upadhayay, S Guragain. Internet use and its addiction level in medical students. Advances in medical education and practice. 2017;8:641-7.

672. N Wege, T Muth, J Li, P Angerer. Mental health among currently enrolled medical students in Germany. Public health. 2016;132:92-100.

673. NA Walsh, LM Repa, SN Garland. Mindful larks and lonely owls: The relationship between chronotype, mental health, sleep quality, and social support in young adults. Journal of sleep research. 2022;31(1):e13442.

674. Naeem A, Khan U, Ali A, Naeem Azka, Khan Urva, Ali Asad. EFFECT OF EXCESSIVE MOBILE PHONE USAGE ( HOURS) ON SLEEP PATTERNS AMONG THE MEDICAL STUDENTS OF KING EDWARD MEDICAL UNIVERSITY LAHORE. INDO AMERICAN JOURNAL OF PHARMACEUTICAL SCIENCES. 2018;5(7):6400-5.

675. Nagose V, Soni D, Rathod S, Yelne Y, Nagose Vaishali, Soni Dharmishta, et al. COVID-19 impact on mental health, sleep quality and various aspects of life of medical students and interns, and nonmedical students: A comparative study in Indian scenario. ANNALS OF INDIAN PSYCHIATRY. 2021;5(2):158-63.

676. Nasir U, Butt AF, Choudry S, Nasir Usama, Butt Ayesha Farooq, Choudry Sarah. A Study to Evaluate the Lifestyle of Medical Students in Lahore, Pakistan. CUREUS. 2019;11(3).

677. Nayak AK, Rath N, Mohapatra S, Pattnaik JI, Nayak Anil Kumar, Rath Neelmadhav, et al. Assesment Of Sleep Quality In Medical Students In A Tertiary Care Hospital. INDIAN JOURNAL OF PSYCHIATRY. 2017;59(6):S175-S.

678. Nayak A, Saranya K, Fredrick J, Madumathy R, Subramanian SK, Nayak Amrita, et al. Assessment of burden of internet addiction and its association with quality of sleep and cardiovascular autonomic function in undergraduate medical students. CLINICAL EPIDEMIOLOGY AND GLOBAL HEALTH. 2021;11.

679. NB Pokhrel, R Khadayat, P Tulachan. Depression, anxiety, and burnout among medical students and residents of a medical school in Nepal: a cross-sectional study. BMC psychiatry. 2020;20(1):298.

680. NC Aghukwa. Case histories of brainfag syndrome in Nigerian University undergraduates. Nigerian journal of medicine : journal of the National Association of Resident Doctors of Nigeria. 2014;23(4):315-20.

681. NE Penn, DF Kripke, J Scharff. Sleep paralysis among medical students. The Journal of psychology. 1981;107:247-52.

682. Nieradko B, Borzecki A. Exercise behavior, sleep habits and time management among students of the Medical University of Lublin. Annales Universitatis Mariae Curie-Sklodowska Sectio D: Medicina. 2003;58(1):358-61.

683. Niño García JA, Barragán Vergel MF, Ortiz Labrador JA, Ochoa Vera ME, González Olaya HL. Factors Associated with Excessive Daytime Sleepiness in Medical Students of a Higher Education Institution of Bucaramanga. Revista Colombiana de psiquiatria (English ed). 2019;48(4):222-31.

684. NK Ibrahim, AA Alghamdi, MM Almehmadi, AA Alzahrani, AK Turkistani, K Alghamdi. Allergy and related clinical symptoms among medical students and interns. Pakistan journal of medical sciences. 2019;35(4):1060-5.

685. NK Ibrahim, AK Alotaibi, AM Alhazmi, RZ Alshehri, RN Saimaldaher, MA Murad. Prevalence, predictors and triggers of migraine headache among medical students and interns in King Abdulaziz University, Jeddah, Saudi Arabia. Pakistan journal of medical sciences. 2017;33(2):270-5.

686. NK Ibrahim, BS Baharoon, WF Banjar, AA Jar, RM Ashor, AA Aman, et al. Mobile Phone Addiction and Its Relationship to Sleep Quality and Academic Achievement of Medical Students at King Abdulaziz University, Jeddah, Saudi Arabia. Journal of research in health sciences. 2018;18(3):e00420.

687. Noor S, Kayani AS, Shahid N, Ihsan A, Rasheed S, Tabassum MN, et al. Fast Food Intake Affecting Physical and Mental Well-Being of Medical Students in Lahore. PAKISTAN JOURNAL OF MEDICAL & HEALTH SCIENCES. 2021;15(8):1942-4.

688. Noordeen F, Jayathilaka T, Pitchai FNN, Yapa HRN, Jayarathne IMNMK, Nawarathne TND, et al. Stress coping mechanisms practiced by medical undergraduates of a State Medical School in Sri Lanka. SRI LANKA JOURNAL OF SOCIAL SCIENCES. 2018;41(2):101-9.

689. NTT Phuong, VTN Ngoc, LM Linh, NM Duc, NT Tra, LQ Anh. Bruxism, Related Factors and Oral Health-Related Quality of Life Among Vietnamese Medical Students. International journal of environmental research and public health. 2020;17(20).

690. Nunes TC, Hirano RS, Cruz LC, Seixas A, Jean-Louis G, Fonseca VAD, et al. Self perceived memory difficulties in medical students as another symptom of anxiety. TRENDS IN NEUROSCIENCE AND EDUCATION. 2018;11:9-12.

691. NY Boo, GJ Chia, LC Wong, RM Chew, W Chong, RC Loo. The prevalence of obesity among clinical students in a Malaysian medical school. Singapore medical journal. 2010;51(2):126-32.

692. Nyamute L, Mathai M, Mbwayo A, Nyamute Linda, Mathai Muthoni, Mbwayo Anne. Quality of sleep and burnout among undergraduate medical students at the university of Nairobi, Kenya. BJPSYCH OPEN. 2021;7:S279-S.

693. O Benoit, J Foret, B Merle, A Reinberg. Circadian rhythms (temperature, heart rate, vigilance, mood) of short and long sleepers: effects of sleep deprivation. Chronobiologia. 1981;8(4):341-50.

694. O Benoit, J Foret, G Bouard, B Merle, J Landau, ME Marc. Habitual sleep length and patterns of recovery sleep after 24 hour and 36 hour sleep deprivation. Electroencephalography and clinical neurophysiology. 1980;50(5):477-85.

695. O Goyal, S Nohria, AS Dhaliwal, P Goyal, RK Soni, RS Chhina, et al. Prevalence, overlap, and risk factors for Rome IV functional gastrointestinal disorders among college students in northern India. Indian journal of gastroenterology : official journal of the Indian Society of Gastroenterology. 2021;40(2):144-53.

696. O Ikotun, EWC Lee, M Glover. Medical Students' Perspective on the Effect of Sleep Quality on Academic Performance [Letter]. Advances in medical education and practice. 2020;11:569-70.

697. O Rezaei, Y Mokhayeri, J Haroni, MJ Rastani, M Sayadnasiri, H Ghisvand, et al. Association between sleep quality and quality of life among students: a cross sectional study. International journal of adolescent medicine and health. 2017;32(2).

698. O'Flynn J, Dinan TG, Kelly JR. Examining stress: an investigation of stress, mood and exercise in medical students. Irish journal of psychological medicine. 2018;35(1):63-8.

699. OA Khairoalsindi, WK Saber, NA Althubaiti, EF Alshareef, MA Almekhlafi. Primary headache characters and coping strategies among medical students of Umm Al-Qura University in the Western Region of Saudi Arabia. Neurosciences (Riyadh, Saudi Arabia). 2018;23(4):308-13.

700. OE Salama, RM Abou El Naga. Cellular phones: are they detrimental? The Journal of the Egyptian Public Health Association. 2004;79(3):197-223.

701. OF AlButaysh, AA AlQuraini, AA Almukhaitah, YM Alahmdi, FS Alharbi. Epidemiology of irritable bowel syndrome and its associated factors in Saudi undergraduate students. Saudi journal of gastroenterology : official journal of the Saudi Gastroenterology Association. 2020;26(2):89-93.

702. Oluwole OSA, Oluwole Olusegun Steven Ayodele. Recurrent Dreams of Nigerian Undergraduates. DREAMING. 2019;29(4):339-57.

703. Oraby MI, Soliman RH, Mahmoud MA, Elfar E, Abd ElMonem NA, Oraby Mohammed, I, et al. Migraine prevalence, clinical characteristics, and health care-seeking practice in a sample of medical students in Egypt. EGYPTIAN JOURNAL OF NEUROLOGY PSYCHIATRY AND NEUROSURGERY. 2021;57(1).

704. Owens JA. Sleep loss and fatigue in medical training. Current opinion in pulmonary medicine. 2001;7(6):411-8.

705. Perotta B, Arantes-Costa FM, Enns SC, Figueiro-Filho EA, Paro H, Santos IS, et al. Sleepiness, sleep deprivation, quality of life, mental symptoms and perception of academic environment in medical students. BMC medical education. 2021;21(1):111.

706. Pikó B, Barabás K, Boda K. [Epidemiology of psychosomatic symptoms and its effect on the self-evaluation of general health in university students]. Hungary1995 1995-7-30. 1667-71 p.

707. Poorolajal J, Ghaleiha A, Darvishi N, Daryaei S, Panahi S. The Prevalence of Psychiatric Distress and Associated Risk Factors among College Students Using GHQ-28 Questionnaire. Iranian journal of public health. 2017;46(7):957-63.

708. Raybin JB, Detre TP. Sleep disorder and symptomatology among medical and nursing students. Comprehensive psychiatry. 1969;10(6):452-62.

709. Reddy IY, Nagothu RS. Academic performance depends on chronotype: Myth or reality? Indian journal of clinical anatomy and physiology. 2019;6(4):471-4.

710. Silva VM, Magalhaes JED, Duarte LL, Silva Victor Menezes, Magalhaes Joedyson Emmanuel de Macedo, Duarte Leandro Lourencao. Quality of sleep and anxiety are related to circadian preference in university students. PLOS ONE. 2020;15(9).

711. Sitticharoon C, Srisuma S, Kanavitoon S, Summachiwakij S, Sitticharoon Chantacha, Srisuma Sorachai, et al. Exploratory study of factors related to educational scores of first preclinical year medical students. ADVANCES IN PHYSIOLOGY EDUCATION. 2014;38(1):25-33.

712. SK Hull, LF DiLalla, JK Dorsey. Prevalence of health-related behaviors among physicians and medical trainees. Academic psychiatry : the journal of the American Association of Directors of Psychiatric Residency Training and the Association for Academic Psychiatry. 2008;32(1):31-8.

713. SM Ketelaar, MH Frings-Dresen, JK Sluiter. Is change in health behavior of Dutch medical students related to change in their ideas on how a physician's lifestyle influences their patient's lifestyle? International journal of adolescent medicine and health. 2014;26(4):511-6.

714. Smith JM, Mader J, Szeto ACH, Arria AM, Winters KC, Wilkes TCR. Cannabis Use for Medicinal Purposes among Canadian University Students. Canadian journal of psychiatry Revue canadienne de psychiatrie. 2019;64(5):351-5.

715. Snelling J, Sahai A, Ellis H. Attitudes of medical and dental students to dissection. United States2003 2003-3. 165-72 p.

716. Sobal J. Health protective behaviors in first year medical students. Social science & medicine (1982). 1986;22(5):593-8.

717. Sun J, Yi H, Liu Z, Wu Y, Bian J, Wu Y, et al. Factors associated with skipping breakfast among Inner Mongolia medical students in China. BMC public health. 2013;13:42.

718. Tanaka J, Uchimura N, Hashizume Y, Shirakawa S, Satomura T, Ohyama T, et al. Effects of aroma on sleep and biological rhythms. Psychiatry and clinical neurosciences. 2002;56(3):299-300.

719. Taub JM, Hawkins DR. Aspects of personality associated with irregular sleep habits in young adults. Journal of clinical psychology. 1979;35(2):296-304.

720. Wu J, Huang Z, Chen Y, Chen Y, Pan Z, Gu Y. Temporomandibular disorders among medical students in China: prevalence, biological and psychological risk factors. BMC oral health. 2021;21(1):549.

721. Xie J, Li X, Luo H, He L, Bai Y, Zheng F, et al. Depressive Symptoms, Sleep Quality and Diet During the 2019 Novel Coronavirus Epidemic in China: A Survey of Medical Students. Frontiers in public health. 2020;8:588578.

722. Zheng B, Li M, Wang KL, Lv J. [Analysis of the reliability and validity of the Chinese version of Pittsburgh sleep quality index among medical college students]. Beijing da xue xue bao Yi xue ban = Journal of Peking University Health sciences. 2016;48(3):424-8.

723. Zheng B, Wang K, Pan Z, Li M, Pan Y, Liu T, et al. [Associations between dormitory environment/other factors and sleep quality of medical students]. Zhonghua liu xing bing xue za zhi = Zhonghua liuxingbingxue zazhi. 2016;37(3):348-52.

**Table S4: Quality Assessment of all included studies**

| **Study ID** | **References** | **1. Was the study target population a close representation of the national population in relation to relevant variables?** | **2. Was the sampling frame a true or close representation of the target population?** | **3. Was some form of random selection used to select the sample, OR was a census undertaken?** | **4. Was the likelihood of nonresponse bias minimal?** | **5. Were data collected directly from the subjects (as opposed to a proxy)?** | **6. Was an acceptable case definition used in the study?** | **7. Was the study instrument that measured the parameter of interest shown to have validity and reliability?** | **8. Was the same mode of data collection used for all subjects?** | **9. Was the length of the shortest prevalence period for the parameter of interest appropriate?** | **10. Were the numerator(s) and denominator(s) for the parameter of interest appropriate?** |
| --- | --- | --- | --- | --- | --- | --- | --- | --- | --- | --- | --- |
|  | Al-mistarehi, 2019 [30] |  |  |  |  |  |  |  |  |  |  |
|  | Alfadeel, 2019 [22] |  |  |  |  |  |  |  |  |  |  |
|  | Ali, 2019 [18] |  |  |  |  |  |  |  |  |  |  |
|  | Almansour, 2020 [35] |  |  |  |  |  |  |  |  |  |  |
|  | Alqudah, 2019 [28] |  |  |  |  |  |  |  |  |  |  |
|  | Alrashed, 2021 [23] |  |  |  |  |  |  |  |  |  |  |
|  | Alshaaer, 2012 [26] |  |  |  |  |  |  |  |  |  |  |
|  | Alzahrani, 2021[36] |  |  |  |  |  |  |  |  |  |  |
|  | Burhan, 2019 [32] |  |  |  |  |  |  |  |  |  |  |
|  | Essangri, 2021[31] |  |  |  |  |  |  |  |  |  |  |
|  | Goweda, 2020 [37] |  |  |  |  |  |  |  |  |  |  |
|  | Ishaq, 2020 [33] |  |  |  |  |  |  |  |  |  |  |
|  | Khan, 2019 [16] |  |  |  |  |  |  |  |  |  |  |
|  | Khurshid, 2021[17] |  |  |  |  |  |  |  |  |  |  |
|  | Mansour, 2016 [24] |  |  |  |  |  |  |  |  |  |  |
|  | Mohamed, 2020 [21] |  |  |  |  |  |  |  |  |  |  |
|  | Pervez, 2021[14] |  |  |  |  |  |  |  |  |  |  |
|  | Ram, 2016 [20] |  |  |  |  |  |  |  |  |  |  |
|  | Shakeel, 2019 [19] |  |  |  |  |  |  |  |  |  |  |
|  | Shalash, 2015 [34] |  |  |  |  |  |  |  |  |  |  |
|  | Yassin, 2020 [29] |  |  |  |  |  |  |  |  |  |  |
|  | Zainab, 2020 [15] |  |  |  |  |  |  |  |  |  |  |

|  | High ROB |
| --- | --- |
|  | Low ROB |

**Reference list**

1. Sateia MJ. International classification of sleep disorders-third edition: highlights and modifications. Chest. 2014;146(5):1387-94.

2. Hypersomnia Foundation. International Classification of Sleep Disorders (ICSD) 2022 [Available from: <https://www.hypersomniafoundation.org/glossary/international-classification-of-sleep-disorders/#:~:text=The%20ICSD%2D3%20groups%20sleep,)%20sleep%2Drelated%20movement%20disorders>.

3. American Academy of Sleep Medicine. The International Classification of Sleep Disorders – Third Edition (ICSD-3). 3 ed: American Academy of Sleep Medicine,. 2014.

4. American Sleep Association. Sleep Disorders – ICD-10 Codes and Names. 2015.

5. Singh S CS, Matchar DB, EB Bass,. Grading a Body of Evidence on Diagnostic Tests. Chang SM, Matchar DB, Smetana GW, et al, editors Methods Guide for Medical Test Reviews [Internet]. Rockville (MD): Agency for Healthcare Research and Quality (US); 2012.

6. Turner RM, Bird SM, Higgins JP. The impact of study size on meta-analyses: examination of underpowered studies in Cochrane reviews. PLoS One. 2013;8(3):e59202.

7. Higgins JPT, Thompson SG, Deeks JJ, Altman DG. Measuring inconsistency in meta-analyses. BMJ. 2003;327(7414):557-60.

8. Schwarzer G. General Package for Meta-Analysis.: CRAN; 2018. Available from: <https://github.com/guido-s/meta>

<http://meta-analysis-with-r.org>.

9. Cochrane Handbook for Systematic Reviews of Interventions. Version 6.22021.

10. Higgins JPT, Green S (editors). 9 Analysing data and undertaking meta-analyses > 9.6 Investigating heterogeneity > 9.6.4 Meta-regression. 2011. In: Cochrane Handbook for Systematic Reviews of Interventions Version 510 [updated March 2011] [Internet]. The Cochrane Collaboration. Available from: Higgins JPT, Green S (editors). Cochrane Handbook for Systematic Reviews of Interventions Version 5.1.0 [updated March 2011]. The Cochrane Collaboration, 2011. Available from [www.handbook.cochrane.org](file:///Users/soc2016/Library/CloudStorage/Box-Box/IPH-Internal%20Projects/Sleep%20Med%20student/Sleep_Disorders_med_Students_MENA/Journal%20Submissions/Scientific%20Reports/Revision%201/www.handbook.cochrane.org).

11. Hoy D, Brooks P, Woolf A, Blyth F, March L, Bain C, et al. Assessing risk of bias in prevalence studies: modification of an existing tool and evidence of interrater agreement. J Clin Epidemiol. 2012;65(9):934-9.

12. Dekkers OM, Vandenbroucke JP, Cevallos M, Renehan AG, Altman DG, Egger M. COSMOS-E: Guidance on conducting systematic reviews and meta-analyses of observational studies of etiology. PLOS Medicine. 2019;16(2):e1002742.

13. Terracciano L, Brozek J, Compalati E, Schünemann H. GRADE system: New paradigm. Curr Opin Allergy Clin Immunol. 2010;10(4):377-83.

14. Pervez S, Kumar H, Bai S, Kumar R, Parkash O, Avinash, et al. Prevalence of Insomnia Among Medical Students. PAKISTAN JOURNAL OF MEDICAL & HEALTH SCIENCES. 2021;15(4):1228-30.

15. Zainab S, Soomro RA, Khoso A, Qazi NA, Siddiqui S, Zainab Saima, et al. Frequency and Predictors of Sleep Disorders in Undergraduate Medical Students. JOURNAL OF THE LIAQUAT UNIVERSITY OF MEDICAL AND HEALTH SCIENCES. 2020;19(2):109-15.

16. Khan K, Waqas M, Sarwar R, Ahmad S, Faizan M, Khan K, et al. Effects of insomnia on daily performance of medical students: a cross sectional study conducted in university of Lahore, Pakistan. RAWAL MEDICAL JOURNAL. 2019;44(3):622-5.

17. Khurshid R, Majeed S, Amer L, Rana S, Ikram S, Upal S, et al. Attitudes and Reactions of Medical Students to the Dissection Room. Pak J Med Health Sci. 2021;15(5):917-9.

18. Ali A, Mehmood S, Farooq L, Arif H, Korai N, Khan M, et al. Influence of Excessive Mobile Phone Use on Anxiety and Academic Performance among Medical College Students. JOURNAL OF PHARMACEUTICAL RESEARCH INTERNATIONAL. 2019;31(6).

19. Shakeel HA, Maqsood H, Ishaq A, Ali B, Hussain H, Khan AR, et al. Insomnia among medical students: a crosssectional study. Int J Res Med Sci. 2019;7(3):893.

20. Ram D. Frequency of insomnia amongst medical students and its correlation with demographic variables. J Pak Pyschiatr Soc. 2016;14(2):26-9.

21. Mohamed E, Abdulrahim S, Sami W, Althaqib A, Alzuwayyid A, Almutiri K, et al. Insomnia and Related Anxiety Among Medical Students. J Res Med Dent Sci. 2020;8(3):198-202.

22. Alfadeel M, Alqahtani N, Alhudaib M, Almudhee S, Alghamdi A, Jadou N, et al. THE PREVALENCE OF INSOMNIA AMONG FEMALE MEDICAL STUDENTS OF ALMAAREFA COLLEGES IN RIYADH CITY -KINGDOM OF SAUDI ARABIA 2015-2016. INDO AMERICAN JOURNAL OF PHARMACEUTICAL SCIENCES. 2020

;6(2):3377-91.

23. Alrashed FA, Sattar K, Ahmad T, A A, Karim SI, Alsubiheen AM, et al. Prevalence of insomnia and related psychological factors with coping strategies among medical students in clinical years during the COVID-19 pandemic. Saudi J Bio Sci. 2021;28(11):6508-14.

24. Mansour T, Yousef M, Mansour TMA, Yousef M. Nightmares among young medical students. BIOMEDICAL RESEARCH-INDIA. 2016;27(2):437-41.

25. Al Zahrani J, Al Dossari K, Abdulmajeed I, Al Ghamdi S, Al Shamrani A, Al Qahtani N, et al. Daytime Sleepiness And Academic Performance Among Arab Medical Students. AMERICAN JOURNAL OF RESPIRATORY AND CRITICAL CARE MEDICINE. 2016;193.

26. Nour Eddin Farouq. Alshaaer﻿, Eyad Marashli﻿, Mohanad Mahgoub﻿, Ahmed Abdulhadi. Alashqae, editors. The Prevalence of Insomnia in Medical Students: Impact of Academic Performance2012.

27. Goweda R, Idris K, Bakhsh A, Mufti H, Gadhi M, Alrashed A, et al. Prevalence and associated risk factor of low back pain among medical student of Umm Al-Qura University, Makkah, Saudi Arabia: Cross-sectional study. MEDICAL SCIENCE. 2020;24(106):4359-67.

28. Alqudah M, Balousha SAM, Al-Shboul O, Al-Dwairi A, Alfaqih MA, Alzoubi KH. Insomnia among Medical and Paramedical Students in Jordan: Impact on Academic Performance2019 2019. 7136906 p.

29. Yassin A, Al-Mistarehi AH, Beni Yonis O, Aleshawi AJ, Momany SM, Khassawneh BY. Prevalence of sleep disorders among medical students and their association with poor academic performance: A cross-sectional study. Ann Med Surg (Lond). 2020;58(58):124-9.

30. Al-mistarehi A, Ibnian A, Shaqadan S, Khassawneh B, Al-mistarehi AW, Ibnian AM, et al. The Impact of Sleep Disorders on Academic Performance Among Medical Students. Am J Respir Crit Care Med. 2019;199:A4296.

31. Essangri H, Sabir M, Benkabbou A, Majbar MA, Amrani L, Ghannam A, et al. Predictive Factors for Impaired Mental Health among Medical Students during the Early Stage of the COVID-19 Pandemic in Morocco. Am J Trop Med Hyg. 2021;104(1):95-102.

32. Burhan NM. Prevalence of sleep disorders among medical students at King Abdulaziz University: a cross-sectional study. 2019.

33. Ishaq M, Riaz SU, Iqbal N, Siddiqui S, Moin A, Sajjad S, et al. Prevalence of Restless Legs Syndrome among Medical Students of Karachi: An Experience from a Developing Country. Sleep Disord. 2020;2020:7302828.

34. Shalash AS, Elrassas HH, Monzem MM, Salem HH, Abdel Moneim A, Moustafa RR. Restless legs syndrome in Egyptian medical students using a validated Arabic version of the Restless Legs Syndrome Rating Scale. Sleep Med. 2015;16(12):1528-31.

35. Almansour A, AlJammaz F, Ahmeda A, Alfawaz M, Abdulsalam K, AlSheikh A, et al. The Prevalence of Sleep Deprivation and its influence on Students' Life Attending Medical School at King Saud University. INTERNATIONAL JOURNAL OF PHARMACEUTICAL AND PHYTOPHARMACOLOGICAL RESEARCH. 2020;10(5):149-56.

36. Alzahrani M, Ahmed S, Sami W, Al-jubairi N, Almutairi A, Alqhatani A, et al. Prevalence of Sleep Pattern and its Effect on Health among Medical Students in Majmaah, Saudi Arabia. ANNALS OF MEDICAL AND HEALTH SCIENCES RESEARCH. 2021;11(3):1284-9.

37. Abdelmoaty Goweda R, Hassan-Hussein A, Ali Alqahtani M, Janaini MM, Alzahrani AH, Sindy BM, et al. Prevalence of sleep disorders among medical students of Umm Al-Qura University, Makkah, Kingdom of Saudi Arabia. J Public Health Res. 2021;9(s1)(Suppl 1):2020.
